# Mechanical stress-mediated immune and inflammatory regulation: **a** bibliometric and visualization analysis of mechanoimmunology based on two databases

**DOI:** 10.3389/fmed.2025.1698177

**Published:** 2025-11-06

**Authors:** Jiayuecheng Pang, Yicheng Ma, Hongchao Huang, Jiqing Ma, Yongjun Zheng, Shichu Xiao, Zhaofan Xia

**Affiliations:** 1 Department of Burn Trauma and Wound Repair, The First Affiliated Hospital of Naval Medical University, Naval Medical University, Shanghai, People's Republic of China; 2 Department of Vascular Surgery, The First Affiliated Hospital of Naval Medical University, Naval Medical University, Shanghai, People's Republic of China; 3 Research Unit of Key Techniques for Treatment of Burns and Combined Burns and Trauma Injury, Chinese Academy of Medical Sciences, Shanghai, People's Republic of China

**Keywords:** mechanical stress, biomechanics, mechanoimmunology, immune regulation, inflammatory regulation, bibliometric

## Abstract

**Background:**

With advances in detection technologies and the trend of interdisciplinary integration, mechanical stress in biomechanics has been shown to exert broad effects on organisms. It influences multiple cellular processes, including proliferation, migration, and differentiation, and also plays a critical regulatory role in both immune cell function and the organismal immune-inflammatory response. Thus, an emerging interdisciplinary field—mechanoimmunology—has emerged. However, the specific mechanisms underlying this field remain incompletely elucidated. Herein, this study aims to identify the latest research hotspots and provide a thorough analysis of the current research status in this domain.

**Methods:**

A total of 1,901 articles were retrieved from the Web of Science Core Collection (WOSCC) and 2,954 articles from Scopus. These selected articles were imported into bibliometric analysis software for preliminary data processing, followed by further visual analysis using multiple graphing tools. Finally, specific analyses were conducted based on various categories, including journals, authors, geographical distribution, citations, and keywords.

**Results:**

The United States and China are the two leading research hubs in this field, with the University of California System currently standing as the most influential institution. A surge in research on mechanoimmunology has been observed since 2021. During the “Research Explosion Phase” post-2021, several new keyword clusters emerged, including “T cells,” “macrophage polarization,” and “ion channels,” alongside clusters related to emerging fields such as “wound healing,” “gut microbiota,” and “bone remodeling.” The mechanosensitive ion channel protein Piezo1 and the YAP/TAZ transcription complex in the Hippo pathway have emerged as key targets in recent studies.

**Conclusion:**

This study identifies the ongoing research surge in the field of mechanoimmunology while comprehensively delineating the current research landscape. Additionally, through keyword clustering across different phases, it uncovers the latest research hotspots and potential future research directions focused on novel mechanosensitive targets for immune-inflammatory modulation. This work provides a clear research roadmap for investigators in the field, offering insights into future research possibilities.

## 1 Introduction

Mechanical stress is a fundamental component of biomechanics, as nearly all organisms exhibit regulated biological behaviors in response to mechanical stress. Within organisms, mechanical stress exists in multiple forms: at the cellular level, vascular endothelial cells are chronically exposed to shear stress from blood flow—this stress sustains endothelial cell function; meanwhile, tensile forces between cells influence the three-dimensional folding of cell populations (1, 2). At the tissue level, mechanical stress promotes bone maturation and bone remodeling, whereas prolonged lack of mechanical stimulation causes bone metabolic disorders (3). At the organ level, the heart is subjected to cyclic mechanical stress generated by circulation, and these forces also have profound impacts on the maintenance of cardiac function (4). The ability of mechanical stress to affect organisms relies on specialized structures including mechanical sensors, mechanosensitive ion channels, and the cytoskeleton. For instance, within intercellular adherens junctions, the mechanical linkage between adjacent structures is formed by the E-cadherin–catenin complex, while α-catenin connects the cytoskeleton to adherens junctions—these structures sense and transduce mechanical stress (5). Mechanically gated ion channels, upon mechanical stimulation, permits the flow of ions (e.g., Na^+^, K^+^, Ca^2^^+^, Cl^−^) across the cell membrane, with several such channel families having been extensively studied. The Piezo protein family is a classic example: when mechanical force is sensed, Piezo1 undergoes conformational changes and opens its central ion-conducting pore, enabling cation influx—primarily Ca^2^^+^. Similar to Piezo1, Piezo2-mediated Ca^2^^+^ influx activates RhoA, which in turn regulates the assembly of stress fibers and focal adhesions (6, 7). Additionally, the transmembrane channel-like (TMC) protein family is proposed to be involved in mechanical signal transduction (8, 9). Beyond these mechanisms, mechanical stress also controls cell biological activities by modulating signaling pathways. Cumulative studies have established that mechanical stress regulates cell proliferation and apoptosis via the Hippo/YAP signaling pathway and the Wnt/β-catenin signaling pathway (10, 11).

Immune regulation refers to the process by which the organism modulates immune function through multiple mechanisms to maintain immune homeostasis—ensuring the immune system defends against pathogen invasion while preventing excessive immune responses from causing self-tissue damage. Inflammatory regulation involves the precise modulation of the initiation, progression, intensity, and resolution of inflammatory responses within biological organisms. These two processes are tightly interconnected and mutually influential. At the cellular level, a variety of immune cells are involved in inflammatory regulation: regulatory T cells (Tregs), for instance, are capable of suppressing both immune and inflammatory responses. Tregs can regulate the expression of cytokines such as IL-10 and TGF-β to exert an inhibitory effect on immune inflammation (12). At the molecular level, cytokine regulation constitutes a key component of immune and inflammatory control. Proinflammatory cytokines such as TNF-α and IL-1β initiate and amplify inflammatory responses, whereas anti-inflammatory cytokines such as IL-10 and TGF-β dampen the intensity of inflammation and promote its resolution. Additionally, the regulation of immune-related gene expression plays a critical role: transcription factors such as T-bet and GATA-3, for example, are involved in controlling the differentiation of T cells into distinct subsets (13). These transcription factors bind to the promoter regions of specific genes to regulate their transcription and expression. Epigenetic regulation—including DNA methylation, histone modification, and non-coding RNA-mediated regulation—also contributes to this process. Dysregulation of immune and inflammatory control often leads to tissue damage, organ dysfunction, and the development of autoimmune diseases. In autoimmune hepatitis, for instance, the imbalance between Th17 cells and Tregs is implicated in disease pathogenesis and progression (14). In rheumatoid arthritis, IL-6 activates the JAK-STAT signaling pathway, leading to the phosphorylation of STAT3; this, in turn, regulates the expression of inflammation-related genes and thereby promotes inflammatory responses and joint damage (15). Beyond these, additional mechanisms of immune-inflammatory dysregulation have been identified, including abnormalities in immune checkpoints such as CTLA-4 and epigenetic alterations in relevant genes (16).

Recent studies have revealed that mechanical stress also participates in the regulation of immune inflammation in organisms. Mechanical stress is involved in the activation and polarization of immune cells: for instance, appropriate mechanical stress can induce the nuclear localization of NFAT1 in resting CD8^+^ T cells and increase c-Jun expression, which in turn promotes the expression of the early activation marker CD69 to trigger lymphocyte activation (17). In Drosophila larvae, mechanical stress induces the expression of the Unpaired 3 (Upd3) cytokine; via activation of the JAK/STAT signaling pathway, this induction elicits cellular immune responses in the larvae. Mechanical stress also contributes to inflammatory regulation (18). In oral mucosal cells, for example, mechanical stress activates the p38 MAPK cascade to modulate the production of proinflammatory cytokines (19). Additionally, mechanical stress may influence immune cell migration. As a leading researcher in the field of immunology, Professor Huse Morgan has provided in-depth insights into the mechanical regulatory mechanisms within the immune system in recent years. For instance, the mechanical forces exerted on the extracellular matrix (ECM) by the cytoskeletal remodeling of leukocytes play a pivotal role in behaviors such as immune cell activation and cell migration. Additionally, in his recently published research, he has also elaborated on how nanonewton-scale mechanical forces at the immunological synapse can influence the regulation of lymphocyte cytotoxicity mechanisms (20, 21). Within the tumor microenvironment, for instance, cancer cells upregulate canonical immune evasion mechanisms in response to mechanical stress, including epithelial-mesenchymal transition (EMT) and autophagy (22). Meanwhile, extracellular vesicles (EVs)—key mediators of intercellular communication—also play a role in mechanical stress-mediated immune regulation. Endothelial cells, for example, secrete laminar shear stress-induced EVs in response to blood flow-derived laminar shear stress; these EVs can target the MAPK signaling pathway, thereby participating in the M2 polarization of macrophages (23). Recent research on the regulation of immune inflammation by mechanical stress has experienced explosive growth. Developing novel therapeutic strategies by modulating mechanical stress appears to be a promising approach—such as utilizing biomaterials with specific mechanical properties, or applying microfluidic technology or ultrasound technology to balance mechanical forces and immune inflammation. Mechanoimmunology has emerged as a natural response to this need. However, further in-depth studies are still required to elucidate the underlying mechanisms in this field. Therefore, this study employs bibliometric analysis to focus on the developmental history and cutting-edge directions of this domain, aiming to provide novel insights for future research on mechanical stress-mediated immune and inflammatory regulation.

## 2 Materials and methods

### 2.1 Data collection and verification

The WOSCC database was selected as the main analysis set for this study, while the Scopus database served as the validation set. This setup was chosen primarily because most current bibliometric software is more compatible with data exported from the WOSCC database, whereas data exported from the Scopus database suffers from issues such as partial information loss and the need for format conversion. Furthermore, due to its stringent inclusion criteria, the WOSCC better reflects the academic impact of articles, and its citation data holds greater reference value for this study. In contrast, Scopus, with its broader coverage and greater inclusivity, may exhibit a significant increase in the citation count of the same article. Consequently, content based on citation data for analysis will exclusively use WOSCC database in this bibliometric study.

In formulating the search strategy, three key criteria were prioritized: high relevance between the strategy and search results, comprehensiveness of search outcomes, and avoidance of excessive search filters. An overabundance of filters would introduce significant subjective bias into the “Keywords” category during subsequent analyses. Therefore, the search strategy designed for this study in WOSCC database was structured as follows: terms representing mechanical stress included [TS = (mechanical stress) OR TS = (shear stress) OR TS = (mechanical strain) OR TS = (mechanical force) OR TS = (mechanical stimulation) OR TS = (mechanoimmunology) OR TS = (mechanotransduction)]; terms representing immune and inflammatory regulation included [TS = (immuneregulation) OR TS = (immune regulation) OR TS = (immune control) OR TS = (inflammatory regulation) OR TS = (inflammatoryregulation)], The two terms were connected using the AND operator. In the WOSCC database, Online First or Ahead of Print articles were excluded from this study. The citation index Editions were set to All, and article publication dates were restricted to before August 1, 2025. The same strategy was used in the Scopus database, with the category tag “TITLE-ABS-KEY” applied before the keywords. This search strategy was developed by two researchers with expertise in the relevant field. The two researchers independently formulated and executed their respective search strategies, followed by cross-validation of the strategies.

### 2.2 Inclusion and exclusion criteria

**Figure 1:**
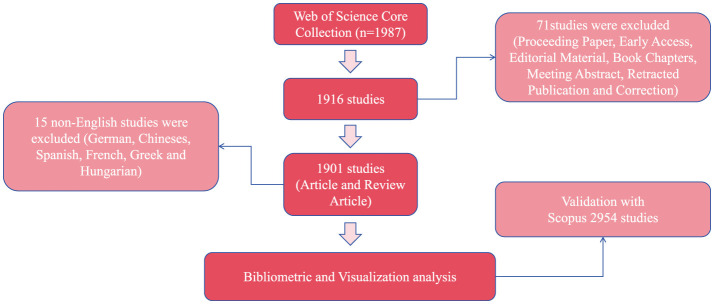
Flow chart.

Using the aforementioned search strategy, a total of 1,987 literatures were initially retrieved. After excluding 71 records of non-target document types—including Proceeding Papers, Early Access articles, Editorial Materials, Book Chapters, Meeting Abstracts, Retracted Publications, and Corrections—only 1,916 documents (comprising Articles and Review Articles) were retained. Subsequently, 15 non-English articles were further excluded, including six German-language, four Chinese-language, two Spanish-language, one French-language, one Greek-language, and one Hungarian-language records. This resulted in a final set of 1,901 English-language articles. All selected records were exported in the “Full Record and Cited References” format, encompassing 1,426 Articles and 475 Review Articles. The same inclusion and exclusion criteria were applied in the Scopus database, ultimately yielding 2,954 articles, consisting of 2,222 research articles and 732 review articles (Figure 1).

### 2.3 Data analysis

Following the final inclusion of studies, all eligible records were processed using specialized analytical tools, and subsequent analyses were conducted both separately and in combination across eight major categories: Overview, Journal, Author, Affiliation, Country, Article, Reference, and Keywords. To ensure comprehensive and rigorous data processing, visualization, and statistical analysis, four key software tools were employed in this study, each with distinct but complementary roles. Bibliometrix (R package, version 5.1.0) served as the primary tool for data processing, responsible for conducting statistical analysis and preliminary evaluation of the files exported from the WOSCC database (24). For graphical processing and visualization, two dedicated tools were utilized: Citespace (version 6.4 R1) was primarily applied to perform information clustering and generate timeline-based visualizations, which helped illustrate the evolutionary trajectory of research topics over time, while Vosviewer (version 1.6.20) was used to conduct co-occurrence analysis and co-citation analysis, enabling the identification of key research clusters and influential literature (25, 26). Additionally, GraphPad Prism (version 10.4.2) was employed for further refinement of processed data and the generation of high-quality graphs, ensuring that the final data presentations met the standards for clarity and reproducibility required for academic publication.

## 3 Results

### 3.1 Annual publications and average citations

**Figure 2:**
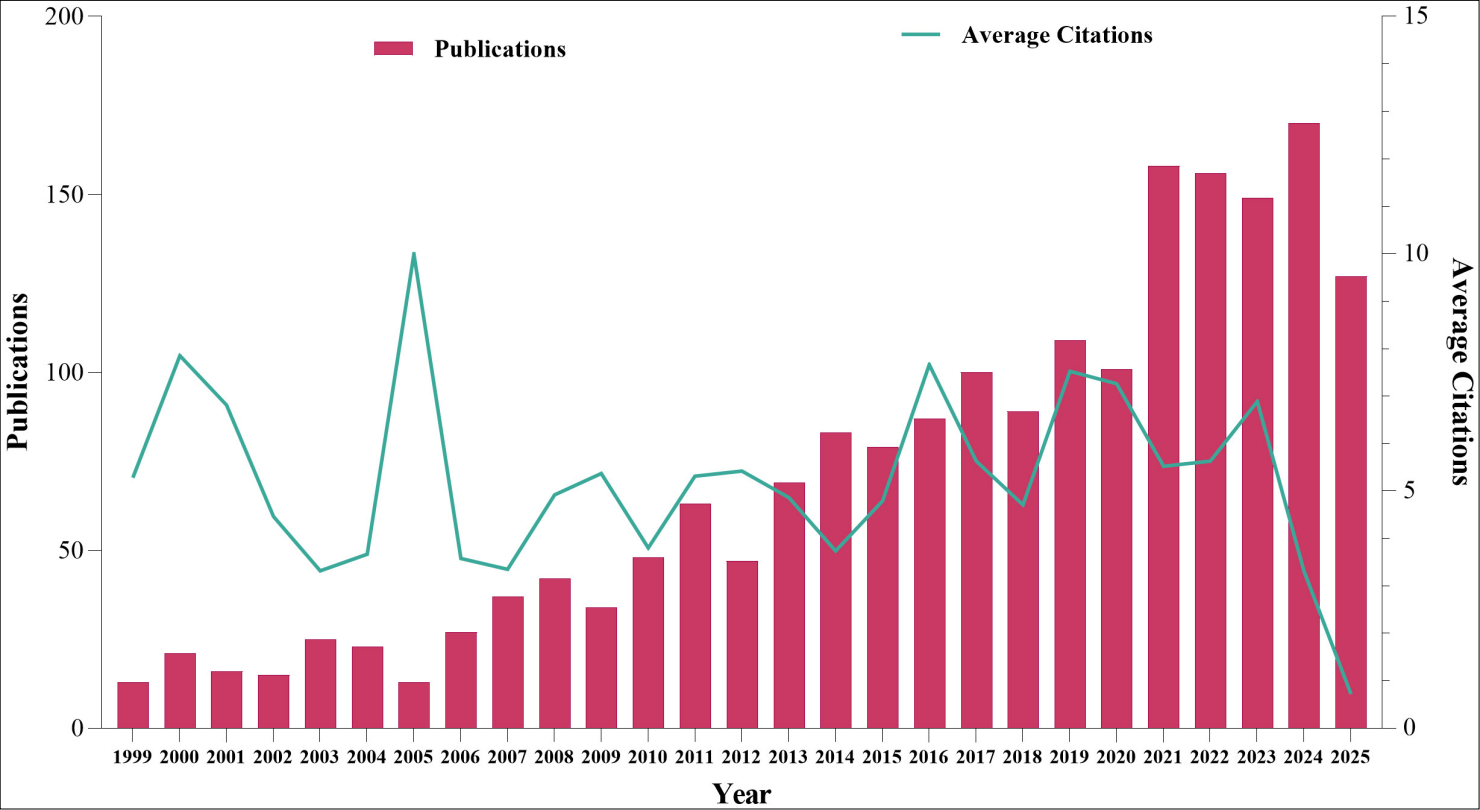
Annual publication volume and average citation count in the research field of mechanoimmunology.

Based on the established search strategy and criteria, a total of 1,901 articles in WOSCC focusing on mechanoimmunology were finally included, covering the time period from 1999 to 2025. The overall number of published articles showed a steady upward trend over this period. Notably, the largest year-on-year growth (56.44%) was observed in 2021, and the highest annual publication volume of 170 articles was reached in 2024. The same trend was also observed in the Scopus database, with sequential growth rates of 23.21 and 21.26% recorded between 2020 and 2021, representing the highest levels in history. Additionally, the number of published articles reached a peak of 261 in 2024 (Supplementary Table S1). This upward trajectory indicates that research in the field of mechanoimmunology has now entered an active phase. Interestingly, although only 13 articles were published in 2005—far fewer than the peak in later years—this year exhibited the highest average annual citation count of 209.77 citations per article in WOSCC. This observation suggests that 2005 may have witnessed the publication of either a single high-impact study or multiple influential works that have continued to shape subsequent research in this domain (Figure 2).

### 3.2 Sources

**Figure 3:**
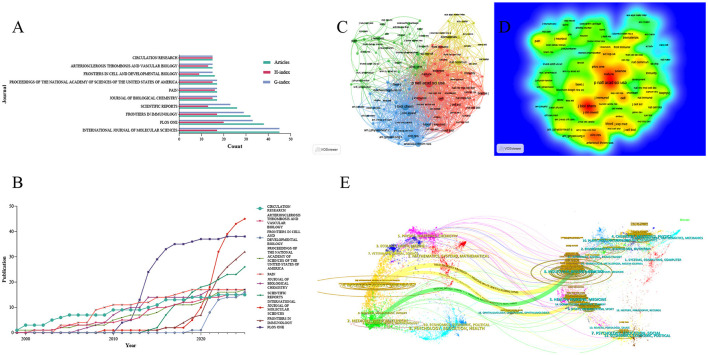
Influential journals in the research field of mechanoimmunology. **(A)** Publication volume, H-index, and G-index of the top 10 journals. **(B)** Annual publication volume of the top 10 journals. **(C)** Co-citation network map of influential journals. **(D)** Density map of influential journals. **(E)** Dual-map overlay analysis.

The top 10 journals in the field of mechanoimmunology were identified based on Bradford's Law. Among these journals, *International Journal of Molecular Sciences* had the highest number of publications and the highest G-index: it published 45 articles, with an H-index of 17 and a G-index of 45. This finding indicates that although this journal only has 17 highly cited papers, there are a small number of studies with extremely high citation counts in its published works, which have significantly boosted the total cumulative citations. For example, two articles focusing on vascular endothelial cell biology and the pathophysiology of atherosclerosis have been cited a total of 1,286 times. This also indicates that the relationship between hemodynamics in cardiovascular diseases and immune-inflammatory regulation may be a critical component of research in this field (27, 28). *PLoS One* ranked highest in terms of H-index (H-index = 20), while also having 38 publications and a G-index of 33. These metrics demonstrate that this journal carries substantial weight in the relevant field, with its papers excelling in both quantity and quality. Additionally, *Frontiers in Immunology* deserves attention as it had the highest M-index (M-index = 1.417). This high M-index suggests that *Frontiers in Immunology* is the journal with the highest impact per unit time in the field of mechanoimmunology, and has garnered greater attention in this domain in recent years (Figures 3A, B, Table 1). The same pattern was observed in the Scopus database, where *International Journal of Molecular Sciences, Frontiers in Immunology*, and *PLoS One* remained the top three journals in terms of influence in this field. Various indices for each journal, calculated based on Scopus data, are provided in Supplementary Table S2.

**Table 1:** Top 10 sources in the research field of mechanoimmunology.

**Source**	**Publications**	**Total citations**	**H-index**	**G-index**	**M-index**	Impact factor^*^	JIF category/quartile^*^
International Journal of Molecular Sciences	45	2,117	17	45	1	4.9	Biochemistry and Molecular Biology/Q1
PLoS One	38	1,134	20	33	1.333	2.6	Multidisciplinary Sciences/Q2
Frontiers in Immunology	32	862	17	29	1.417	5.9	Immunology/Q1
Scientific Reports	26	572	13	23	1.083	3.9	Multidisciplinary Sciences/Q1
Journal of Biological Chemistry	17	1,101	15	17	0.75	3.9	Biochemistry and Molecular Biology/Q2
Pain	17	1,599	16	17	0.727	5.5	Anesthesiology/Q1
Proceedings of the National Academy of Sciences of the United States of America (PNAS)	17	2,233	15	17	0.556	9.1	Multidisciplinary Sciences/Q1
Frontiers in Cell and Developmental Biology	16	242	9	15	1.286	4.3	Cell Biology/Q2
Arteriosclerosis, Thrombosis, and Vascular Biology	15	903	13	15	0.52	7.4	Hematology/Q1
Circulation Research	15	5,157	15	15	0.556	16.2	Cardiac and Cardiovascular Systems/Q1

^*^The content of impact factor (IF) and JIF quartile are derived from journal citation reports™ (JCR™) 2024.

To reveal the network framework of knowledge sources in the relevant field, we conducted a journal co-citation analysis. The results showed that two major clusters of core journals constitute the primary knowledge framework: one cluster is centered on high-impact journals including *PNAS, Nature*, and *Science*, while the other is dominated by journals such as *Journal of Biological Chemistry* and *Journal of Clinical Investigation* (Figures 3C, D). In parallel, we performed an analysis of the journal dual-map overlay to investigate the cross-domain citation relationships between citing literature and cited literature. This analysis revealed that research on mechanical stress-mediated immune and inflammatory regulation is concentrated in two major disciplinary categories: “Molecular, Biology, Immunology” and “Medicine, Medical, Clinical.” Notably, both categories share a common knowledge base derived from the “Molecular, Biology, Genetics” field. Specifically, the “Immunology” subcategory within the first major category cited literature from the “Genetics” field 37,526 times, with a standardized Z-score of 10.039594. In contrast, the “Clinical” subcategory within the second major category cited the “Genetics” field 9,832 times, corresponding to a standardized Z-score of 2.46391 (Figure 3E).

### 3.3 Author

**Figure 4:**
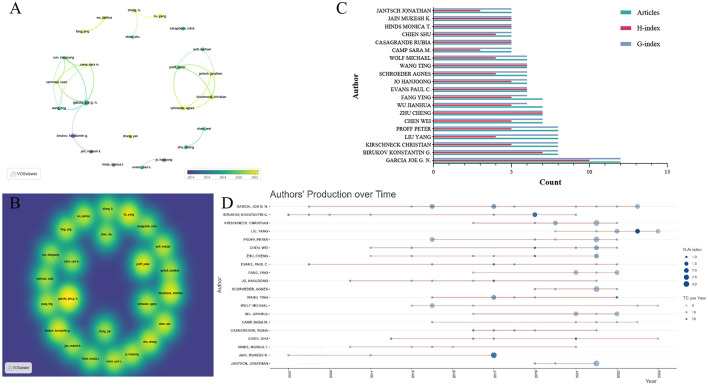
Influential authors in the research field of mechanoimmunology. **(A)** Co-author network map over time. **(B)** Co-author density map. **(C)** Publication volume, H-index, and G-index of the top 20 authors. **(D)** Production of the top 20 authors over time.

The top 20 authors in the field were selected based on their number of publications. Among these authors, Garcia Joe GN emerged as the most influential figure: he has conducted research in this field since 2008, with a total of 12 published articles, and his H-index (H-index = 10) and G-index (G-index = 12) are the highest among all included authors—these metrics collectively confirm his leading status in the domain. In the Scopus database, exactly the same result was also obtained—Garcia Joe GN identified as the most influential author (Supplementary Table S3). Liu Yang, by contrast, represents the most active emerging author in recent years in WOSCC. Among the top 10 authors ranked by publication count, Liu Yang had the highest M-index (M-index = 0.667). This observation is further supported by the author publication timeline: Liu Yang entered the field in 2020 and became the author with the highest average annual citation count in 2024, achieving an average of 29.5 citations per article that year (Supplementary Table S4). To gain deeper insights into collaborative dynamics, we further performed an author collaboration network analysis. This analysis revealed the formation of two major collaborative clusters: one centered on Garcia Joe GN and the other on Proff Peter. These findings indicate that both Garcia Joe GN and Proff Peter are pivotal leading researchers in this field (Figures 4A–D).

### 3.4 Regional distribution

**Figure 5:**
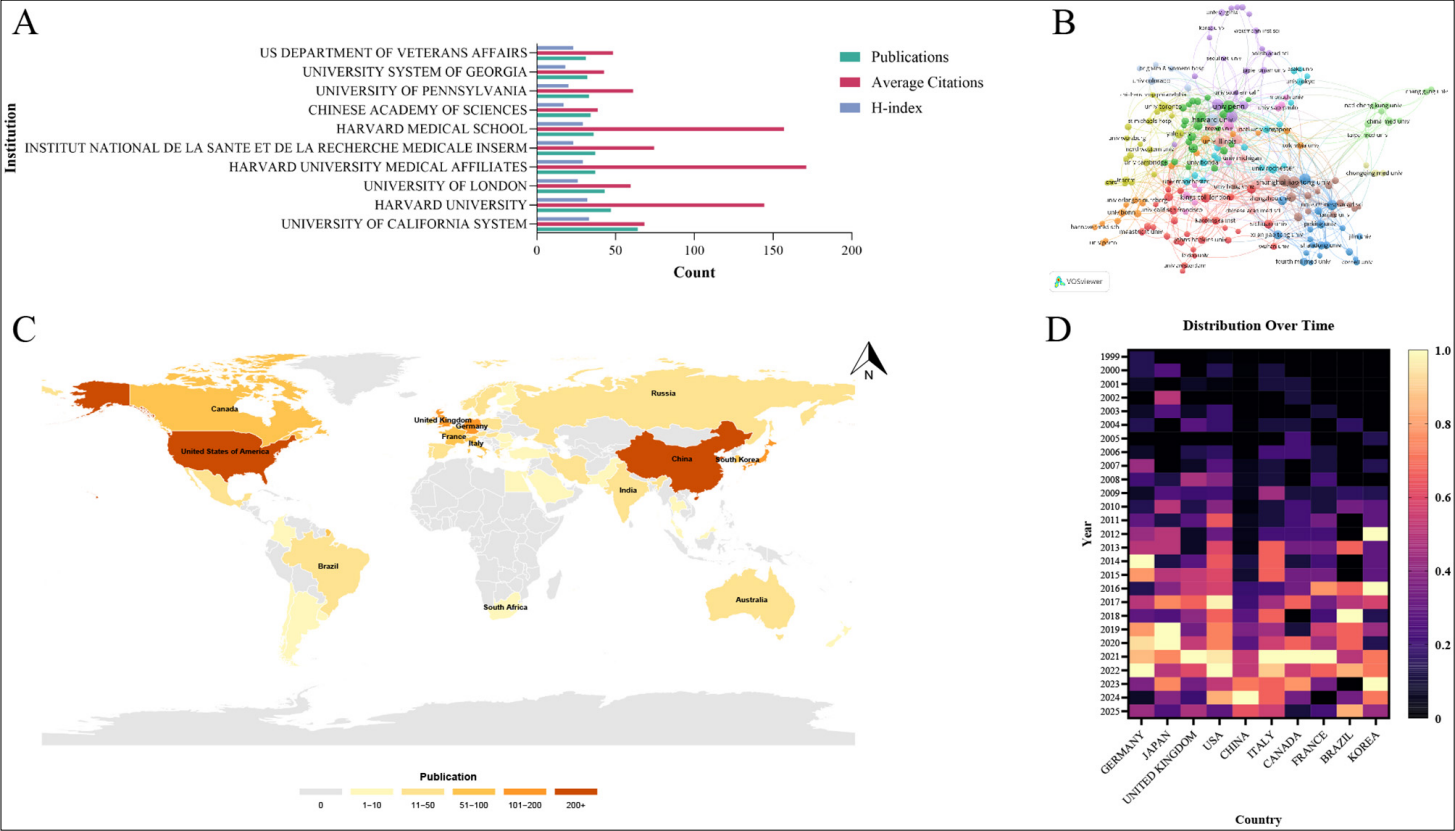
Influential institutions and countries in the research field of mechanoimmunology. **(A)** Publication volume, average citations, and H-index of the top 10 institutions. **(B)** Co-author network map of influential institutions. **(C)** World map of publication output by influential countries. **(D)** Distribution of independent standardized annual publication volumes among the top 10 countries.

To characterize regional research strengths and inter-regional collaborative relationships in the field of mechanoimmunology, we conducted a regionalized analysis of the affiliations of first authors of all included papers and the countries where these affiliations are located. Among all institutions, the University of California System ranked first in terms of publication count, with 64 articles published, and also had the highest H-index (H-index = 33). These metrics collectively indicate that the University of California System is the most influential institution in the relevant research field. In contrast, Harvard University Medical Affiliates, despite publishing 37 articles (fewer than the University of California System), achieved the highest average citations per article of 171.03. This finding suggests that Harvard University Medical Affiliates has produced high-quality, or even extremely high-impact, research papers in this domain (Figure 5A). In the Scopus database, the University of California also emerged as an institution with a high volume of publications, further validating its influence in this field (Supplementary Table S5). Furthermore, an analysis of the inter-institutional collaboration network revealed the formation of two distinct collaborative structures: a major global collaboration network centered on the University of Pennsylvania and Harvard University, and a smaller regional collaboration network led by Shanghai Jiao Tong University (Figure 5B).

**Figure 6:**
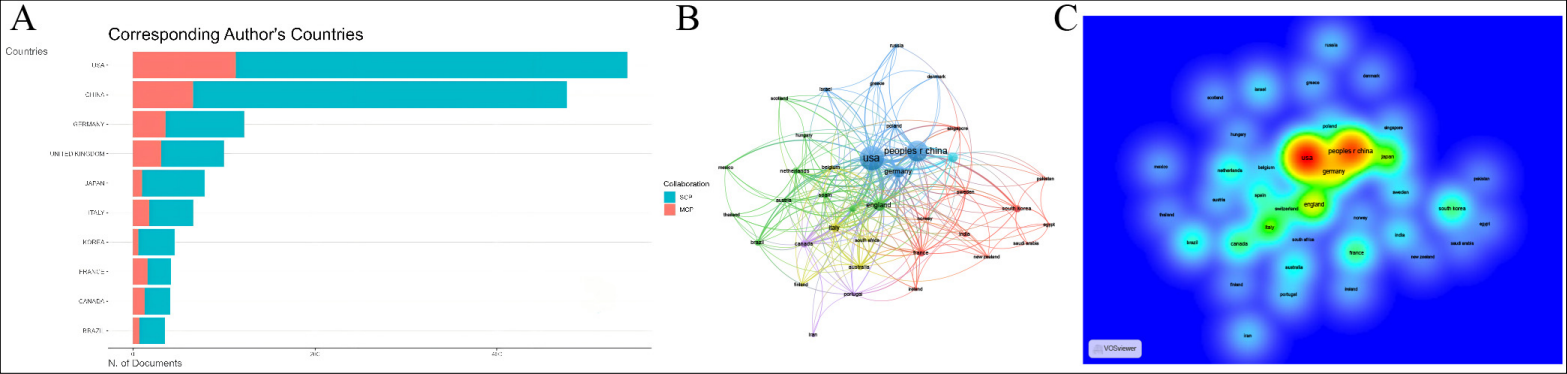
Inter-Country Collaboration. **(A)** The SCP and MCP of the top 10 countries. **(B)** Co-author network map. **(C)** Co-author density map. SCP, single-country publications; MCP, multi-country publications.

Among all countries conducting research in this field, the United States (US) and China stand out as the two most influential research hubs: the US has published 668 articles in WOSCC, while China has published 481 articles (Figure 5C). This trend was also consistent in the Scopus database. The United States ranked first with 973 publications, while China ranked second with 841 publications (Supplementary Figure S1). To explore the temporal distribution trends of research activity across countries, we analyzed the annual publication counts of each country after standardization. This analysis revealed distinct research progress trajectories between the US and China. The US has been engaged in research in this field since the early 2000s, with its annual publication count showing a steady year-on-year increase; notable peaks in research output were observed in 2017 and 2022. In contrast, China devoted limited attention to this field during its early developmental stages and only began to conduct in-depth research gradually starting from 2014. Despite this later entry, China has demonstrated a remarkable research pace: through intensive research efforts in just recent years, it has emerged as the world's second-largest research hub in this domain, with its research output peaking in 2024 (Figure 5D). An analysis of inter-country collaborative relationships further confirmed the status of the US and China as the two major research centers. Statistics on Single-Country Publications (SCP) and Multi-Country Publications (MCP) for all articles showed that the US had the highest number, which is 113, of MCP, followed by China of 66—ranking first and second globally, respectively (Figure 6A, Supplementary Table S6). It should be noted that the calculation method here is based on whether an article involves cross-country corresponding author collaboration, using the affiliations of co-corresponding authors as the benchmark. Consequently, the number of articles counted differs from the national publication count, which is statistically based on the affiliation of the first author. Additionally, the inter-country collaboration network indicated that both the US and China serve as central nodes in the global collaborative network (Figures 6B, C).

### 3.5 Documents and references

**Figure 7:**
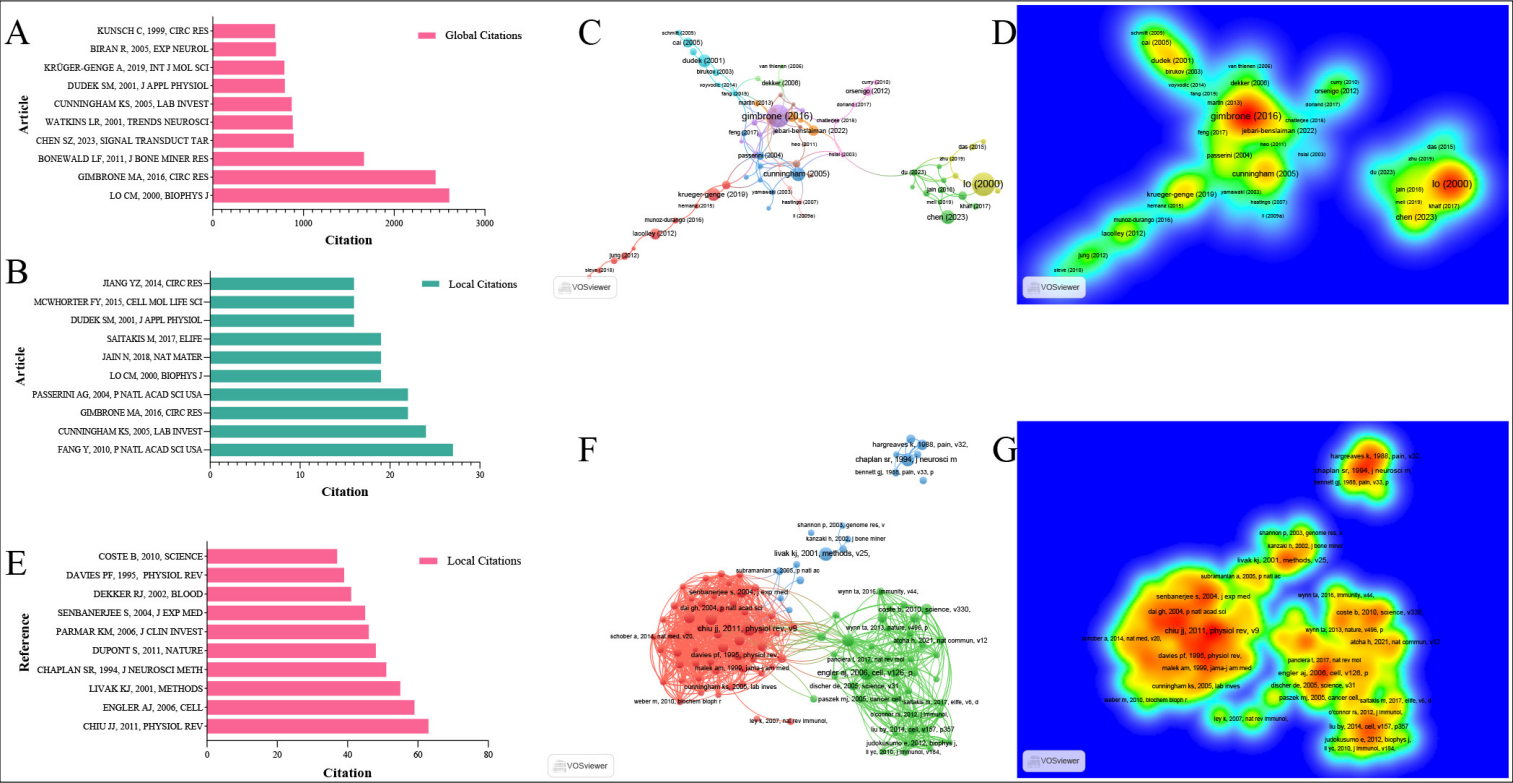
Analysis of highly cited articles and references. **(A)** Top 10 global cited articles. **(B)** Top 10 local cited articles. **(C)** Citation network map of highly cited articles. **(D)** Density map of highly cited articles. **(E)** Top 10 cited references in the research field. **(F)** Co-citation network map of highly cited references. **(G)** Density map of highly cited references.

For all included studies in WOSCC, we conducted total citation analysis and local citation analysis. Local citations refer to the number of times a study is cited by relevant literature within this field, which can reflect the relevance of the study to the domain. The article published by author LO CM in 2000 in the *Biophysical Journal* had the highest total citation count (2,607 citations), ranking first among all included studies (29). Meanwhile, this article received 19 local citations within the field, placing it fourth in local citation rankings. In contrast, the article by author Fang Y, published in 2010 in *PNAS*, achieved the highest local citation count of 27, despite a lower total citation count of 396. This discrepancy suggests that Fang Y's 2010 article may have stronger relevance to research in this specific field (30) (Figures 7A, B, Table 2). We further performed a citation network analysis of all included literatures, which revealed two distinct core nodes in the citation network. One of these cores is the 2000 article by Lo CM, which was mentioned earlier. The other major core node in the citation network is the article published by author Gimbrone MA in 2016 in *Circulation Research*—a status consistent with its total and local citation data (31). Specifically, this article ranked second in total citations of 2,457 and third in local citations of 22 (Figures 7C, D, Supplementary Table S7).

**Table 2:** The top 10 most local cited article in the research field of mechanoimmunology.

**Article title**	**First author**	**Journal**	**Local citations**	**Global citations**	**Publication year**
MicroRNA-10a regulation of proinflammatory phenotype in athero-susceptible endothelium *in vivo* and *in vitro* (30)	Fang Y	PNAS	27	396	2010
The role of shear stress in the pathogenesis of atherosclerosis (36)	Cunningham KS	Laboratory Investigation	24	871	2005
Endothelial cell dysfunction and the pathobiology of atherosclerosis (31)	Gimbrone MA	Circulation Research	22	2,457	2016
Coexisting proinflammatory and antioxidative endothelial transcription profiles in a disturbed flow region of the adult porcine aorta (79)	Passerini AG	PNAS	22	285	2004
Cell movement is guided by the rigidity of the substrate (29)	Lo CM	Biophysical Journal	19	2,607	2000
Spatial confinement downsizes the inflammatory response of macrophages (80)	Jain N	Nature Materials	19	200	2018
Different TCR-induced T lymphocyte responses are potentiated by stiffness with variable sensitivity (81)	Saitakis M	eLife	19	178	2017
Cytoskeletal regulation of pulmonary vascular permeability (82)	Dudek SM	Journal of Applied Physiology	16	795	2001
Physical and mechanical regulation of macrophage phenotype and function (48)	McWhorter FY	Cellular and Molecular Life Sciences	16	353	2015
Hemodynamic disturbed flow induces differential DNA methylation of endothelial Kruppel-Like factor 4 promoter *in vitro* and *in vivo* (83)	Jiang YZ	Circulation Research	16	158	2014

To explore the knowledge origins of mechanoimmunology, we analyzed the reference literatures of all included studies. The article published by author Chiu JJ in 2011 in *Physiological Reviews* was cited the most frequently (63 citations), followed by the 2006 article by author Engler AJ in Cell (59 citations), which ranked second (32, 33). Consistent with these citation data, the reference collaboration network revealed that these two articles serve as the core nodes of two major collaborative networks, respectively. These findings confirm that the knowledge base of this field has evolved from the knowledge networks centered on these two foundational literatures (Figures 7E–G, Table 3).

**Table 3:** The top 10 most cited reference in the research field of mechanoimmunology.

**Reference title**	**First author**	**Journal**	**Citations**	**Publication year**
Effects of disturbed flow on vascular endothelium: pathophysiological basis and clinical perspectives (32)	Chiu JJ	Physiological Reviews	63	2011
Matrix elasticity directs stem cell lineage specification (33)	Engler AJ	Cell	59	2006
Analysis of relative gene expression data using real-time quantitative PCR and the 2[-Delta Delta C(T)] method (84)	Livak KJ	Methods	55	2001
Quantitative assessment of tactile allodynia in the rat paw (85)	Chaplan SR	Journal Of Neuroscience Methods	51	1994
Role of YAP/TAZ in mechanotransduction (49)	Dupont S	Nature	48	2011
Integration of flow-dependent endothelial phenotypes by Kruppel-like factor 2 (86)	Parmar KM	Journal of Clinical Investigation	46	2006
KLF2 Is a novel transcriptional regulator of endothelial proinflammatory activation (87)	Senbanerjee S	Journal of Experimental Medicine	45	2004
Prolonged fluid shear stress induces a distinct set of endothelial cell genes, most specifically lung Krüppel-like factor (KLF2) (88)	Dekker RJ	Blood	41	2002
Flow-mediated endothelial mechanotransduction (89)	Davies PF	Physiological Reviews	39	1995
Piezo1 and Piezo2 are essential components of distinct mechanically activated cation channels (90)	Coste B	Science	37	2010

**Figure 8:**
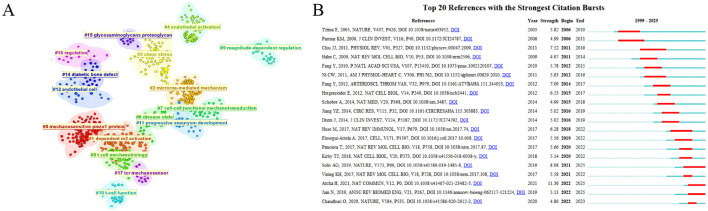
Cluster analysis and citation burst analysis of references. **(A)** Clusters of key terms in references. **(B)** Top 20 references with the strongest citation burst.

Concurrently, we performed a cluster analysis of the key terms extracted from all reference literatures, which yielded 16 major clusters covering key topics such as “mechanosensitive Piezo1 protein”, “dependent M2 activation,” and “microRNA-mediated mechanism.” These clusters enable the tracing of both the systematic knowledge foundations and the mechanistic research origins of the field (Figure 8A). Furthermore, a citation burst analysis of all reference literatures identified the article “Mechanically activated ion channel Piezo1 modulates macrophage polarization and stiffness sensing”—published in *Nature Communications* in 2021—as having the strongest citation burst index of 11.36 (34). Since 2022, this article has been cited 28 times by the studies included in our analysis, highlighting its role as a critical component of recent research in this domain (Figure 8B).

### 3.6 Keywords

**Figure 9:**
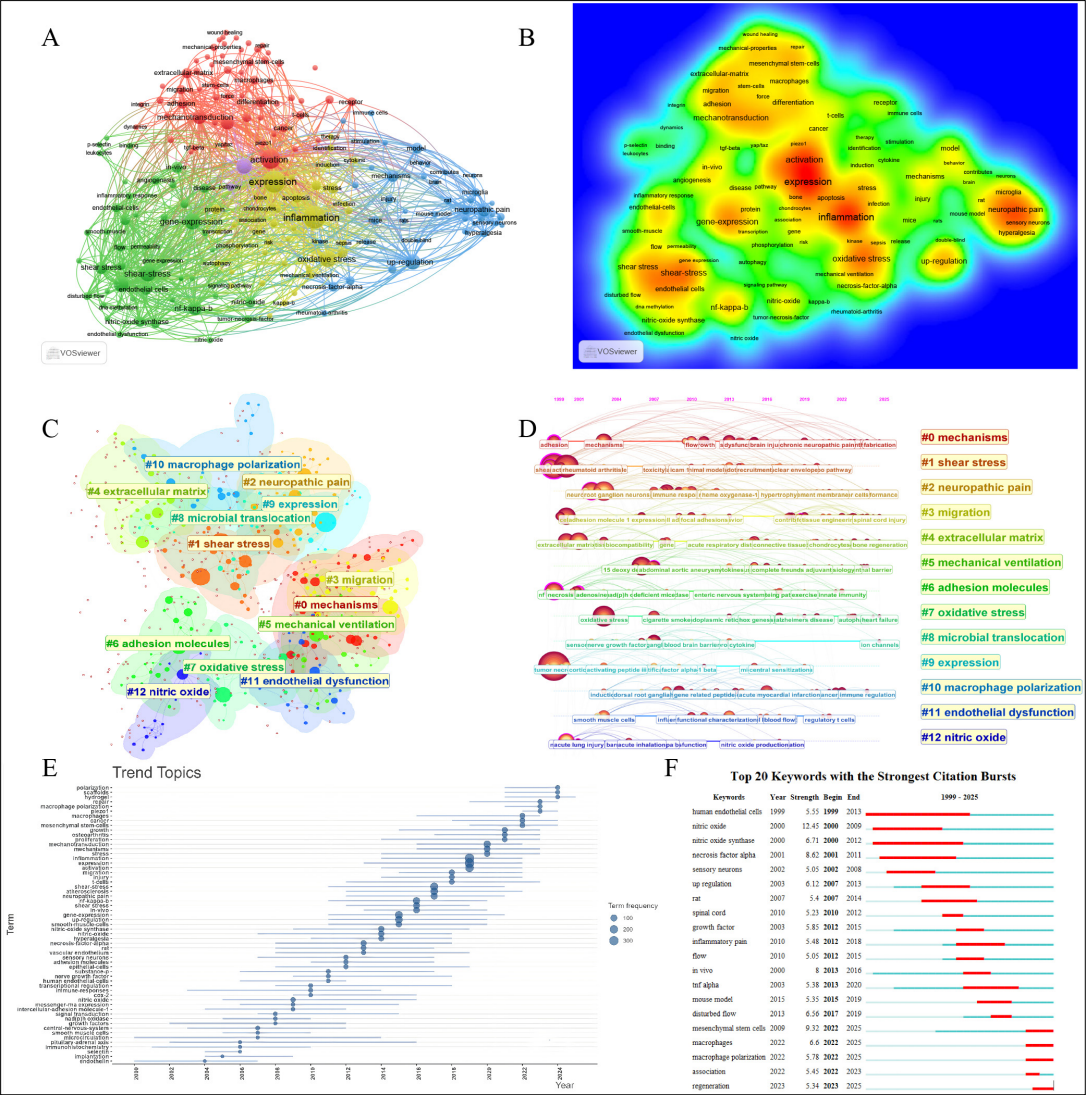
Keywords analysis. **(A)** Co-occurrence network map of keywords. **(B)** Density map of keywords. **(C)** Keywords clusters of all literatures. **(D)** Timeline view of keywords clusters. **(E)** Trend topics of keywords. **(F)** Top 20 keywords with strongest citation bursts.

Keywords analysis serves as an analytical dissection of research trends and hotspots in the field. To characterize the evolutionary changes, we generated a keyword thematic timeline based on the frequency of keyword occurrences across different time periods—this visualization facilitates the intuitive tracking of shifts in research emphasis over time. Concurrently, we performed a keyword co-occurrence analysis of all included literatures in WOSCC, which identified four major keyword co-occurrence clusters. These clusters radiate around four central keywords, respectively: “activation,” “shear stress,” “neuropathic pain,” and the combined theme of “inflammation + expression” (Figures 9A, B). Each co-occurrence cluster contains noteworthy associations: for instance, keywords such as “endothelial cells,” “shear stress,” and “NF-κB” exhibited high-frequency co-occurrence. This pattern suggests that shear stress may regulate the biological behaviors of endothelial cells through NF-κB-related cytokines or signaling pathways. For example, this study has demonstrated that shear stress induces VCAM-1 via the NF-κB pathway in vascular endothelial cells, thereby upregulating the expression of CX3CR1—a process closely associated with the development of atherosclerosis (35). Other high-frequency co-occurring keyword groups include “macrophages, mechanotransduction, activation, Piezo1, cancer” and “TNF-α, upregulation.” Keyword analysis was also conducted in the Scopus database, and the results showed that its high-frequency keywords centered on experimental method terms such as “human,” “human cell,” “article,” and “animal experiment.” This is precisely related to the relatively lenient inclusion policy of the Scopus database mentioned earlier, which leads to the inclusion of keywords from a large number of non-high-quality studies. However, in its medium-frequency keyword network, the same keyword clusters as those in the WOSCC database can still be identified, such as the cluster of “shear stress, endothelial cells, inflammation” (Supplementary Figure S2). While keyword co-occurrence analysis initially explored the research directions and hotspots in this field, we further conducted keyword clustering analysis to enable deeper excavation of latent information within the data.

Through this keyword clustering analysis of WOSCC data, a total of 13 major clusters were identified, corresponding to the following themes: “mechanisms,” “shear stress,” “neuropathic pain,” “migration,” “extracellular matrix,” “mechanical ventilation,” “adhesion molecules,” “oxidative stress,” “microbial translocation,” “expression,” “macrophage polarization,” “endothelial dysfunction,” and “nitric oxide.” Concurrently, a timeline analysis was performed on these clusters (Figures 9C, D). The specific keyword lists for each cluster in the WOSCC database, as well as the keyword clustering results from Scopus, are all available in Supplementary Figure S3A, Supplementary File S1.

In the keyword burst analysis of WOSCC data, “human endothelial cells” exhibited the longest burst duration, spanning from 1999 to 2013. Data in Scopus confirms this, with “endothelium” bursts between 2000 and 2015 (Supplementary Figure S3B). This observation indicates that research on human endothelial cells represented the most prominent focus in the early stage of this field, which corroborates the content described in the previous “Sources” section, indicating that cardiovascular disease was the primary direction of early-stage research in this field (27, 28). In contrast, “necrosis factor alpha” showed an early surge trend in both WOSCC and Scopus, indicating that TNF-α may be a key factor in early-stage research. In recent years, research in this field has gradually shifted. For instance, in WOSCC, the keyword “macrophages” has shown a burst peak with an intensity of 6.6 since 2022; similarly, “macrophage polarization” has exhibited a burst peak with an intensity of 5.78, also starting from 2022. In Scopus, “wound healing” and “hydrogel” surged rapidly after 2021, indicating that the field may currently be focusing its attention on wound repair. These data confirm that the field is currently centered on macrophage regulation and wound repair as its primary research direction (Figures 9E, F, Supplementary Figure S3B).

## 4 Discussion

### 4.1 Current status of the research field and international collaboration

Research in the field of mechanoimmunology is currently in an explosive growth phase and cardiology and cardiovascular surgery have emerged as pioneering disciplines in this research area. This is attributed to the fact that hemodynamic factors—such as blood flow shear stress, which are inherent to cardiovascular diseases—constitute a key research focus of these disciplines. Among the top 10 most highly cited papers in the field, three are derived from cardiovascular disease research. For example, a study published in *Circulation Research* (cited 2,457 times) mentions that specific fluid mechanical stimuli can regulate endothelial genes that play critical roles in proinflammatory activation (31). Another paper, with 871 citations, also notes that shear stress appears to significantly influence vascular inflammation by regulating endothelial genes to acquire atherogenic properties (36). Among the top 10 local cited articles, as many as 6 are related to cardiovascular diseases or vascular endothelial cells. It is evident that researchers in cardiology and cardiovascular surgery were among the first to recognize the involvement of mechanical forces in inflammatory regulation, making them early contributors to this field. Notably, despite only 13 articles being published in 2005, this year achieved the highest average annual citation count. This phenomenon can be attributed to the six cardiovascular disease-related articles published in 2005, which accounted for 63.99% of the total citations that year (36–41).

The substantial growth in the number of publications in 2021 can be attributed to two key factors: a shift in research focus within foundational disciplines and the rise of China as a research force in this field. In 2020, Immunology was the discipline with the highest number of publications (13 articles). By 2021, however, Biochemistry, Molecular Biology, and Cell Biology emerged as the top two disciplines in terms of publication output, with 30 and 25 articles, respectively. This trend indicates that since the 2020s, research in the field has gradually shifted toward deeper mechanistic investigations (Supplementary File S2). The potential cause of this phenomenon may be attributed to the rapid advancement of detection technologies. Over the past decade alone, the development of single-molecule force measurement and microscopic imaging techniques has enabled mechanical studies at the cellular level. For example, dsDNA probe technology has successfully measured the loading rates of integrin forces at 0.5–2 pN/s. Furthermore, dynamic super-resolution imaging technology, when combined with fluorescent dyes, allows for the monitoring of real-time mechanical forces associated with various biological activities. In addition, various modifiable and deformable hydrogel materials, when used in conjunction with techniques such as ultrasonic elastography and magnetic resonance elastography, have become common tools for investigating how changes in ECM stiffness affect cellular biological activities (42–47). Over the past 5 years, the exploration of underlying mechanisms has become a rapidly growing research hotspot. Prior to and including 2020, the US consistently ranked first in annual publication volume in this field. However, since 2021, China has overtaken the US, establishing itself as an emerging research powerhouse in the domain.

Nevertheless, distinctions remain in the international central status of the US and China in this field. The US continues to serve as a global hub for international collaboration in the domain, while China is still in the phase of expanding its influence. This discrepancy stems from the US's earlier entry into research in this field, more solid research foundations, and a greater number of research centers. As evident from the inter-institutional network diagram, a global collaborative network radiates outward with US-based institutions at its core. In contrast, Chinese institutions occupy peripheral positions in this collaborative network, and intra-country collaborations among Chinese institutions are more prominent compared to international collaborations (Figure 5B). Another country worthy of attention is Germany, which not only ranks third globally in publication count but also serves as one of the core nodes in the cross-country collaboration network. Meanwhile, Peter Proff from the University Hospital Regensburg (Germany) is a key author in international collaborations within this field. An interesting set of data reveals that East Asian countries—such as China, Japan, and South Korea—exhibit lower MCP% values, while European countries (including Germany, the United Kingdom, France, and Italy) have higher MCP% values. Combined with the cross-country and inter-institutional collaboration networks, these findings suggest that European countries maintain closer collaborative relationships in the field of mechanical stress-mediated immune and inflammatory regulation. In contrast, East Asian countries engage in relatively limited collaboration with one another and remain in a phase of relatively independent research. This phenomenon has also contributed to the slower pace of influence expansion of East Asia in this domain.

In this disciplinary field derived from biomechanics, the fundamental basis of all research lies in the observation that physical signals such as mechanical stress can influence the physiological and pathological processes of cells. A prime example is the article with the highest total citation count—“Cell Movement Is Guided by the Rigidity of the Substrate”—published by author LO CM in 2000, which found that 3T3 cells exhibit a greater tendency to migrate toward rigid substrates. Concurrently, mechanical strain generated by manipulating flexible substrates can also guide cell movement (29). Thanks to the insights gained by researchers into the regulatory role of mechanical stress, subsequent scientists have gradually uncovered that mechanical stress influences immune cells and inflammatory cells through mechanisms involving mechanical sensors and mechanosensitive ion channels. For instance, the article “Physical and Mechanical Regulation of Macrophage Phenotype and Function”—which has accumulated 353 total citations and 16 local citations—reveals the molecular mechanisms underlying macrophage mechanotransduction, with implicated molecules including integrins, FAK-Src activation, and actin polymerization (48). As previously noted, cardiology and cardiovascular surgery researchers were pioneers in this field. Analysis of the highly cited article network diagram shows that while Lo CM's 2000 article (the most highly cited overall) serves as a peripheral core node in the citation network, the more centrally located node within the entire citation network is the aforementioned article from *Circulation Research*—which explores the relationship between mechanical stimulation and endothelial cells in atherosclerotic disease (31) (Figures 7C, D). In the pathological development of atherosclerosis, mechanical stimulation and immune-inflammatory responses are nearly unavoidable factors; thus, cardiovascular research holds greater reference value for the field of mechanoimmunology. For example, the article “MicroRNA-10a Regulation of Proinflammatory Phenotype in Athero-Susceptible Endothelium *in vivo* and *in vitro*”—which has the highest number of local citations of 27—demonstrates that mechanical stimulation from blood flow promotes the proinflammatory phenotype of endothelial cells by regulating microRNA-10a (30). It is for this reason that the most frequently cited reference in this field also focuses on the pathophysiological effects of vascular endothelial cells in response to mechanical stimulation from blood flow (32).

Of greater interest, however, is the observation that among the frequently cited references in this field, there are numerous basic research articles that extend beyond the scope of vascular diseases. For instance, a 2006 article from *Cell*—which ranks second in local citation frequency (59 local citations)—found that undifferentiated mesenchymal stem cells (MSCs) are highly sensitive to tissue-level elasticity, a property that may influence the lineage commitment of these cells (33). Another notable example is a 2011 article from *Nature* (48 local citations), which directly addresses the roles of YAP and TAZ—two key markers in mechanotransduction (49). This phenomenon is more intuitively evident in the reference network diagram, where two distinct clusters of references have emerged: one centered on vascular disease research, and another independent cluster. This separate cluster consists of basic research articles that radiate outward from the 2006 *Cell* article mentioned above (Figures 7F, G).

The underlying cause of this phenomenon lies in the fact that among all articles included in the bibliometric analysis, there is a large number of recent studies published in recent years. Due to their high cutting-edge nature, these studies have not yet been frequently cited and thus do not appear in the list of highly cited articles. However, these newly published studies may cite a more specialized or precise set of references—this increases the likelihood of a distinct reference cluster emerging among the frequently cited references. As evident from the reference network diagram, the articles in this new reference cluster have relatively recent publication dates, which strongly suggests that this cluster likely represents a source of the latest knowledge system in the field. Therefore, we will integrate the insights from this section with the results of keyword analysis to discuss the shifts in research hotspots and the latest advancements.

### 4.2 Shifts in research directions and latest research hotspots

Based on the aforementioned analysis results, 2020 is defined as a turning point. The period from the inception of research up to and including 2020 is designated as the “Research Exploration Phase,” while the period from 2021 to the present is termed the “Research Explosion Phase.” The Research Exploration Phase was centered on investigating the effects of mechanical stress on endothelial cells in cardiovascular diseases—a conclusion supported by keyword burst analysis. “Human endothelial cells” emerged as a high-frequency hotspot term in early-stage research, and for example, this early study demonstrated that shear stress can inhibit the biosynthesis of TNF-α via heme oxygenase-1 in human endothelial cells, thereby suppressing inflammatory responses (50). Additionally, one study has also demonstrated that endothelial cells exposed to high hemodynamic energy may induce more significant expression of inflammatory cytokines (51). All these researches indicating that mechanical stress regulates the immune-inflammatory responses of vascular endothelial cells. Additionally, TNF-α was the most extensively studied cytokine during the Research Exploration Phase. A study on anterior cruciate ligament (ACL) injury demonstrated that mechanical stimulation increases the protein level of MMP-2 via TNF-α, IL-1β, and their combinations, thereby contributing to delayed healing of ACL injury (52). Another study demonstrated that under prolonged and high-intensity mechanical stretch stimulation, the TNF-α receptor p55 is gradually upregulated in rat myocardium, which may have a potential impact on myocardial compensatory hypertrophy (53). Additional studies have demonstrated that non-uniform shear stress can induces endothelial susceptibility to circulating TNF-α and adhesion of monocytic cells (54). Collectively, these studies demonstrate that TNF-α is indeed involved in the regulation of immune inflammation in various tissues under mechanical stimulation.

**Figure 10:**
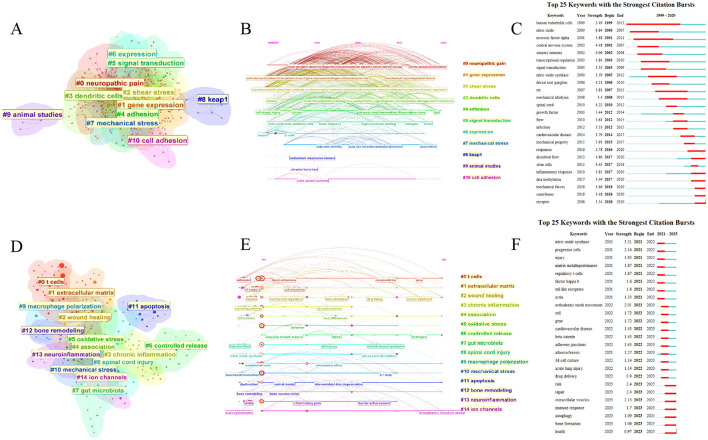
Independent keywords cluster analysis of the research exploration phase and the research explosion phase. **(A)** Keywords clusters in research exploration phase. **(B)** Timeline view of keywords clusters in research exploration phase. **(C)** Top 25 keywords with strongest citation bursts in research exploration phase. **(D)** Keywords clusters in research explosion phase. **(E)** Timeline view of keywords clusters in research explosion phase. **(F)** Top 25 keywords with strongest citation bursts in research explosion phase.

We re-conducted keyword clustering for the Research Exploration Phase and Research Explosion Phase of both WOSCC and Scopus data, and observed significant changes in the resulting clusters (Figure 10, Supplementary Files S4A–D, S3–S4). During the Research Exploration Phase, the major clusters in WOSCC were concentrated on keywords with broader descriptive scopes, such as “gene expression,” “shear stress,” “signal transduction,” “cell adhesion,” and “mechanical stress.” (Figures 10A–C) In contrast, the major clusters of the Research Explosion Phase featured more precise keywords—including “T cells,” “macrophage polarization,” “apoptosis,” and “ion channels”—which are more closely aligned with mechanistic research on immune-inflammatory regulation (Figures 10D–F). Concurrently, a shift in research directions was also observed. During the Research Explosion Phase, new major clusters emerged, such as “wound healing,” “gut microbiota,” and “bone remodeling.” This indicates that in recent years, research in the field of mechanical stress-mediated immune and inflammatory regulation has garnered attention from diverse disciplines. The influence of mechanical stress on wound repair by regulating immune-inflammatory responses has emerged as a direction of significant interest in recent years. For example, one study demonstrated that static mechanical stress with an amplitude of 15% promotes the polarization of macrophages toward the M2 phenotype, leading to increased release of anti-inflammatory cytokines and growth factors, as well as the activation of YAP and TAZ. This exhibits beneficial effects on enhancing wound angiogenesis and wound healing (55). Another study used an innovative skin stress-shielding hydrogel dressing to reduce the mechanical tension of wounds, thereby downregulating the expression of TGF-β1 and collagen I, and inhibiting the FAK/p-FAK pathway. This hydrogel helps reduce scar formation during wound healing (56). In the field of bone tissue repair, the latest research has found that fast stress-relaxing hydrogels exhibit a favorable osteogenic effect in a rat femoral defect model, with the simultaneous activation of TRPV4 and the Wnt/β-catenin pathway (57). Additionally, the role of mechanical stress in intestinal microbiota has gradually been explored. The latest research shows that in a Drosophila melanogaster model, groups of Drosophila melanogaster fed a indigestible diet containing methylcellulose exhibit increased microbiota-dependent ROS, activation of the IMD pathway, and an upregulation of IMD-dependent Tk expression. This suggests that diet-induced mechanical forces may have an impact on the immunity and metabolism of the digestive tract (58). Notably, clusters related to neurosurgery appeared in both the Research Exploration Phase and Research Explosion Phase. For instance, the Research Exploration Phase included clusters such as “neuropathic pain” and “dendritic cells,” while the Research Explosion Phase featured clusters like “neuroinflammation” and “spinal cord injury.” This consistency indicates that the role of mechanical stress in neuroinflammation regulation has consistently attracted attention. For example, this 2016 article systematically elaborates that mechanical stress can trigger the release of neuropeptides, which is one of the causes of neuroinflammation following traumatic brain injury (59).

The keyword clusters of the Research Explosion Phase reflect the primary research directions in recent years. This study will focus on emerging mechanistic research hotspots, among which T cells, macrophages, and mechanosensitive ion channels constitute key components of recent research focus. Under the “T cells” cluster, associations between T cells and keywords such as “cytoskeleton,” “focal adhesions,” and “stiffness” were identified (Supplementary File S4). For example, one study found that the cytoskeleton and Piezo1 ion channel respond to external shear stress, leading to the dephosphorylation and nuclear translocation of YAP1 and NFAT2. Another article provides a comprehensive account of the regulatory role of actin cytoskeleton dynamics in T cell development and function (60, 61). Immune cells exhibit high dynamism, and this latest study found that the efficient migration of activated primary Th1 T cells is mediated by small, dynamic focal adhesions. This confirms that integrin-mediated focal adhesions play a critical role in T cell motility (62). Notably, although ECM stiffness has long been a classic research direction regarding the regulation of immune inflammation by mechanical stress, a novel biomechanical checkpoint, Osr2, was still identified in 2024. Experiments have demonstrated that Osr2 integrates biomechanical signaling and promotes the terminal exhaustion of tumor-reactive CD8+ T cells (63). As one of the key means by which mechanical stress regulates immune-inflammatory responses, matrix stiffness has also been a major focus of researchers in recent years—and this may also be related to the growing enthusiasm for research on mechanical stress in wound repair processes in recent years. Matrix stiffness not only affects T cell regulation but also exerts influences on various immune and inflammatory cells. Matrix viscoelasticity can coordinate osteogenesis by regulating macrophage metabolism through the VASP/HIF1α signaling transduction pathway (64). Microglia are resident immune cells in the brain. New research has found that matrix stiffness can regulate the secretion of interleukin-10 (IL-10) in human microglia through YAP-mediated mechanotransduction (65). Under the “macrophage polarization” cluster (Supplementary File S4), a close correlation was observed between macrophage polarization and YAP/TAZ—a transcriptional complex in the Hippo pathway. YAP/TAZ are classical mechanotransduction factors, which has been validated by multiple studies (66–68). The latest research has found that YAP/TAZ is activated in lung macrophages of both patients with pulmonary fibrosis and bleomycin-induced pulmonary fibrosis mice. Targeting abnormal YAP/TAZ activity to regulate inflammatory responses may serve as a promising strategy for the prevention and treatment of pulmonary fibrosis (69). For another example, as mentioned earlier, mechanical stress promotes the polarization of macrophages toward the M2 phenotype via YAP/TAZ, thereby facilitating the wound healing process (55). Under the “ion channels” cluster, strong associations between ion channels and keywords like “macrophages” and “actin cytoskeleton” were observed (Supplementary File S4). For example, in dextran sulfate sodium (DSS)-induced acute and chronic inflammatory bowel disease (IBD), the activation of the Piezo1 mechanosensitive ion channel in macrophages enhances the secretion of proinflammatory cytokines via the NLRP3/ NF-κB pathways (70). The actin cytoskeleton serves as a key structure for cell migration and morphological changes, and it modulates the mechanical properties of cells. In chondrocytes, abnormal mechanical stimulation activates the Piezo1 ion channel, leading to an excessive accumulation of intracellular Ca^2^^+^ in chondrocytes, subsequent thinning of the F-actin cytoskeleton, and potentially culminating in temporomandibular joint arthritis (71). Additionally, another type of cell has drawn our attention—natural killer (NK) cells. In the Research Explosion Phase of Scopus data, the keyword “natural killer cell” showed a short-term surge between 2023 and 2025, which may indicate that NK cells will become the next key focus cell type following macrophages (Supplementary Figure S4D). For example, recent studies have found that fluid shear stress can activate the NKG2D receptor, thereby inducing the activation and degranulation of NK cells (72).

Returning focus to the citation burst analysis of references, these burst-cited references likely represent the knowledge system foundation of the current Research Explosion Phase. The five references that have experienced citation bursts since 2021 cover significant overlapping content areas: two focus on Piezo1 research, two are related to macrophages, one discusses the effects of ECM viscoelasticity, and one addresses the impact of mechanical stress on stem cells (Figure 8B). Among two high-impact studies on Piezo1, one found that mice deficient in the Piezo1 protein in innate immune cells exhibited significantly reduced lung inflammation. The other demonstrated that Piezo1-deficient macrophages showed attenuated inflammatory responses and enhanced wound healing responses (34, 73). These studies collectively indicate that Piezo1 serves as a critical target for regulating immune-inflammatory responses and has been a key focus of research in recent years. The influence of extracellular matrix stiffness on immune cells has also emerged as a highly popular research direction, driven by the recent development of novel biomaterials and their applications across various fields. Changes in the mechanical properties of materials in contact with cells can profoundly affect cellular behavior. Notably, modulating matrix stiffness can also alter macrophage function via Piezo1 (34).

Given that research on mechanoimmunology has entered an intense phase, some of the key targets mentioned earlier in this article are also in translational research. For instance, Piezo1 inhibitors have been validated in liver cancer and glioblastoma models for their ability to enhance anti-tumor immune effects. Meanwhile, paclitaxel—a microtubule-targeting agent commonly used in cancer chemotherapy—has recently been found to enhance Piezo2 signaling responses, thereby participating in the regulation of the tumor microenvironment (63, 74, 75). Additionally, antagonists and agonists of the mechanosensitive receptor TRPV4 are also being used in trials for tumor intervention therapy (76). Notably, YAP/TAZ inhibitors are among the most actively pursued targets in current clinical drug development. However, they also face a critical challenge during development: these inhibitors may simultaneously inhibit other normal cellular activities. This has led to unavoidable side effects such as nephrotoxicity and lack of specificity (77). It is not difficult to observe that current research and translation in the field of mechanoimmunology primarily focus on various cancer-related directions. By regulating factors such as ECM stiffness and the cytoskeletal structure of tumor cells themselves, it is possible to modulate the tumor microenvironment from a mechanical force perspective and inhibit tumor cell proliferation and migration. Currently, several reliable target inhibitors have entered clinical research phases, for example, the integrin inhibitor Cilengitide has demonstrated significant efficacy and acceptable toxicity in combination therapy with chemotherapy for small-cell lung cancer (78). This interdisciplinary field of biomechanics and immunology holds great promise and has gradually been applied in current research on tumor intervention therapy.

## 5 Limitation

The WOSCC and Scopus databases differ slightly in their literature inclusion criteria. WOSCC has more stringent literature screening standards, can trace back to older literature, and most importantly, offers superior software support for citation network analysis. In contrast, Scopus has less stringent literature inclusion criteria, it covers a broader range of literature types, including more timely content such as preprints. However, software compatibility with Scopus for citation network analysis is slightly lacking. Owing to the regular updates and removals of articles in the WOSCC and Scopus database, discrepancies in the number of retrieved articles may occur when conducting searches on different dates—even under the same search strategy. Meanwhile, due to the low compatibility of bibliometric software with multilingual literature analysis, this study did not include non-English literature, which accounts for a very small proportion of the total. However, these quantitative discrepancies fall within an acceptable range and do not affect the derivation of the conclusions in this study. Meanwhile, due to the limitations of text recognition in various bibliometric software tools, systematic biases such as inaccurate recognition of author names in certain countries may arise. In this study, statistical analyses were solely based on the calculation results of bibliometric software, with no manual intervention to modify outcomes—this approach avoids introducing secondary data bias. Additionally, compared to other sections of the study (where a top 10 ranking was routinely used for statistical analysis), the scope of author-related statistics was expanded from the conventional top 10 to top 20. This adjustment was implemented to mitigate potential systematic biases in author-related analyses. A key limitation of bibliometrics itself is that older publications are more likely to be assigned greater significance in analyses, as they have had more opportunities to be cited. Additionally, among recent studies published in recent years, only highly disruptive or innovative ones tend to stand out in bibliometric assessments, other studies may be overlooked due to their low citation counts. However, this does not negate their contributions to the field or their inherent innovativeness. In summary, bibliometric analysis serves as a systematic retrospective review of established authoritative research and cannot replace prospective research.

## 6 Conclusion

In summary, research in the field of mechanoimmunology is currently in the Research Explosion Phase, with 2021 marking the onset of a new era for studies in this domain. Cardiovascular disease represented the early research direction in this field; however, the current research focus has expanded rapidly into diverse areas, including biomaterials, wound repair, bone tissue repair, and gut microbiota research. This shift in disciplinary research directions is one of the key drivers behind the current Research Explosion Phase. Another factor contributing to the field's burgeoning growth is the rapid rise of China as a research force. The US and China stand as the two global supercenters for research in this field: the US dominates current global collaborations, while China is gradually expanding its influence. T cells and macrophages are the primary cell types of focus in current research within the field. Additionally, the mechanosensitive ion channel protein Piezo1 and the YAP/TAZ transcriptional complex in the Hippo pathway have emerged as prominent targets in recent studies. Furthermore, Osr2, a key novel biomechanical checkpoint discovered in 2024, is likely to be a highly promising new research target in the coming years. With the rapid advancement of 3D printing technology, microfluidic technology, and microscale mechanical force detection technology, our research group posits that regulating mechanical stress to control the occurrence and progression of immune inflammation represents a highly promising direction for both academic research and industrial applications.

## Data Availability

The original contributions presented in the study are included in the article/Supplementary material, further inquiries can be directed to the corresponding authors.

## Supplementary material

Supplementary Figure S1 Top 10 countries by publication volume in Scopus.

Supplementary Figure S2 Keywords co-occurrence in Scopus.

Supplementary Figure S3 Keywords cluster and citation burst analysis in Scopus. **(A)** Keywords clusters. **(B)** Top 25 keywords with the strongest citation bursts.

Supplementary Figure S4 Independent keywords cluster and citation burst analysis of the research exploration phase and the research explosion phase in Scopus. **(A)** Keywords clusters in research exploration phase. **(B)** Top 25 keywords with strongest citation bursts in research exploration phase. **(C)** Keywords clusters in research explosion phase. **(D)** Top 25 keywords with strongest citation bursts in research explosion phase.

Supplementary Table S1 Annual production volume in Scopus.

Supplementary Table S2 The top 10 journals in Scopus.

Supplementary Table S3 The top 20 authors in Scopus.

Supplementary Table S4 The top 20 authors in WOSCC.

Supplementary Table S5 The top 10 affiliations in Scopus.

Supplementary Table S6 The multi-country publication rate among the top 10 publication-output countries.

Supplementary Table S7 The top 10 most global cited article in the research field of mechanoimmunology.

Supplementary File S1 Each cluster of all time.

Supplementary File S2 Annual publication categories.

Supplementary File S3 Each cluster in exploration phase.

Supplementary File S4 Each cluster in explosion phase.

Supplementary File S5 WOSCC dataset.

Supplementary File S6 Scopus dataset.
