# Supplementary figures and images for: Mechanical stress-mediated immune and inflammatory regulation: a bibliometric and visualization analysis of mechanoimmunology based on two databases
Source: Front Med (Lausanne). 2025 Nov 6;12:1698177. doi: 10.3389/fmed.2025.1698177 (PMC12631210; doi:10.3389/fmed.2025.1698177)

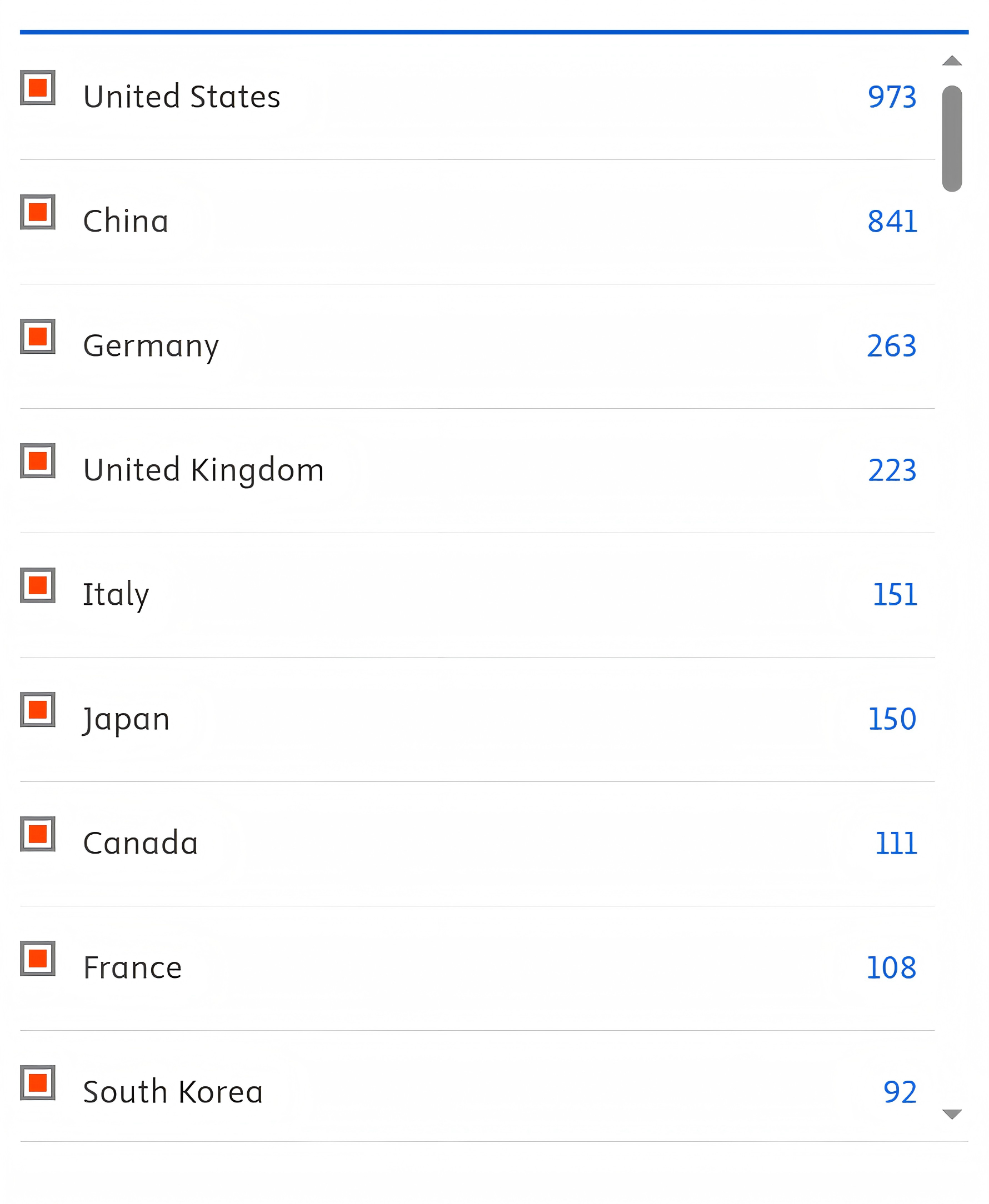

Supplement: Supplementary Figure S1 — Top 10 countries by publication volume in Scopus. [file Image_1.tif]

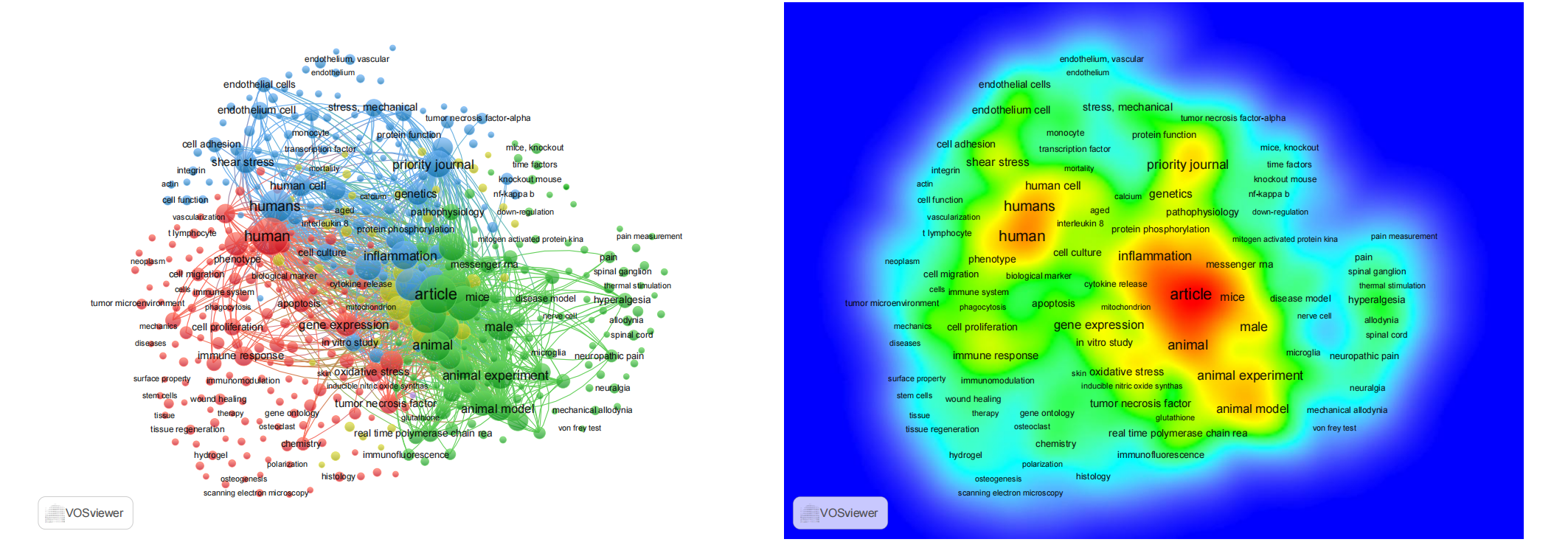

Supplement: Supplementary Figure S2 — Keywords co-occurrence in Scopus. [file Image_2.tif]

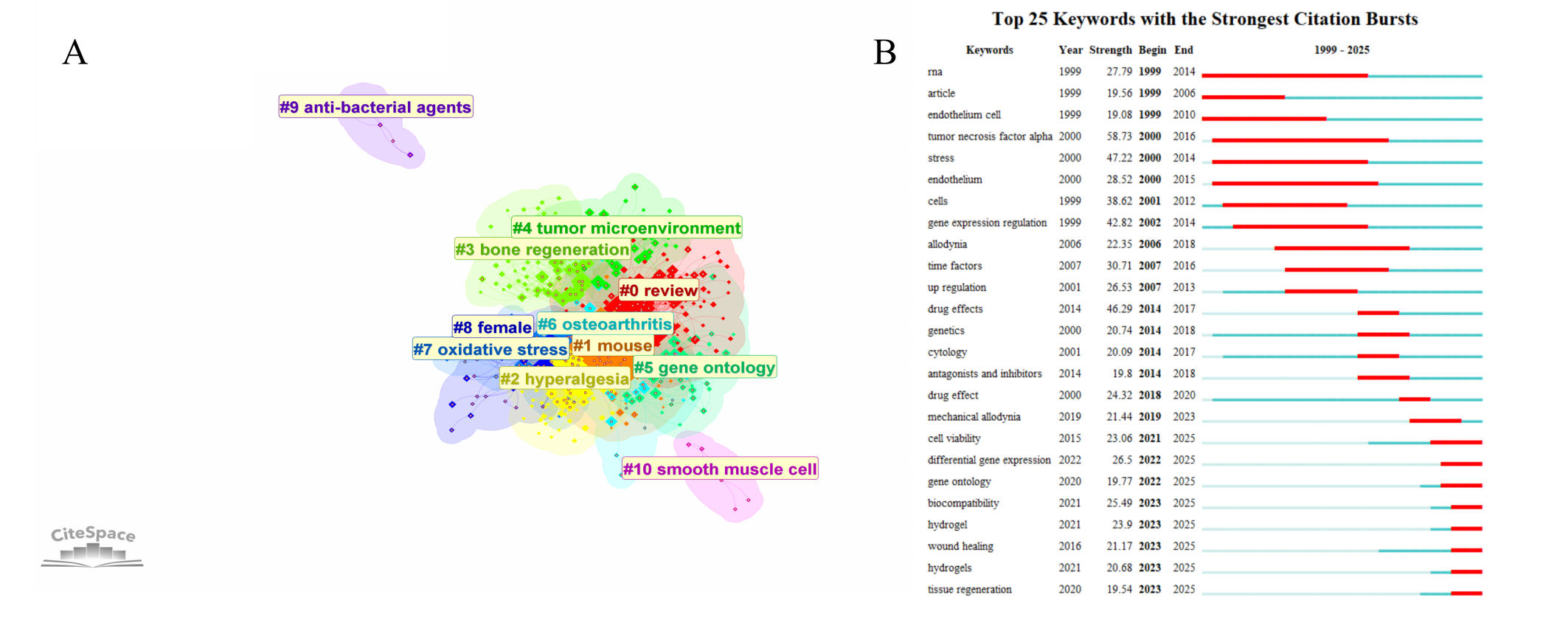

Supplement: Supplementary Figure S3 — Keywords cluster and citation burst analysis in Scopus. (A) Keywords clusters. (B) Top 25 keywords with the strongest citation bursts. [file Image_3.tif]

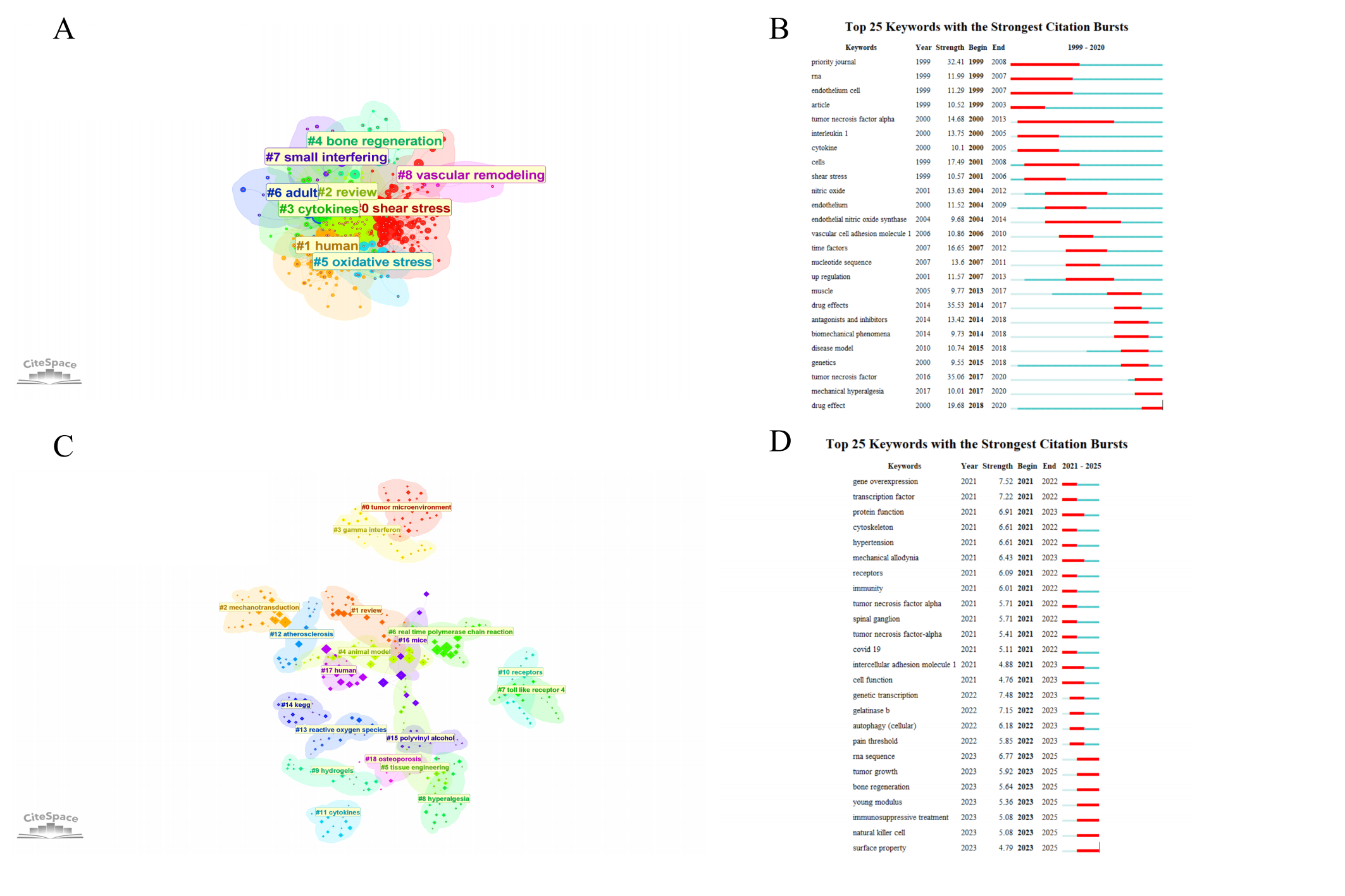

Supplement: Supplementary Figure S4 — Independent keywords cluster and citation burst analysis of the research exploration phase and the research explosion phase in Scopus. (A) Keywords clusters in research exploration phase. (B) Top 25 keywords with strongest citation bursts in research exploration phase. (C) Keywords clusters in research explosion phase. (D) Top 25 keywords with strongest citation bursts in research explosion phase. [file Image_4.tif]

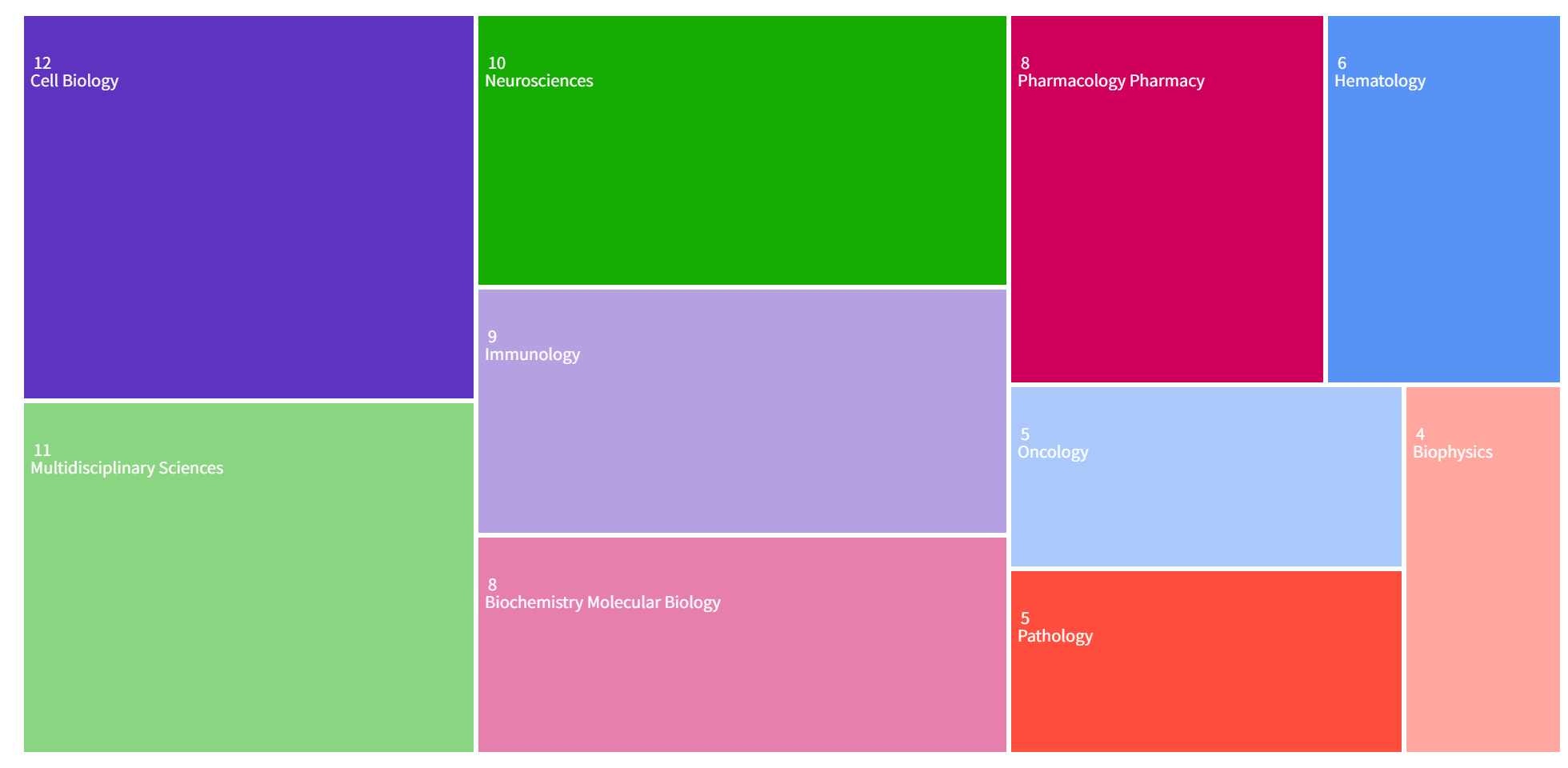

Supplement: Supplementary File S2 — Annual publication categories. [file Data_Sheet_2.zip › annual pub categories/2016visualization.jpg]

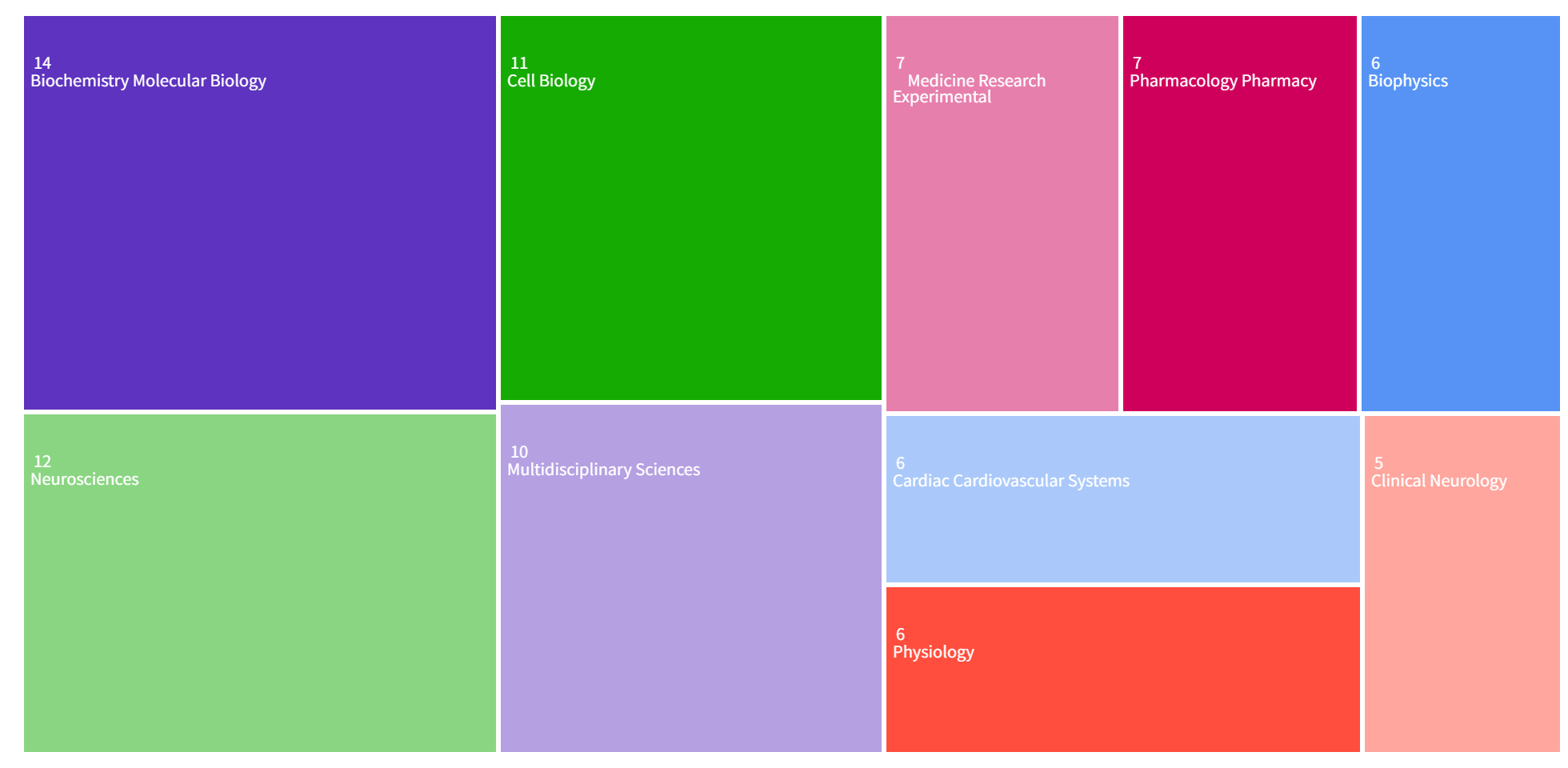

Supplement: Supplementary File S2 — Annual publication categories. [file Data_Sheet_2.zip › annual pub categories/2017visualization.jpg]

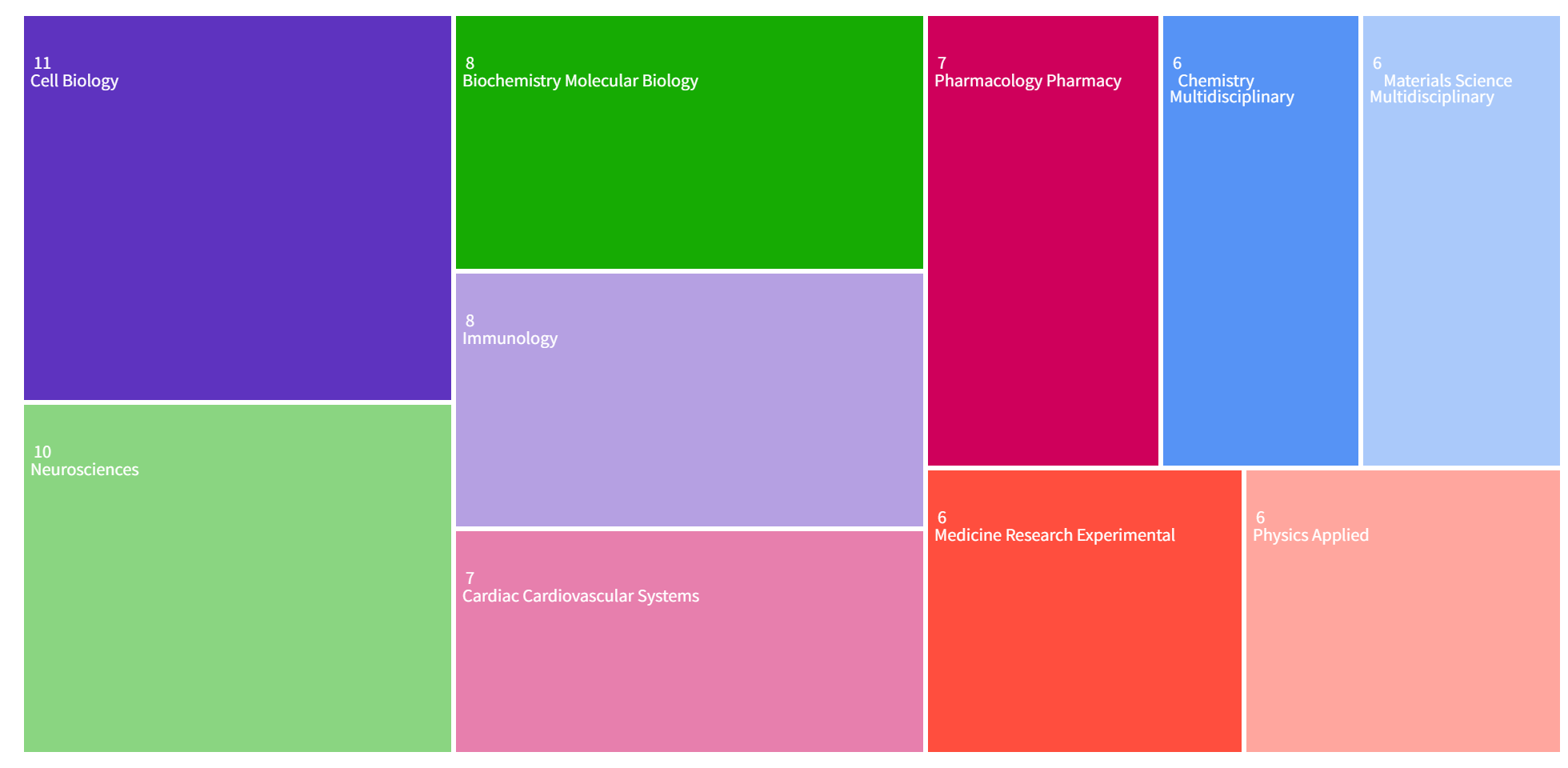

Supplement: Supplementary File S2 — Annual publication categories. [file Data_Sheet_2.zip › annual pub categories/2018visualization.jpg]

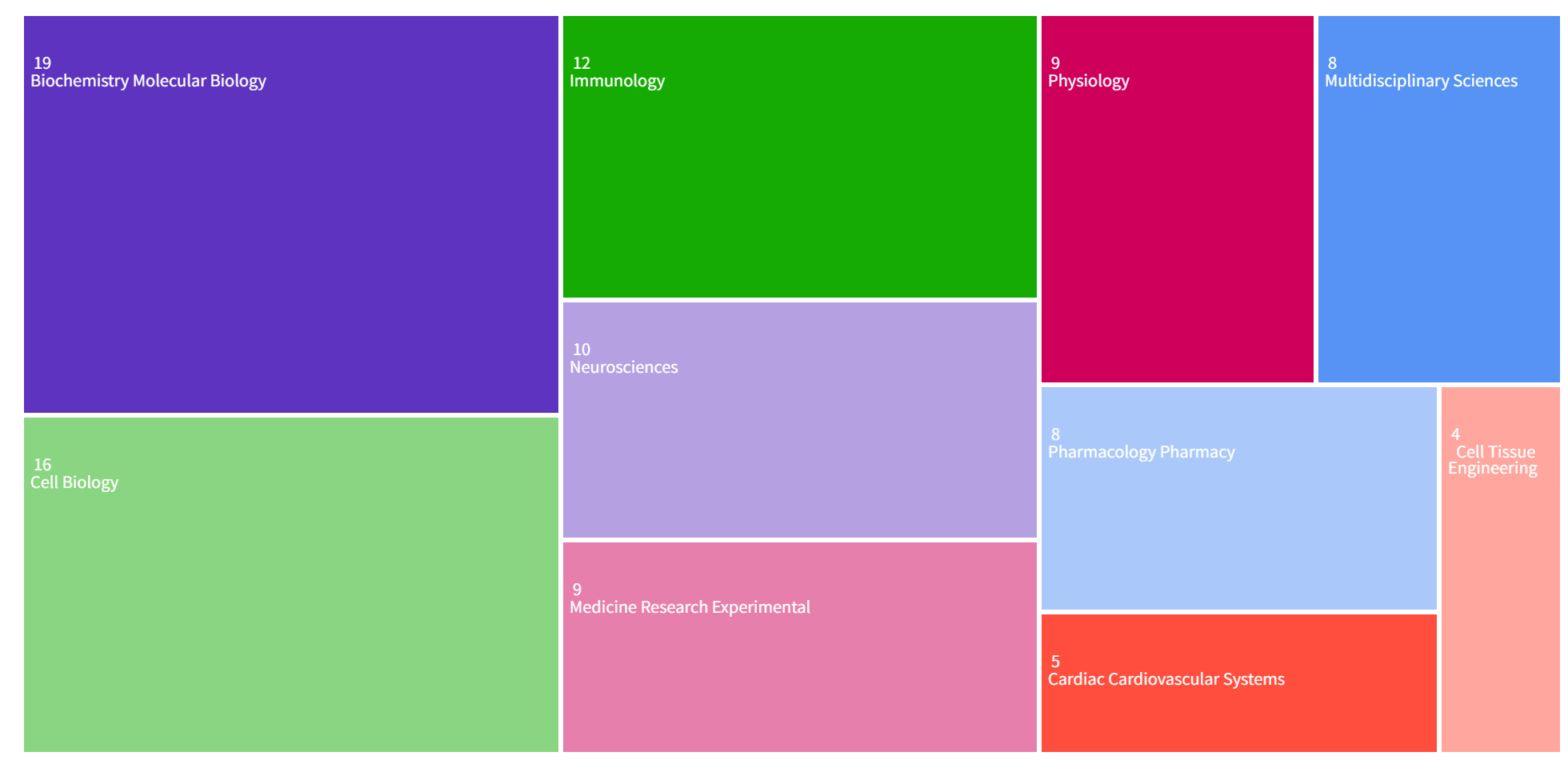

Supplement: Supplementary File S2 — Annual publication categories. [file Data_Sheet_2.zip › annual pub categories/2019visualization.jpg]

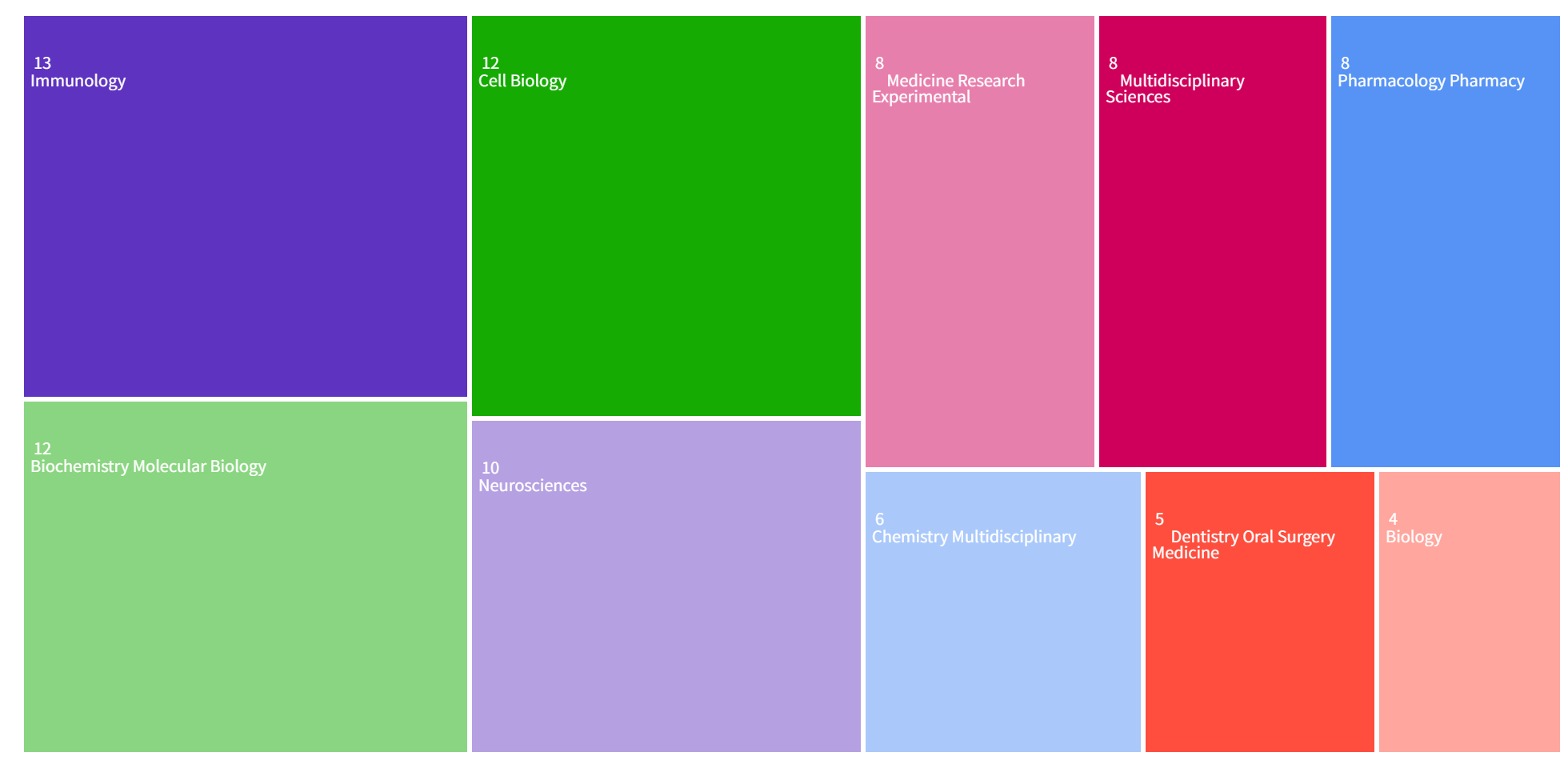

Supplement: Supplementary File S2 — Annual publication categories. [file Data_Sheet_2.zip › annual pub categories/2020visualization.jpg]

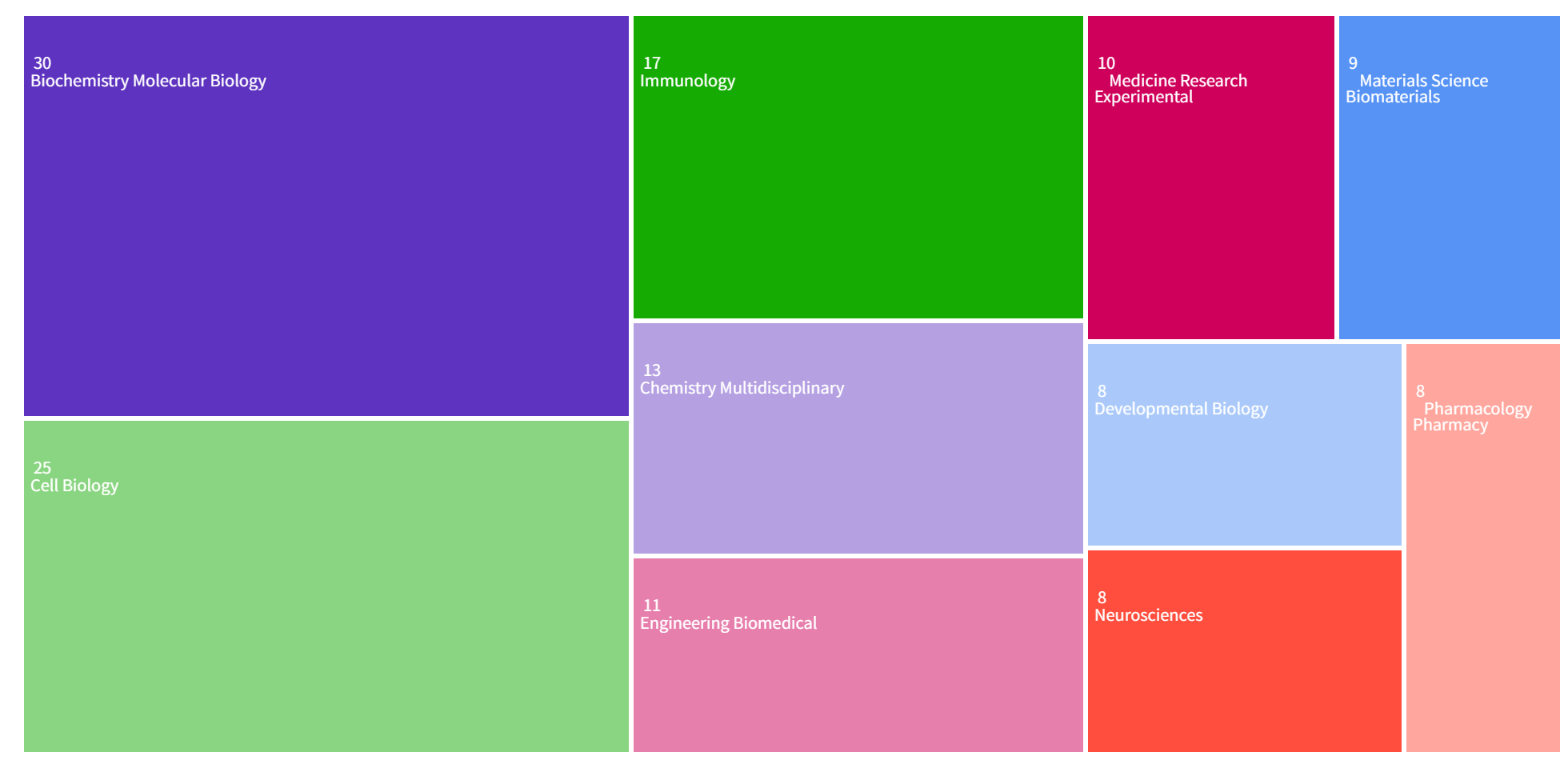

Supplement: Supplementary File S2 — Annual publication categories. [file Data_Sheet_2.zip › annual pub categories/2021visualization.jpg]

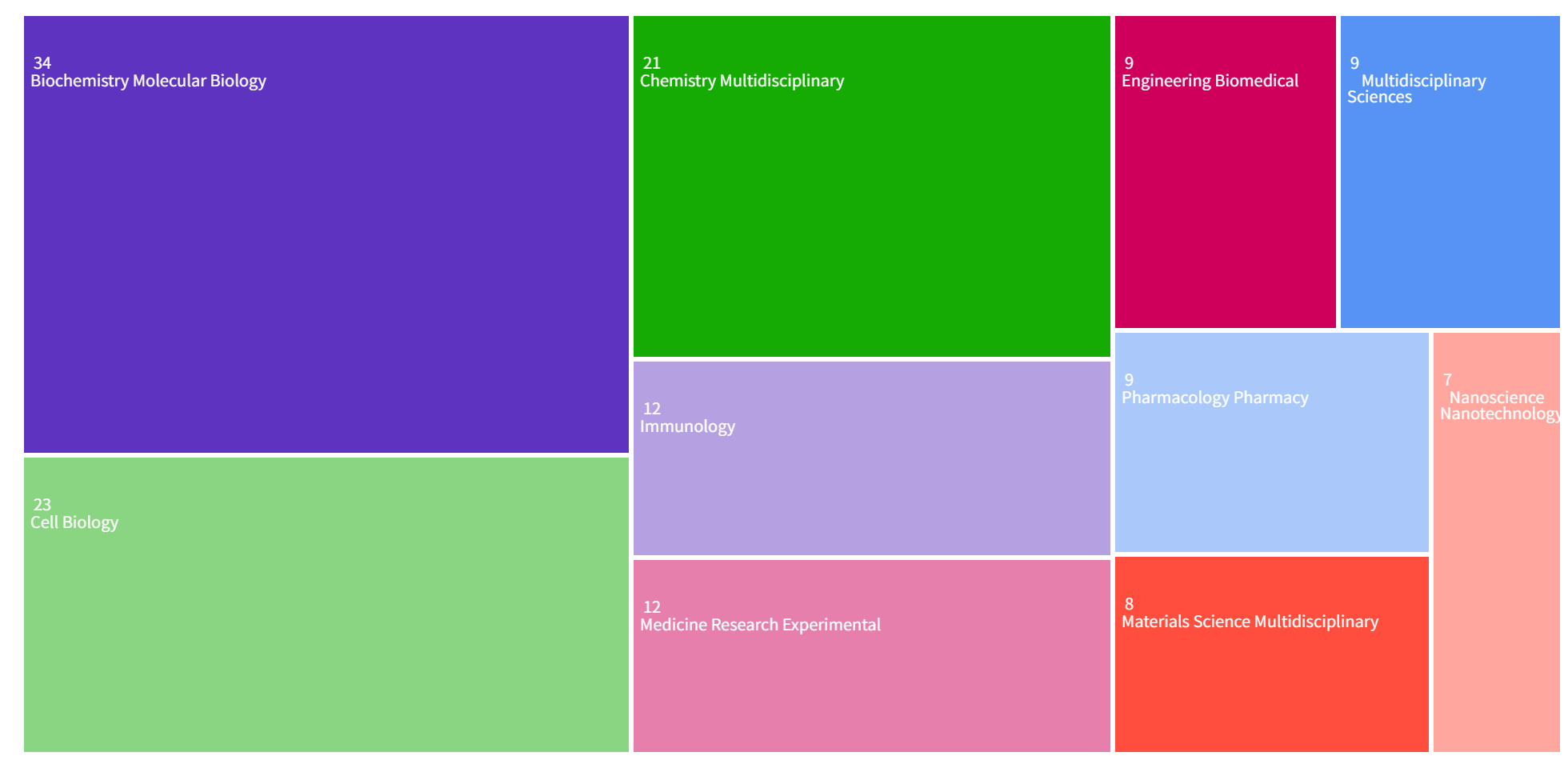

Supplement: Supplementary File S2 — Annual publication categories. [file Data_Sheet_2.zip › annual pub categories/2022visualization.jpg]

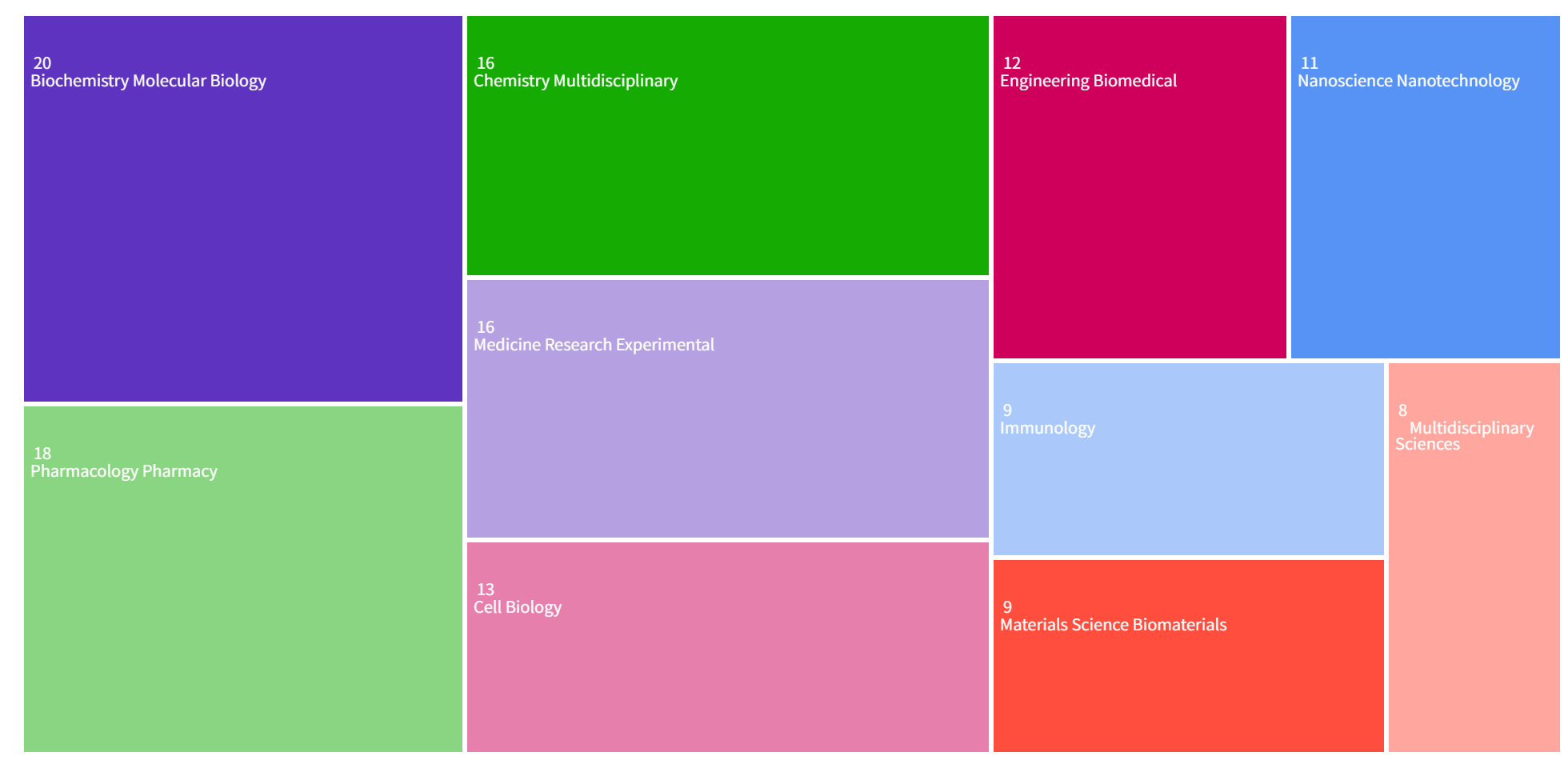

Supplement: Supplementary File S2 — Annual publication categories. [file Data_Sheet_2.zip › annual pub categories/2023visualization.jpg]

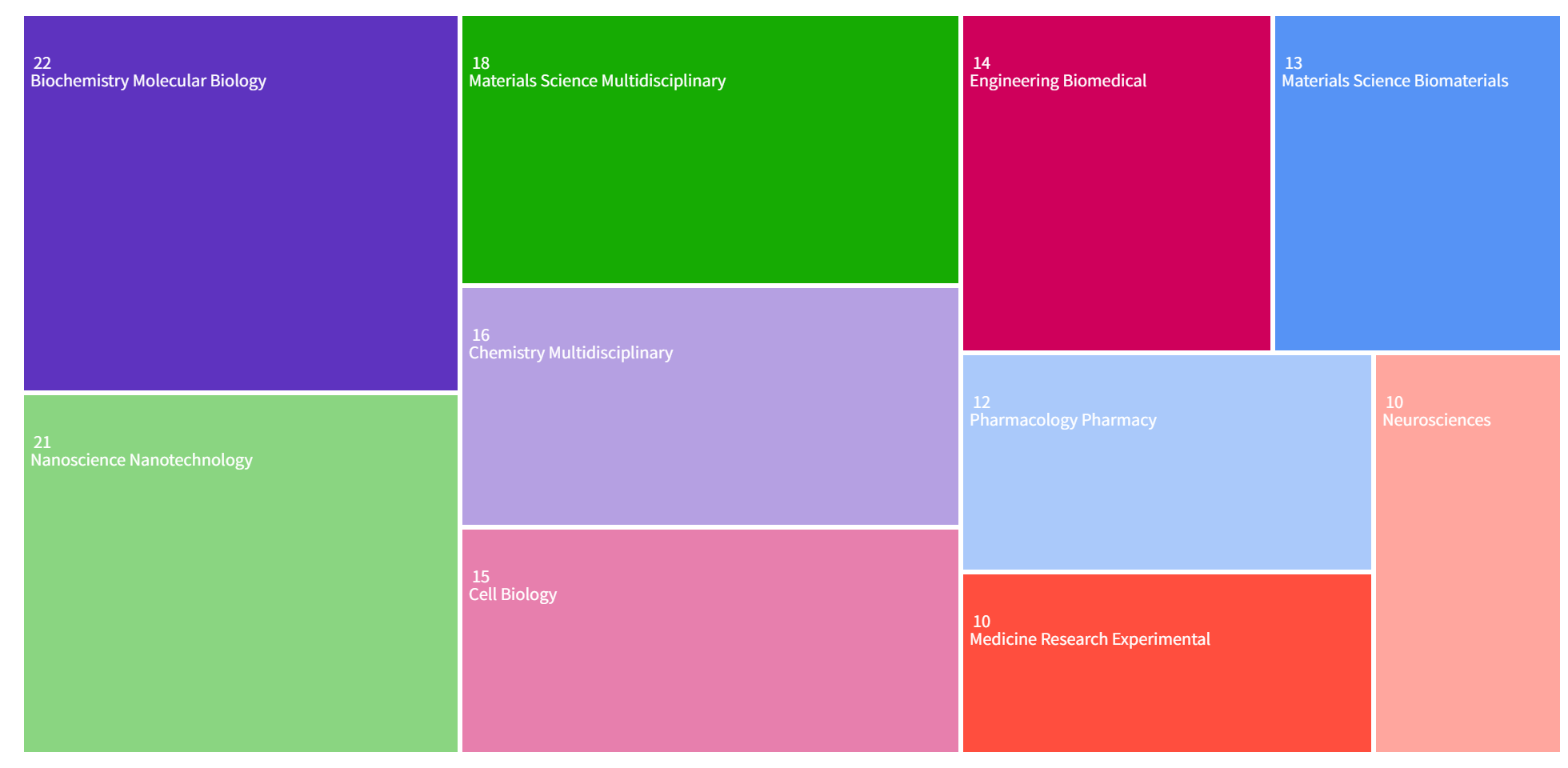

Supplement: Supplementary File S2 — Annual publication categories. [file Data_Sheet_2.zip › annual pub categories/2024visualization.jpg]

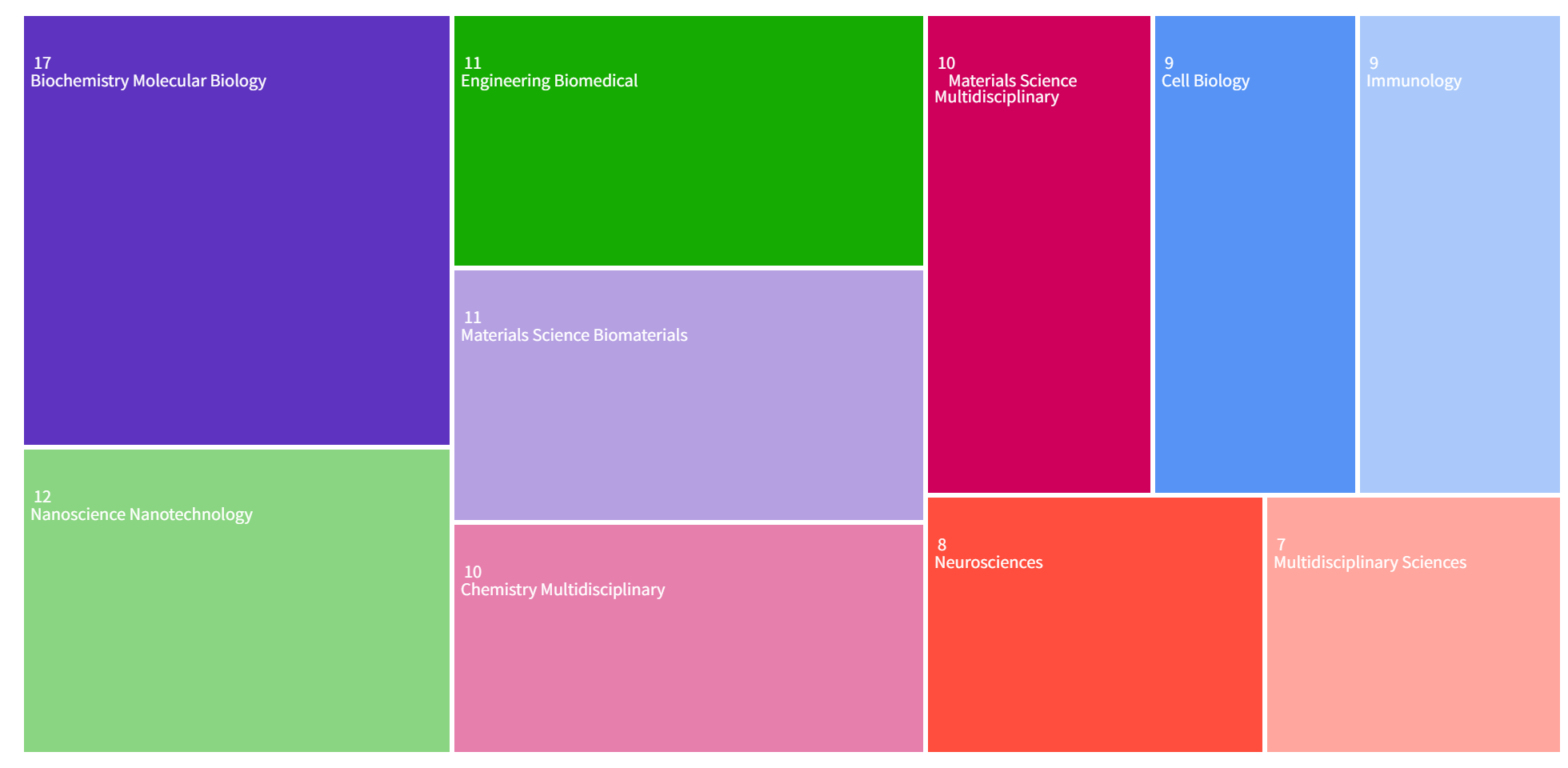

Supplement: Supplementary File S2 — Annual publication categories. [file Data_Sheet_2.zip › annual pub categories/2025visualization.jpg]
