# Supplementary material for: Mechanical stress-mediated immune and inflammatory regulation: a bibliometric and visualization analysis of mechanoimmunology based on two databases
Source: Front Med (Lausanne). 2025 Nov 6;12:1698177. doi: 10.3389/fmed.2025.1698177 (PMC12631210; doi:10.3389/fmed.2025.1698177)
Supplement: Supplementary File S1 — Each cluster of all time. [file Data_Sheet_1.zip › each cluster of all time/cluster0.pdf]

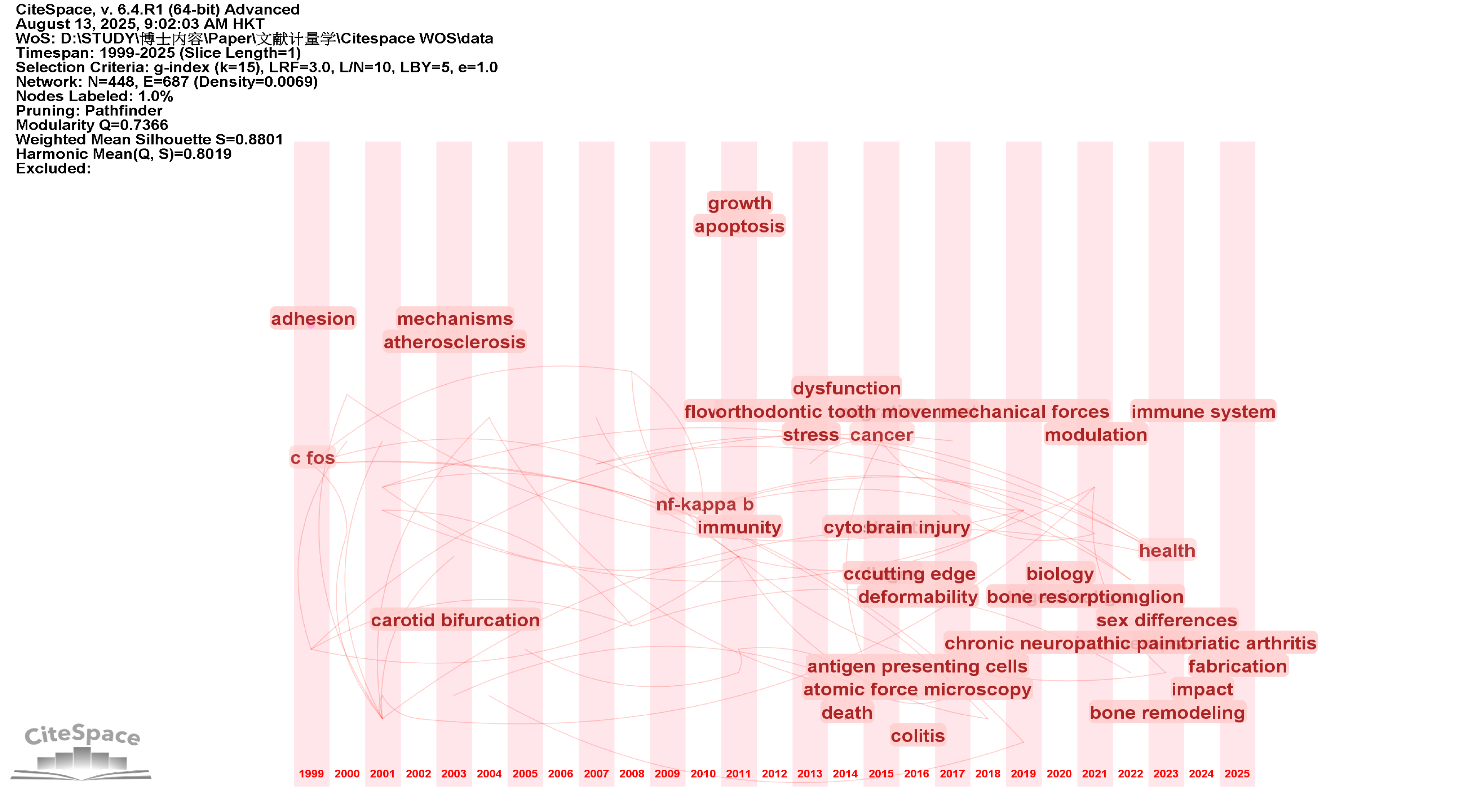
CiteSpace, v. 6.4.R1 (64-bit) Advanced
August 13, 2025, 9:02:03 AM HKT
WoS: D:\STUDY\博士内容\Paper\文献计量学\Citespace WOS\data
Timespan: 1999-2025 (Slice Length=1)
Selection Criteria: g-index (k=15), LRF=3.0, L/N=10, LBY=5, e=1.0
Network: N=448, E=687 (Density=0.0069)
Nodes Labeled: 1.0%
Pruning: Pathfinder
Modularity Q=0.7366
Weighted Mean Silhouette S=0.8801
Harmonic Mean(Q, S)=0.8019
Excluded:
growth
apoptosis
adhesion
mechanisms
atherosclerosis
dysfunction
orthodontic tooth moven
mechanical forces
immune system
stress
cancer
modulation
c fos
nf-kappa b
immunity
brain injury
health
cutting edge
biology
deformability
bone resorption
sex differences
carotid bifurcation
chronic neuropathic pain
psoriatic arthritis
antigen presenting cells
fabrication
atomic force microscopy
impact
death
bone remodeling
colitis
CiteSpace
1999
2000
2001
2002
2003
2004
2005
2006
2007
2008
2009
2010
2011
2012
2013
2014
2015
2016
2017
2018
2019
2020
2021
2022
2023
2024
2025
