# Supplementary material for: Mechanical stress-mediated immune and inflammatory regulation: a bibliometric and visualization analysis of mechanoimmunology based on two databases
Source: Front Med (Lausanne). 2025 Nov 6;12:1698177. doi: 10.3389/fmed.2025.1698177 (PMC12631210; doi:10.3389/fmed.2025.1698177)
Supplement: Supplementary File S1 — Each cluster of all time. [file Data_Sheet_1.zip › each cluster of all time/cluster10.pdf]

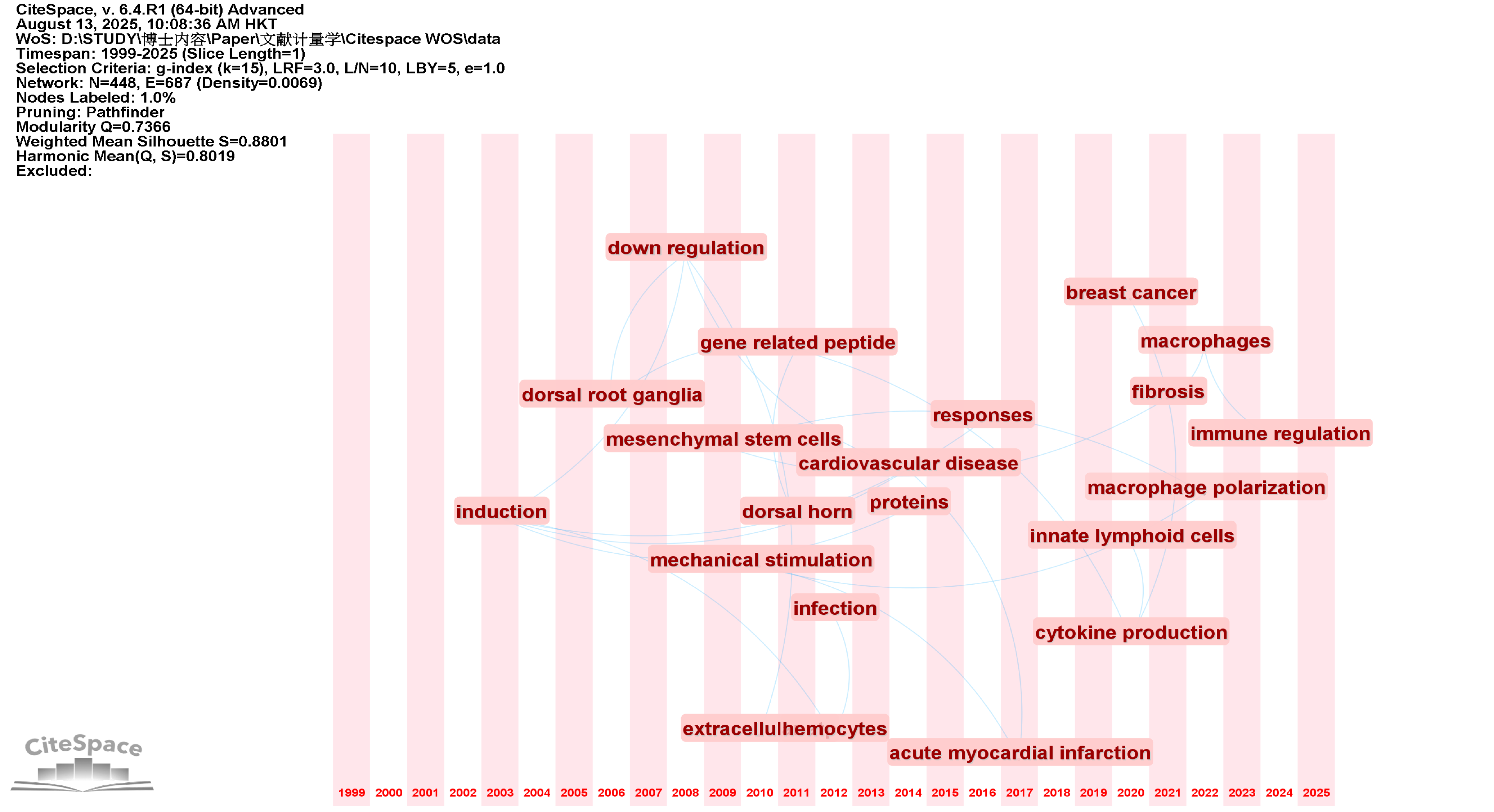
CiteSpace, v. 6.4.R1 (64-bit) Advanced
August 13, 2025, 10:08:36 AM HKT
WoS: D:\STUDY\博士内容\Paper\文献计量学\Citespace WOS\data
Timespan: 1999-2025 (Slice Length=1)
Selection Criteria: g-index (k=15), LRF=3.0, L/N=10, LBY=5, e=1.0
Network: N=448, E=687 (Density=0.0069)
Nodes Labeled: 1.0%
Pruning: Pathfinder
Modularity Q=0.7366
Weighted Mean Silhouette S=0.8801
Harmonic Mean(Q, S)=0.8019
Excluded:
down regulation
breast cancer
gene related peptide
macrophages
dorsal root ganglia
fibrosis
responses
immune regulation
mesenchymal stem cells
cardiovascular disease
macrophage polarization
induction
dorsal horn
proteins
innate lymphoid cells
mechanical stimulation
infection
cytokine production
extracellulhemocytes
acute myocardial infarction
CiteSpace
1999
2000
2001
2002
2003
2004
2005
2006
2007
2008
2009
2010
2011
2012
2013
2014
2015
2016
2017
2018
2019
2020
2021
2022
2023
2024
2025
