# Supplementary material for: Mechanical stress-mediated immune and inflammatory regulation: a bibliometric and visualization analysis of mechanoimmunology based on two databases
Source: Front Med (Lausanne). 2025 Nov 6;12:1698177. doi: 10.3389/fmed.2025.1698177 (PMC12631210; doi:10.3389/fmed.2025.1698177)
Supplement: Supplementary File S1 — Each cluster of all time. [file Data_Sheet_1.zip › each cluster of all time/cluster11.pdf]

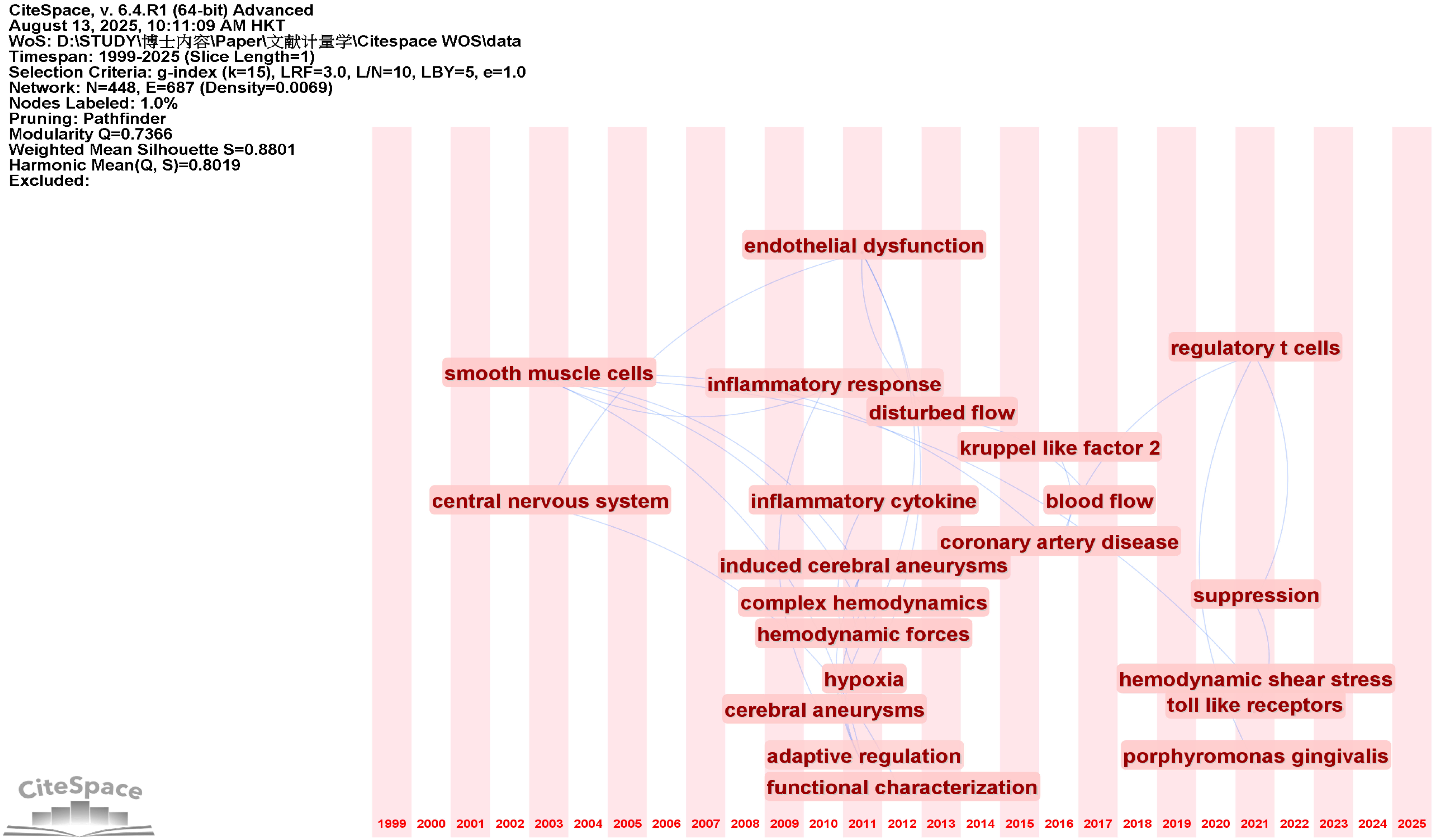
CiteSpace, v. 6.4.R1 (64-bit) Advanced
August 13, 2025, 10:11:09 AM HKT
WoS: D:\STUDY\博士内容\Paper\文献计量学\Citespace WOS\data
Timespan: 1999-2025 (Slice Length=1)
Selection Criteria: g-index (k=15), LRF=3.0, L/N=10, LBY=5, e=1.0
Network: N=448, E=687 (Density=0.0069)
Nodes Labeled: 1.0%
Pruning: Pathfinder
Modularity Q=0.7366
Weighted Mean Silhouette S=0.8801
Harmonic Mean(Q, S)=0.8019
Excluded:
endothelial dysfunction
regulatory t cells
smooth muscle cells
inflammatory response
disturbed flow
kruppel like factor 2
central nervous system
inflammatory cytokine
blood flow
coronary artery disease
induced cerebral aneurysms
suppression
complex hemodynamics
hemodynamic forces
hypoxia
hemodynamic shear stress
toll like receptors
cerebral aneurysms
adaptive regulation
porphyromonas gingivalis
functional characterization
CiteSpace
1999
2000
2001
2002
2003
2004
2005
2006
2007
2008
2009
2010
2011
2012
2013
2014
2015
2016
2017
2018
2019
2020
2021
2022
2023
2024
2025
