# Supplementary material for: Mechanical stress-mediated immune and inflammatory regulation: a bibliometric and visualization analysis of mechanoimmunology based on two databases
Source: Front Med (Lausanne). 2025 Nov 6;12:1698177. doi: 10.3389/fmed.2025.1698177 (PMC12631210; doi:10.3389/fmed.2025.1698177)
Supplement: Supplementary File S1 — Each cluster of all time. [file Data_Sheet_1.zip › each cluster of all time/cluster3.pdf]

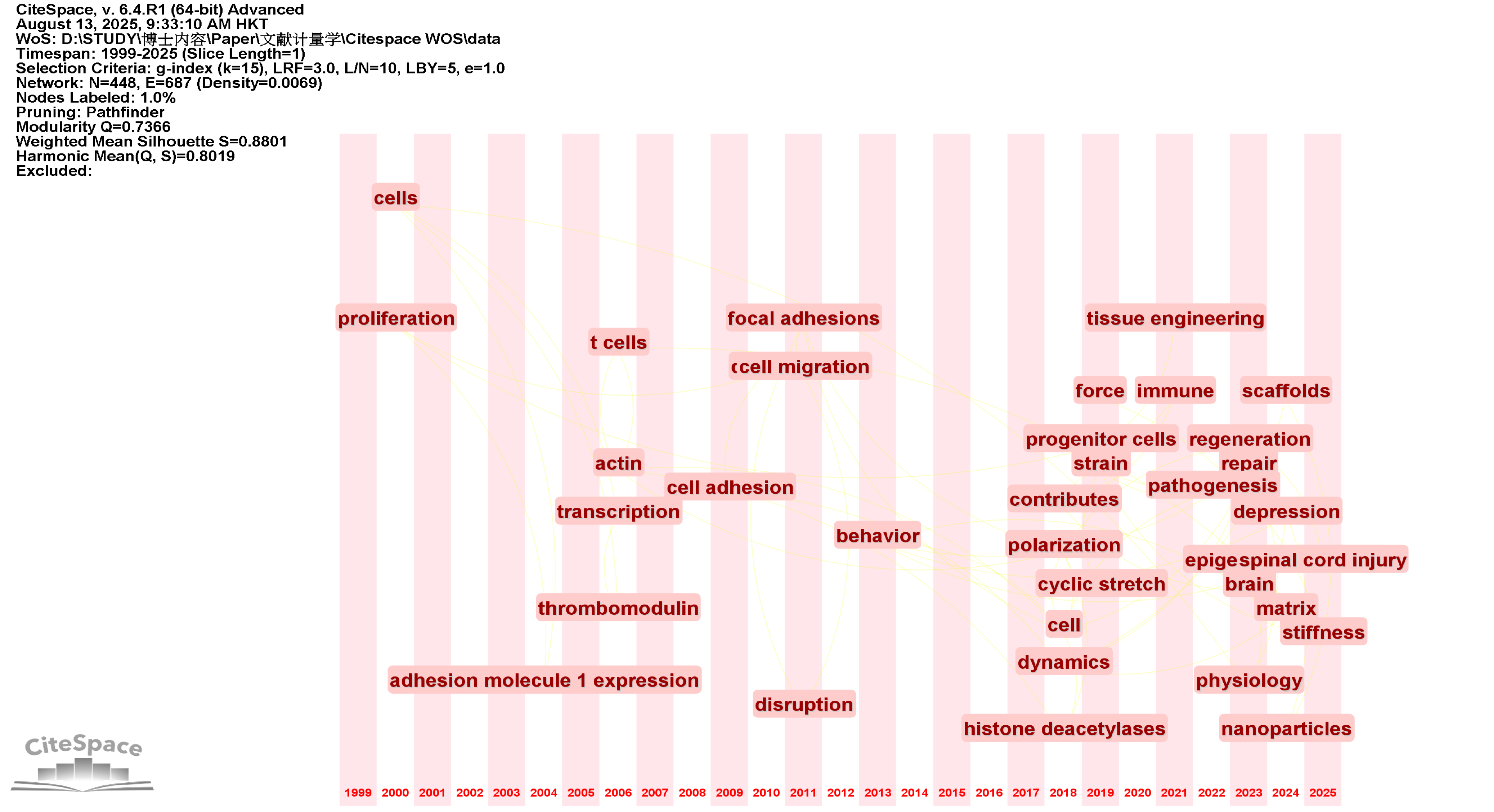
CiteSpace, v. 6.4.R1 (64-bit) Advanced
August 13, 2025, 9:33:10 AM HKT
WoS: D:\STUDY\博士内容\Paper\文献计量学\Citespace WOS\data
Timespan: 1999-2025 (Slice Length=1)
Selection Criteria: g-index (k=15), LRF=3.0, L/N=10, LBY=5, e=1.0
Network: N=448, E=687 (Density=0.0069)
Nodes Labeled: 1.0%
Pruning: Pathfinder
Modularity Q=0.7366
Weighted Mean Silhouette S=0.8801
Harmonic Mean(Q, S)=0.8019
Excluded:
cells
proliferation
t cells
focal adhesions
cell migration
tissue engineering
force
immune
scaffolds
progenitor cells
regeneration
strain
repair
actin
cell adhesion
pathogenesis
contributes
transcription
depression
behavior
polarization
epigespinal cord injury
cyclic stretch
brain
thrombomodulin
matrix
cell
stiffness
dynamics
adhesion molecule 1 expression
physiology
disruption
histone deacetylases
nanoparticles
CiteSpace
1999
2000
2001
2002
2003
2004
2005
2006
2007
2008
2009
2010
2011
2012
2013
2014
2015
2016
2017
2018
2019
2020
2021
2022
2023
2024
2025
