# Supplementary material for: Mechanical stress-mediated immune and inflammatory regulation: a bibliometric and visualization analysis of mechanoimmunology based on two databases
Source: Front Med (Lausanne). 2025 Nov 6;12:1698177. doi: 10.3389/fmed.2025.1698177 (PMC12631210; doi:10.3389/fmed.2025.1698177)
Supplement: Supplementary File S1 — Each cluster of all time. [file Data_Sheet_1.zip › each cluster of all time/cluster4.pdf]

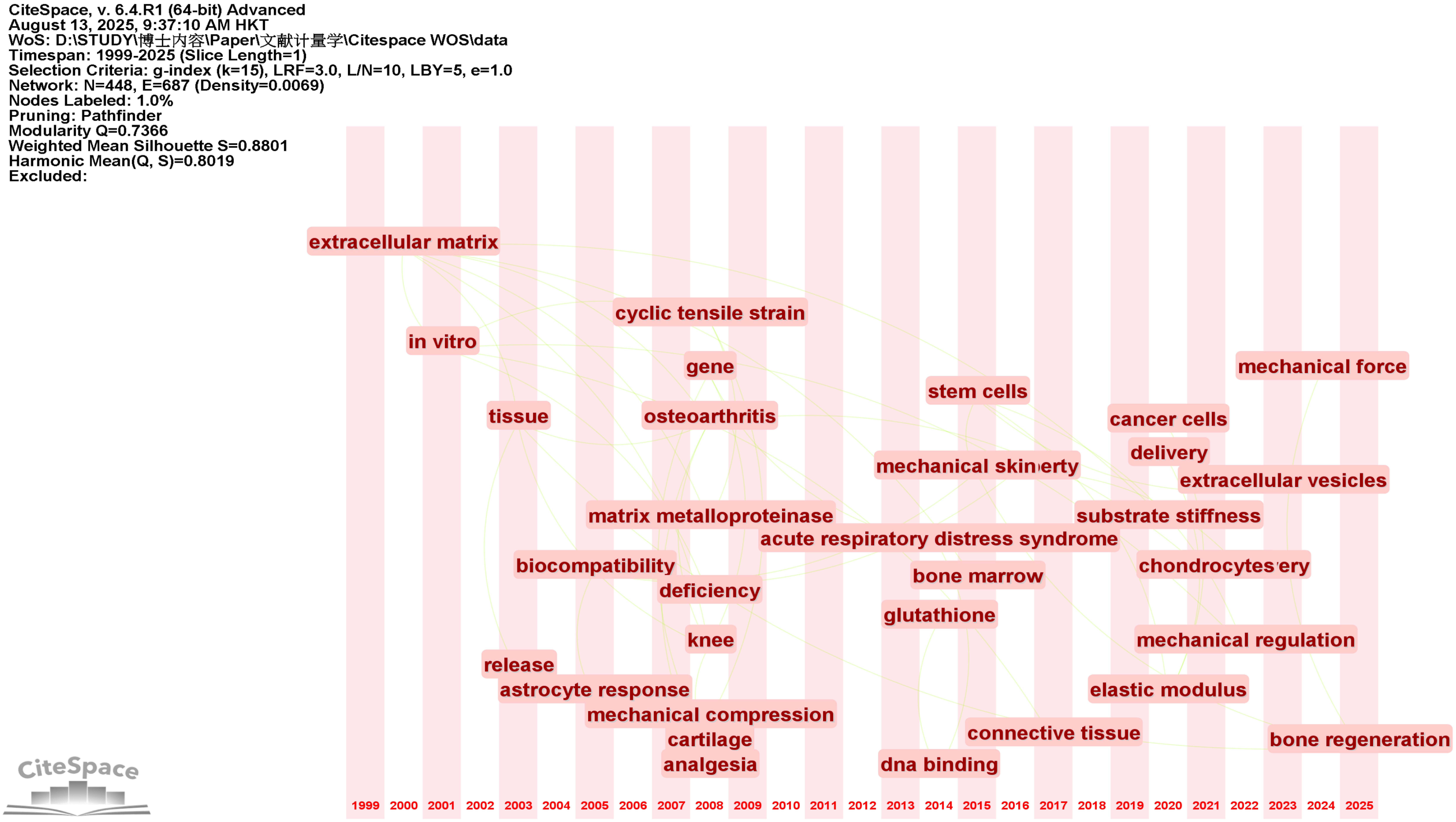

CiteSpace, v. 6.4.R1 (64-bit) Advanced
August 13, 2025, 9:37:10 AM HKT
WoS: D:\STUDY\博士内容\Paper\文献计量学\Citespace WOS\data
Timespan: 1999-2025 (Slice Length=1)
Selection Criteria: g-index (k=15), LRF=3.0, L/N=10, LBY=5, e=1.0
Network: N=448, E=687 (Density=0.0069)
Nodes Labeled: 1.0%
Pruning: Pathfinder
Modularity Q=0.7366
Weighted Mean Silhouette S=0.8801
Harmonic Mean(Q, S)=0.8019
Excluded:
extracellular matrix
cyclic tensile strain
in vitro
gene
mechanical force
stem cells
tissue
osteoarthritis
cancer cells
delivery
mechanical skinerty
extracellular vesicles
matrix metalloproteinase
substrate stiffness
acute respiratory distress syndrome
biocompatibility
chondrocytesery
bone marrow
deficiency
glutathione
knee
mechanical regulation
release
astrocyte response
elastic modulus
mechanical compression
connective tissue
cartilage
bone regeneration
analgesia
dna binding
1999
2000
2001
2002
2003
2004
2005
2006
2007
2008
2009
2010
2011
2012
2013
2014
2015
2016
2017
2018
2019
2020
2021
2022
2023
2024
2025
CiteSpace
