# Supplementary material for: Mechanical stress-mediated immune and inflammatory regulation: a bibliometric and visualization analysis of mechanoimmunology based on two databases
Source: Front Med (Lausanne). 2025 Nov 6;12:1698177. doi: 10.3389/fmed.2025.1698177 (PMC12631210; doi:10.3389/fmed.2025.1698177)
Supplement: Supplementary File S1 — Each cluster of all time. [file Data_Sheet_1.zip › each cluster of all time/cluster7.pdf]

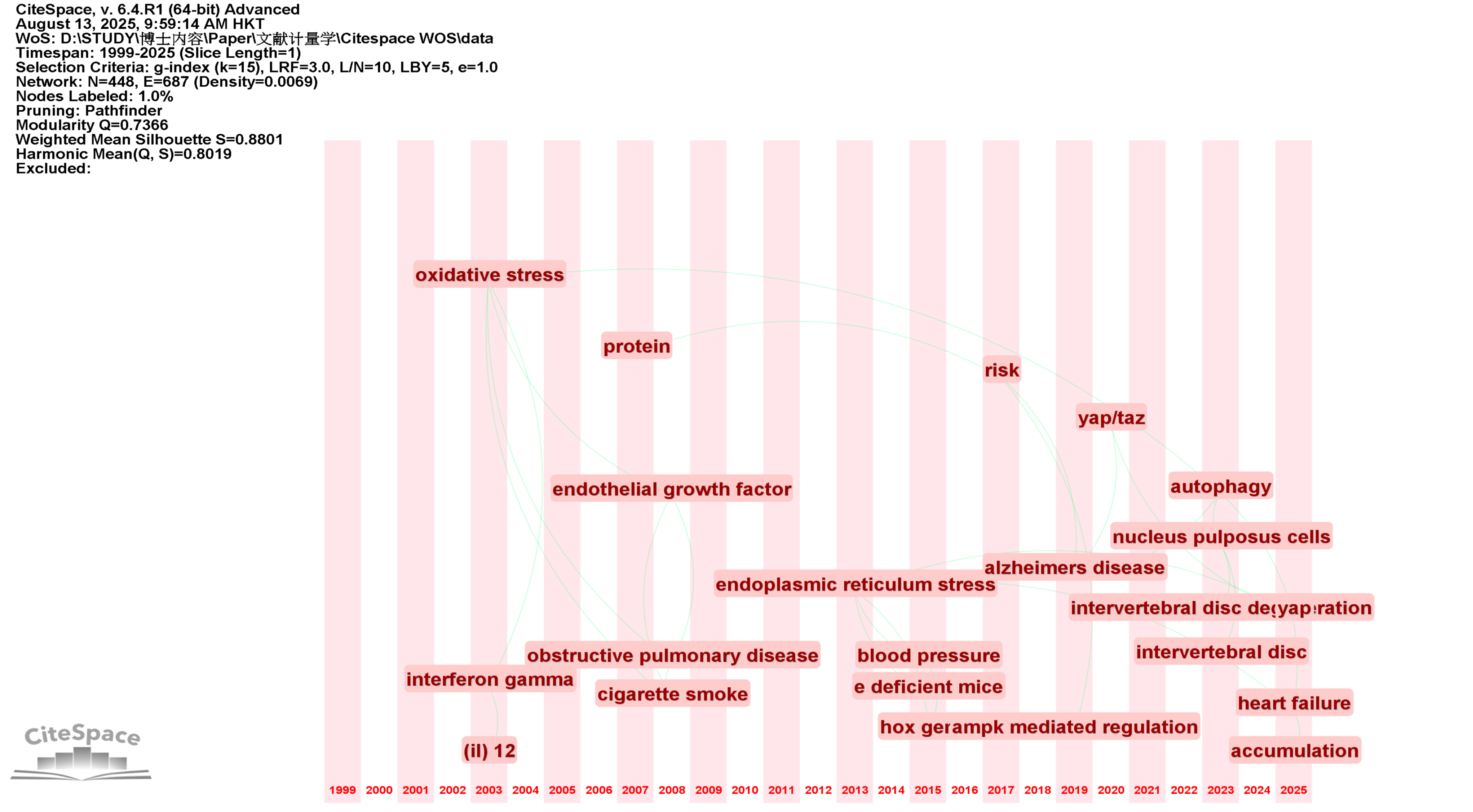
CiteSpace, v. 6.4.R1 (64-bit) Advanced
August 13, 2025, 9:59:14 AM HKT
WoS: D:\STUDY\博士内容\Paper\文献计量学\Citespace WOS\data
Timespan: 1999-2025 (Slice Length=1)
Selection Criteria: g-index (k=15), LRF=3.0, L/N=10, LBY=5, e=1.0
Network: N=448, E=687 (Density=0.0069)
Nodes Labeled: 1.0%
Pruning: Pathfinder
Modularity Q=0.7366
Weighted Mean Silhouette S=0.8801
Harmonic Mean(Q, S)=0.8019
Excluded:
oxidative stress
protein
risk
yap/taz
endothelial growth factor
autophagy
nucleus pulposus cells
alzheimers disease
endoplasmic reticulum stress
intervertebral disc de yap ration
obstructive pulmonary disease
blood pressure
intervertebral disc
interferon gamma
cigarette smoke
e deficient mice
heart failure
hox gerampk mediated regulation
(il) 12
accumulation
CiteSpace
1999 2000 2001 2002 2003 2004 2005 2006 2007 2008 2009 2010 2011 2012 2013 2014 2015 2016 2017 2018 2019 2020 2021 2022 2023 2024 2025
