# Supplementary material for: Mechanical stress-mediated immune and inflammatory regulation: a bibliometric and visualization analysis of mechanoimmunology based on two databases
Source: Front Med (Lausanne). 2025 Nov 6;12:1698177. doi: 10.3389/fmed.2025.1698177 (PMC12631210; doi:10.3389/fmed.2025.1698177)
Supplement: Supplementary File S1 — Each cluster of all time. [file Data_Sheet_1.zip › each cluster of all time/cluster8.pdf]

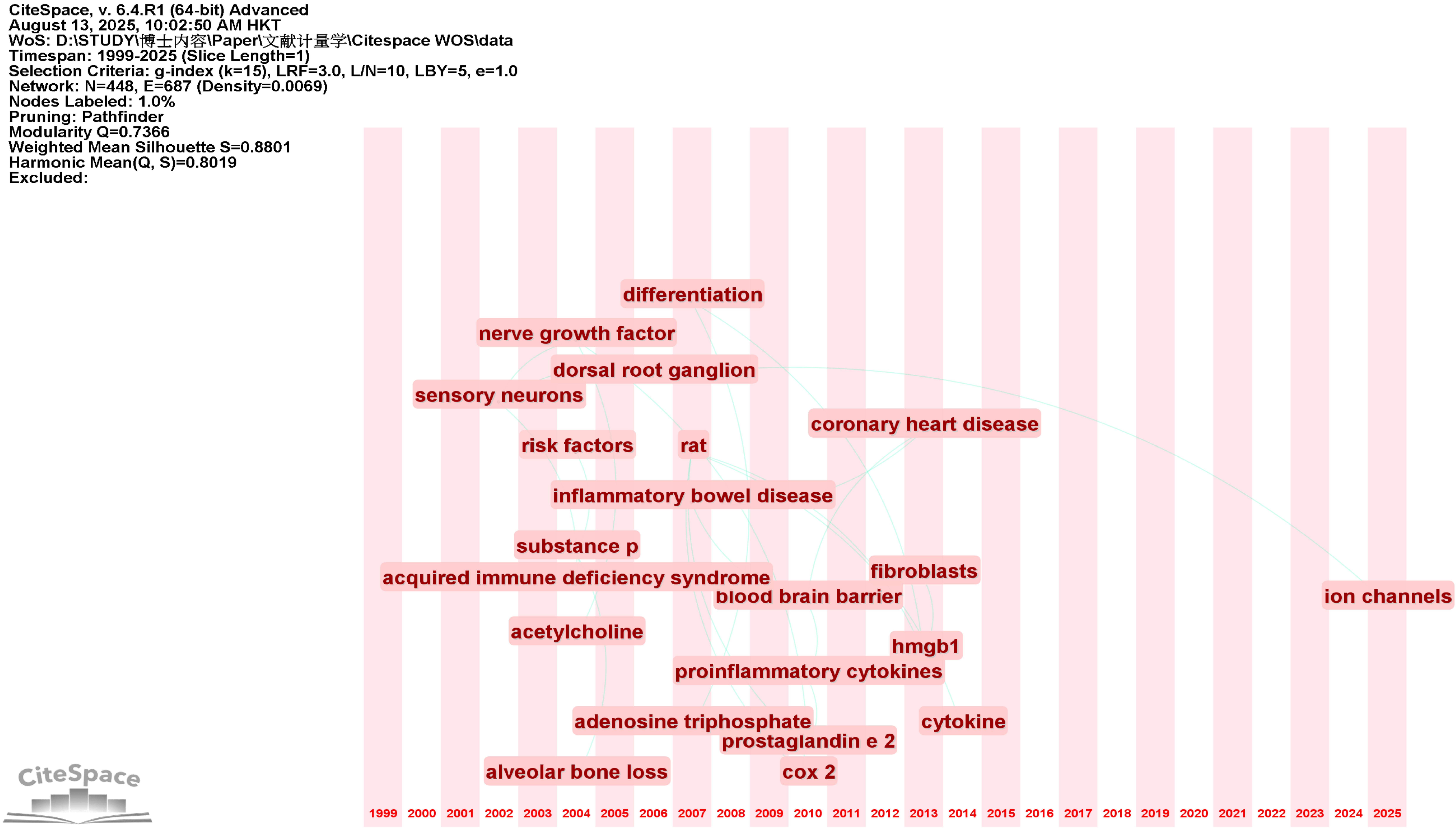
CiteSpace, v. 6.4.R1 (64-bit) Advanced
August 13, 2025, 10:02:50 AM HKT
WoS: D:\STUDY\博士内容\Paper\文献计量学\Citespace WOS\data
Timespan: 1999-2025 (Slice Length=1)
Selection Criteria: g-index (k=15), LRF=3.0, L/N=10, LBY=5, e=1.0
Network: N=448, E=687 (Density=0.0069)
Nodes Labeled: 1.0%
Pruning: Pathfinder
Modularity Q=0.7366
Weighted Mean Silhouette S=0.8801
Harmonic Mean(Q, S)=0.8019
Excluded:
differentiation
nerve growth factor
dorsal root ganglion
sensory neurons
coronary heart disease
risk factors
rat
inflammatory bowel disease
substance p
acquired immune deficiency syndrome
fibroblasts
blood brain barrier
ion channels
acetylcholine
hmgb1
proinflammatory cytokines
adenosine triphosphate
cytokine
prostaglandin e 2
alveolar bone loss
cox 2
CiteSpace
1999 2000 2001 2002 2003 2004 2005 2006 2007 2008 2009 2010 2011 2012 2013 2014 2015 2016 2017 2018 2019 2020 2021 2022 2023 2024 2025
