# Supplementary material for: Mechanical stress-mediated immune and inflammatory regulation: a bibliometric and visualization analysis of mechanoimmunology based on two databases
Source: Front Med (Lausanne). 2025 Nov 6;12:1698177. doi: 10.3389/fmed.2025.1698177 (PMC12631210; doi:10.3389/fmed.2025.1698177)
Supplement: Supplementary File S3 — Each cluster in exploration phase. [file Data_Sheet_3.zip › each cluster in exploration phase/cluster10.pdf]

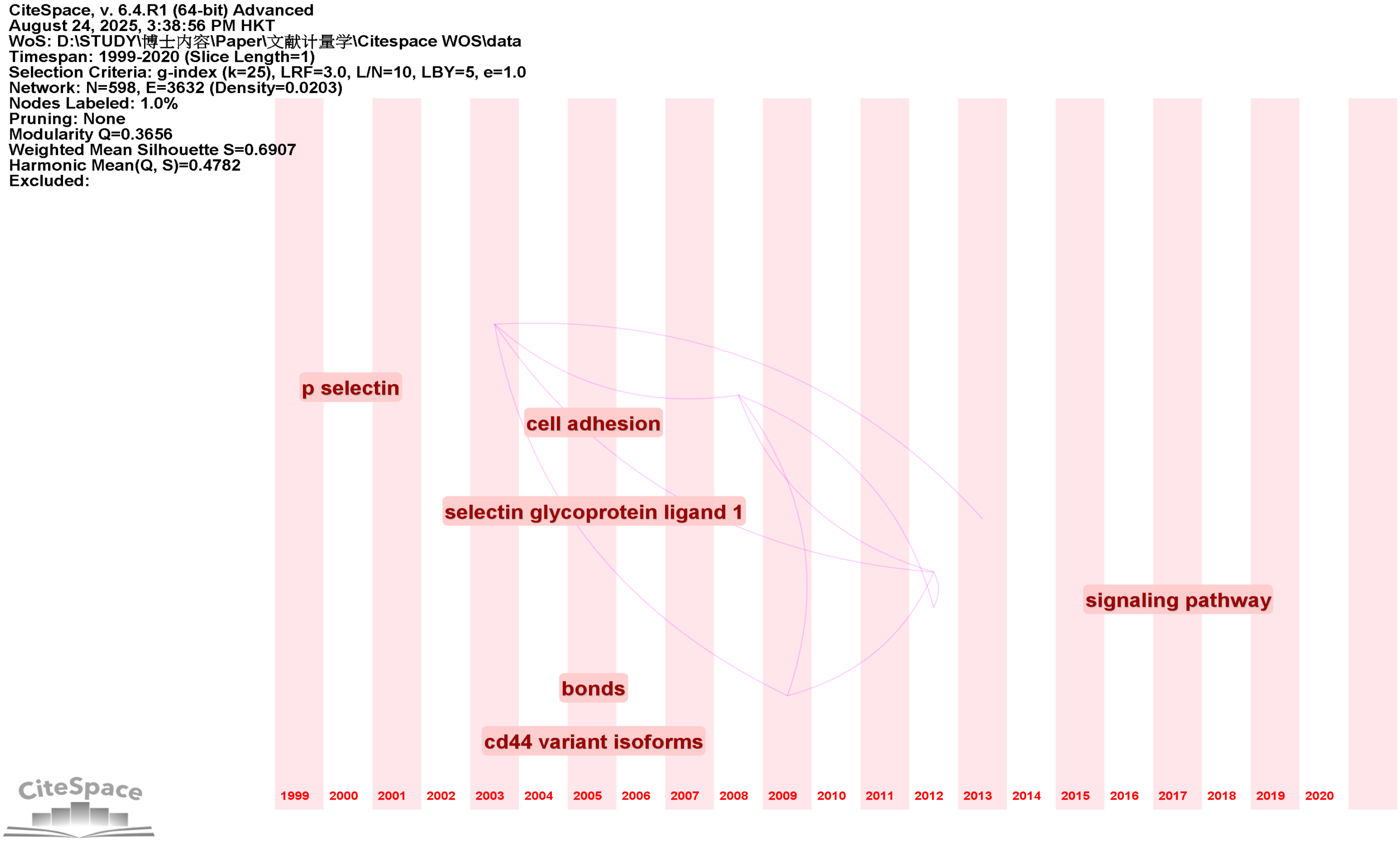
CiteSpace, v. 6.4.R1 (64-bit) Advanced
August 24, 2025, 3:38:56 PM HKT
WoS: D:\STUDY\博士内容\Paper\文献计量学\Citespace WOS\data
Timespan: 1999-2020 (Slice Length=1)
Selection Criteria: g-index (k=25), LRF=3.0, L/N=10, LBY=5, e=1.0
Network: N=598, E=3632 (Density=0.0203)
Nodes Labeled: 1.0%
Pruning: None
Modularity Q=0.3656
Weighted Mean Silhouette S=0.6907
Harmonic Mean(Q, S)=0.4782
Excluded:
p selectin
cell adhesion
selectin glycoprotein ligand 1
signaling pathway
bonds
cd44 variant isoforms
1999
2000
2001
2002
2003
2004
2005
2006
2007
2008
2009
2010
2011
2012
2013
2014
2015
2016
2017
2018
2019
2020
CiteSpace
