# Supplementary material for: Mechanical stress-mediated immune and inflammatory regulation: a bibliometric and visualization analysis of mechanoimmunology based on two databases
Source: Front Med (Lausanne). 2025 Nov 6;12:1698177. doi: 10.3389/fmed.2025.1698177 (PMC12631210; doi:10.3389/fmed.2025.1698177)
Supplement: Supplementary File S3 — Each cluster in exploration phase. [file Data_Sheet_3.zip › each cluster in exploration phase/cluster3.pdf]

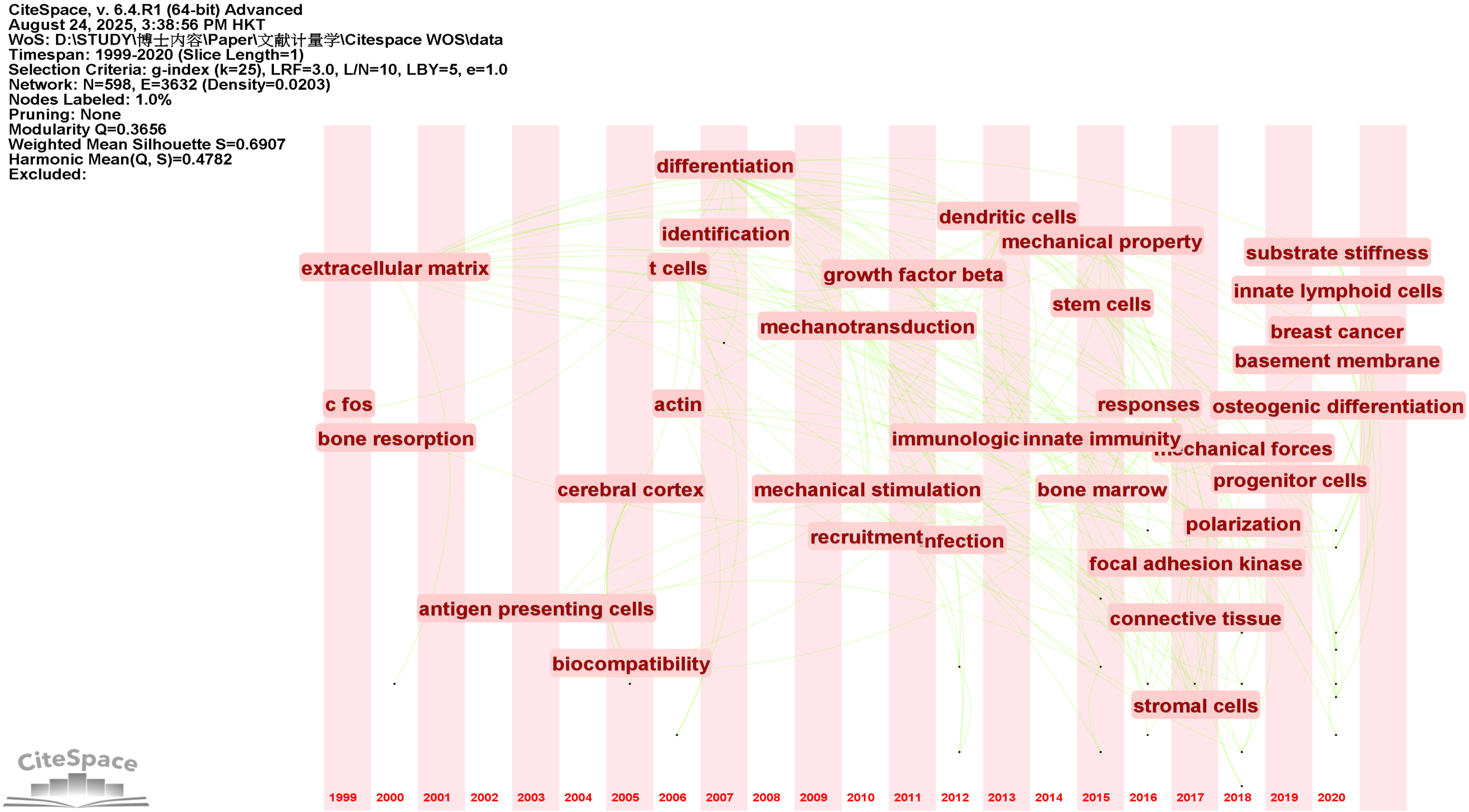
CiteSpace, v. 6.4.R1 (64-bit) Advanced
August 24, 2025, 3:38:56 PM HKT
WoS: D:\STUDY\博士内容\Paper\文献计量学\Citespace WOS\data
Timespan: 1999-2020 (Slice Length=1)
Selection Criteria: g-index (k=25), LRF=3.0, L/N=10, LBY=5, e=1.0
Network: N=598, E=3632 (Density=0.0203)
Nodes Labeled: 1.0%
Pruning: None
Modularity Q=0.3656
Weighted Mean Silhouette S=0.6907
Harmonic Mean(Q, S)=0.4782
Excluded:
differentiation
dendritic cells
identification
mechanical property
substrate stiffness
extracellular matrix
t cells
growth factor beta
innate lymphoid cells
stem cells
mechanotransduction
breast cancer
basement membrane
c fos
actin
responses
osteogenic differentiation
bone resorption
immunologic
innate immunity
chanical forces
progenitor cells
cerebral cortex
mechanical stimulation
bone marrow
polarization
recruitment
nfection
focal adhesion kinase
antigen presenting cells
connective tissue
biocompatibility
stromal cells
CiteSpace
1999
2000
2001
2002
2003
2004
2005
2006
2007
2008
2009
2010
2011
2012
2013
2014
2015
2016
2017
2018
2019
2020
