# Supplementary material for: Mechanical stress-mediated immune and inflammatory regulation: a bibliometric and visualization analysis of mechanoimmunology based on two databases
Source: Front Med (Lausanne). 2025 Nov 6;12:1698177. doi: 10.3389/fmed.2025.1698177 (PMC12631210; doi:10.3389/fmed.2025.1698177)
Supplement: Supplementary File S3 — Each cluster in exploration phase. [file Data_Sheet_3.zip › each cluster in exploration phase/cluster4.pdf]

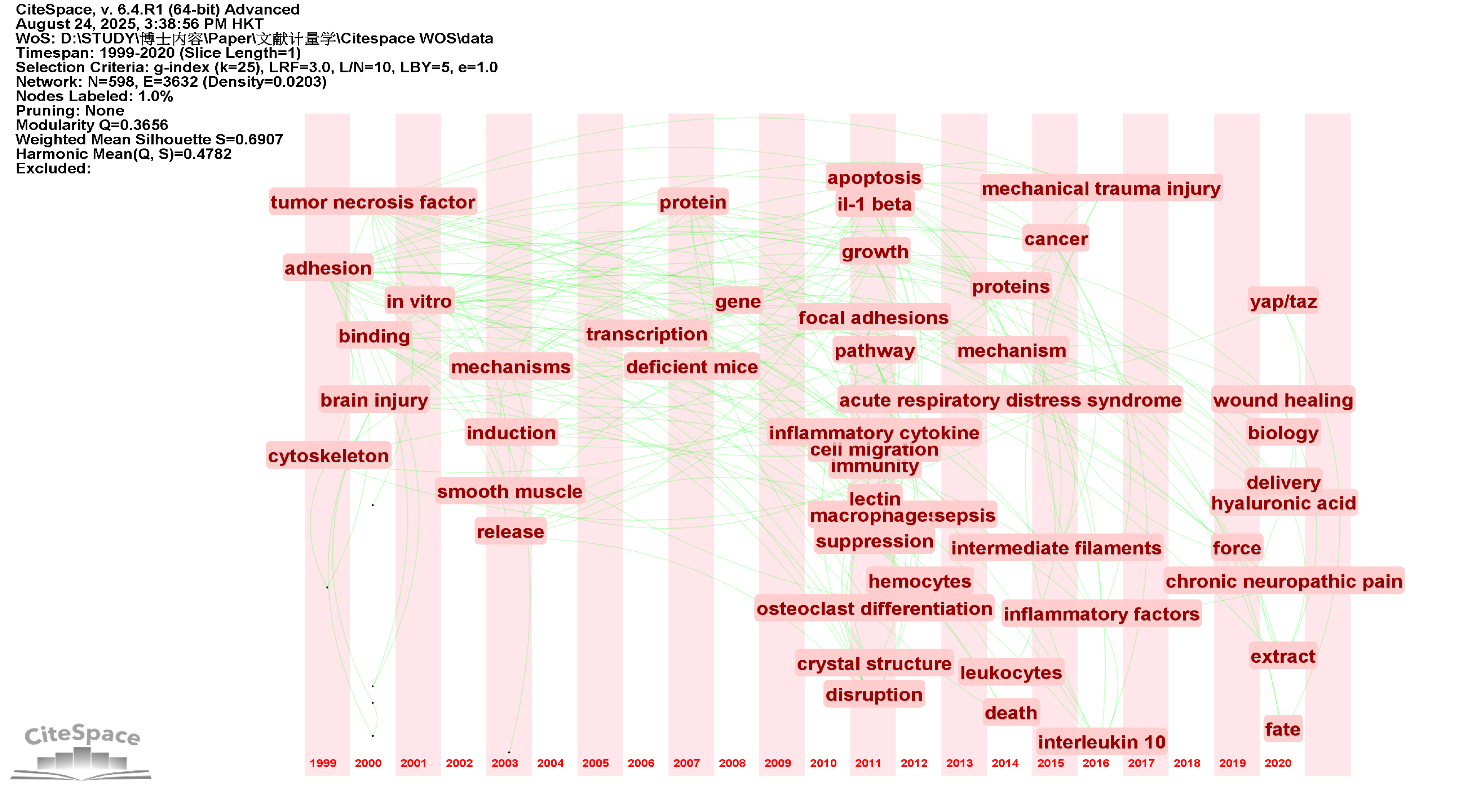
CiteSpace, v. 6.4.R1 (64-bit) Advanced
August 24, 2025, 3:38:56 PM HKT
WoS: D:\STUDY\博士内容\Paper\文献计量学\Citespace WOS\data
Timespan: 1999-2020 (Slice Length=1)
Selection Criteria: g-index (k=25), LRF=3.0, L/N=10, LBY=5, e=1.0
Network: N=598, E=3632 (Density=0.0203)
Nodes Labeled: 1.0%
Pruning: None
Modularity Q=0.3656
Weighted Mean Silhouette S=0.6907
Harmonic Mean(Q, S)=0.4782
Excluded:
tumor necrosis factor
adhesion
in vitro
binding
brain injury
cytoskeleton
mechanisms
induction
smooth muscle
release
protein
transcription
deficient mice
gene
apoptosis
il-1 beta
growth
focal adhesions
pathway
acute respiratory distress syndrome
inflammatory cytokine
cell migration
immunity
lectin
macrophages
sepsis
suppression
hemocytes
osteoclast differentiation
crystal structure
disruption
mechanical trauma injury
cancer
proteins
mechanism
intermediate filaments
inflammatory factors
leukocytes
death
interleukin 10
yap/taz
wound healing
biology
delivery
hyaluronic acid
force
chronic neuropathic pain
extract
fate
1999
2000
2001
2002
2003
2004
2005
2006
2007
2008
2009
2010
2011
2012
2013
2014
2015
2016
2017
2018
2019
2020
CiteSpace
