# Supplementary material for: Mechanical stress-mediated immune and inflammatory regulation: a bibliometric and visualization analysis of mechanoimmunology based on two databases
Source: Front Med (Lausanne). 2025 Nov 6;12:1698177. doi: 10.3389/fmed.2025.1698177 (PMC12631210; doi:10.3389/fmed.2025.1698177)
Supplement: Supplementary File S3 — Each cluster in exploration phase. [file Data_Sheet_3.zip › each cluster in exploration phase/cluster5.pdf]

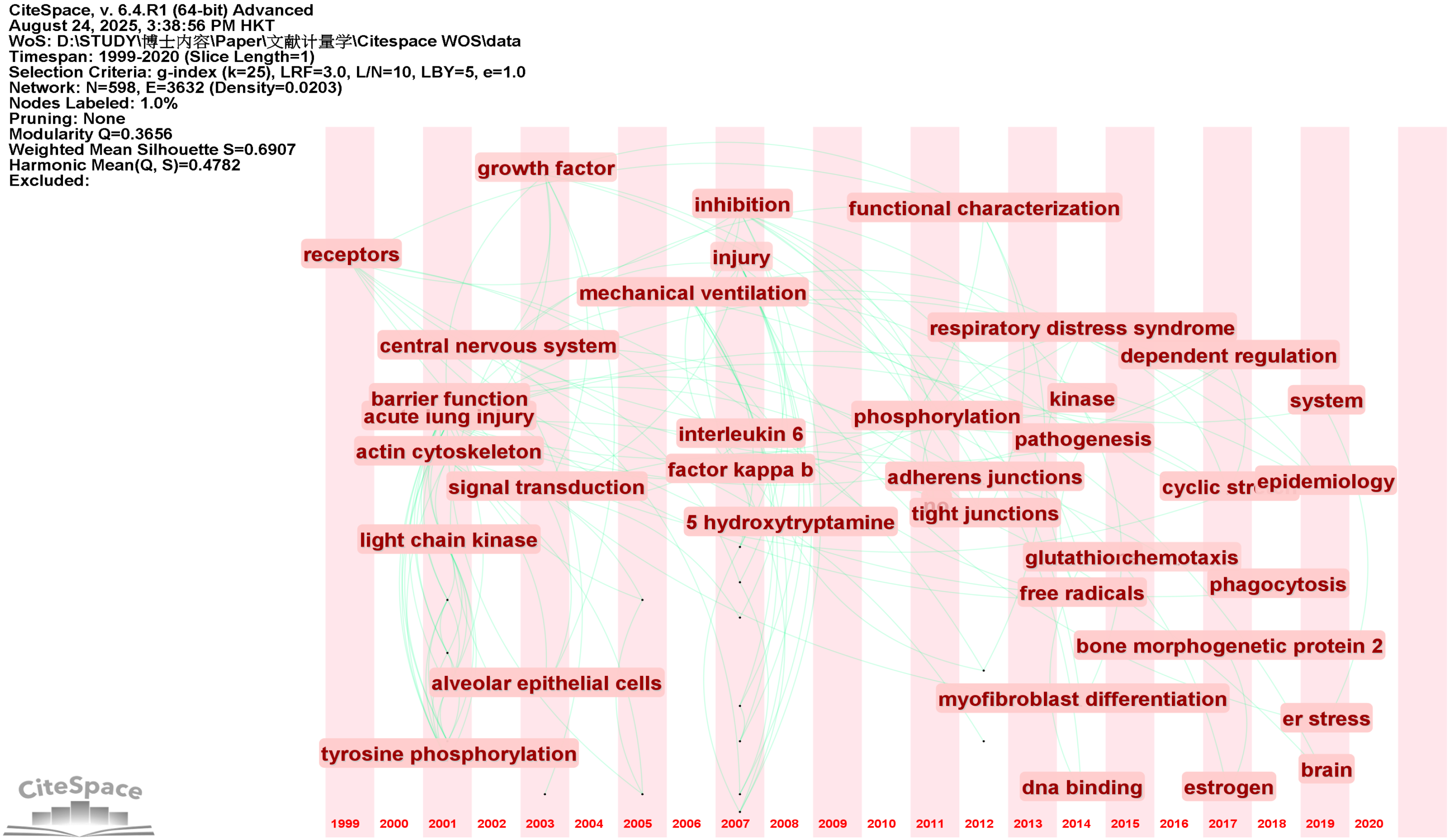
CiteSpace, v. 6.4.R1 (64-bit) Advanced
August 24, 2025, 3:38:56 PM HKT
WoS: D:\STUDY\博士内容\Paper\文献计量学\Citespace WOS\data
Timespan: 1999-2020 (Slice Length=1)
Selection Criteria: g-index (k=25), LRF=3.0, L/N=10, LBY=5, e=1.0
Network: N=598, E=3632 (Density=0.0203)
Nodes Labeled: 1.0%
Pruning: None
Modularity Q=0.3656
Weighted Mean Silhouette S=0.6907
Harmonic Mean(Q, S)=0.4782
Excluded:
growth factor
inhibition
functional characterization
receptors
injury
mechanical ventilation
respiratory distress syndrome
central nervous system
dependent regulation
barrier function
acute lung injury
kinase
system
phosphorylation
interleukin 6
pathogenesis
actin cytoskeleton
factor kappa b
adherens junctions
signal transduction
cyclic str
epidemiology
tight junctions
5 hydroxytryptamine
light chain kinase
glutathio
chemotaxis
phagocytosis
free radicals
bone morphogenetic protein 2
alveolar epithelial cells
myofibroblast differentiation
er stress
tyrosine phosphorylation
brain
dna binding
estrogen
CiteSpace
1999
2000
2001
2002
2003
2004
2005
2006
2007
2008
2009
2010
2011
2012
2013
2014
2015
2016
2017
2018
2019
2020
