# Supplementary material for: Mechanical stress-mediated immune and inflammatory regulation: a bibliometric and visualization analysis of mechanoimmunology based on two databases
Source: Front Med (Lausanne). 2025 Nov 6;12:1698177. doi: 10.3389/fmed.2025.1698177 (PMC12631210; doi:10.3389/fmed.2025.1698177)
Supplement: Supplementary File S3 — Each cluster in exploration phase. [file Data_Sheet_3.zip › each cluster in exploration phase/cluster6.pdf]

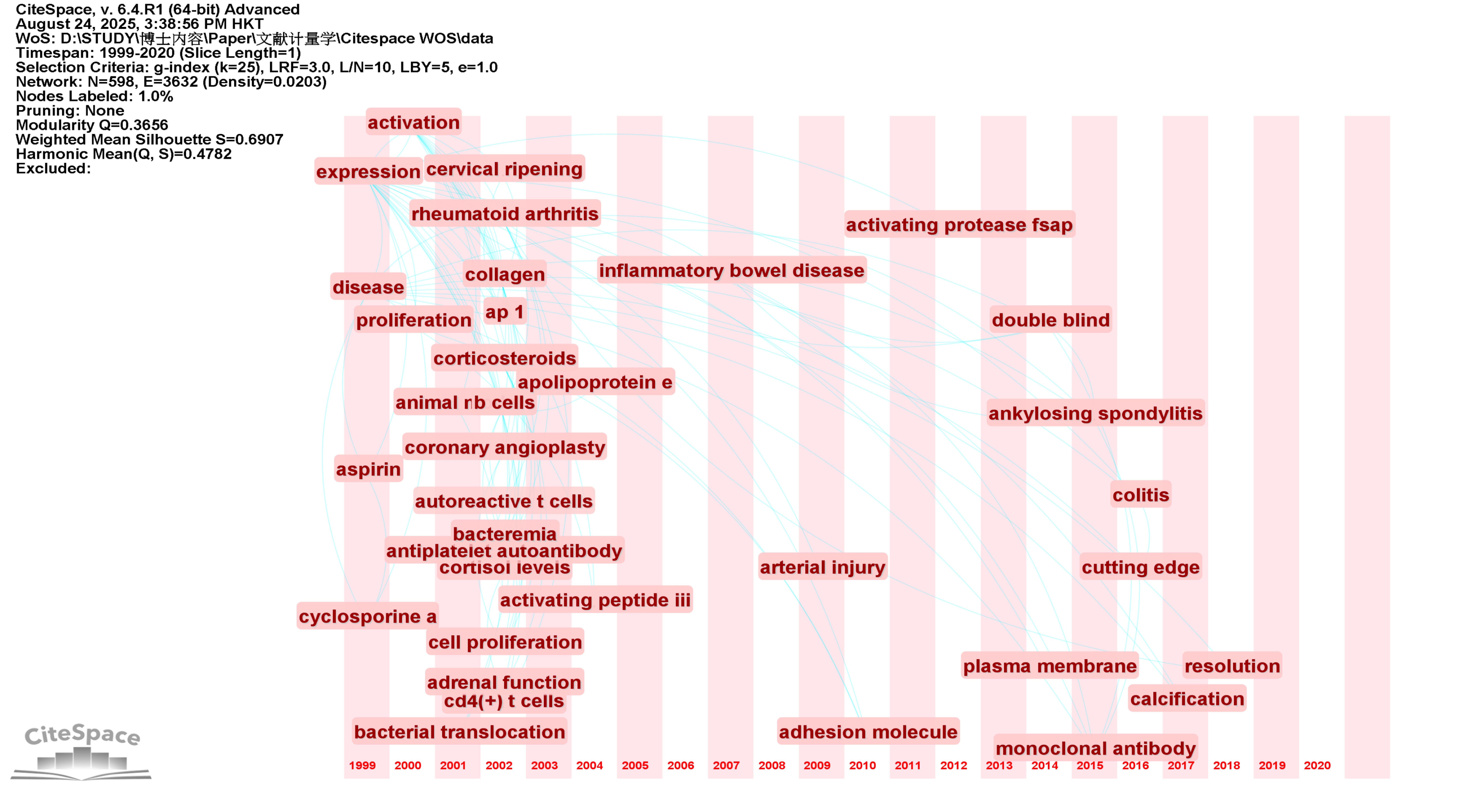
CiteSpace, v. 6.4.R1 (64-bit) Advanced
August 24, 2025, 3:38:56 PM HKT
WoS: D:\STUDY\博士内容\Paper\文献计量学\Citespace WOS\data
Timespan: 1999-2020 (Slice Length=1)
Selection Criteria: g-index (k=25), LRF=3.0, L/N=10, LBY=5, e=1.0
Network: N=598, E=3632 (Density=0.0203)
Nodes Labeled: 1.0%
Pruning: None
Modularity Q=0.3656
Weighted Mean Silhouette S=0.6907
Harmonic Mean(Q, S)=0.4782
Excluded:
activation
expression
cervical ripening
rheumatoid arthritis
activating protease fsap
inflammatory bowel disease
disease
collagen
ap 1
proliferation
double blind
corticosteroids
apolipoprotein e
animal rb cells
ankylosing spondylitis
coronary angioplasty
aspirin
autoreactive t cells
colitis
bacteremia
antiplatelet autoantibody
cortisol levels
arterial injury
cutting edge
activating peptide iii
cyclosporine a
cell proliferation
plasma membrane
resolution
adrenal function
cd4(+) t cells
calcification
bacterial translocation
adhesion molecule
monoclonal antibody
1999
2000
2001
2002
2003
2004
2005
2006
2007
2008
2009
2010
2011
2012
2013
2014
2015
2016
2017
2018
2019
2020
CiteSpace
