# Supplementary material for: Mechanical stress-mediated immune and inflammatory regulation: a bibliometric and visualization analysis of mechanoimmunology based on two databases
Source: Front Med (Lausanne). 2025 Nov 6;12:1698177. doi: 10.3389/fmed.2025.1698177 (PMC12631210; doi:10.3389/fmed.2025.1698177)
Supplement: Supplementary File S4 — Each cluster in explosion phase. [file Data_Sheet_4.zip › each cluster in explosion phase/cluster1.pdf]

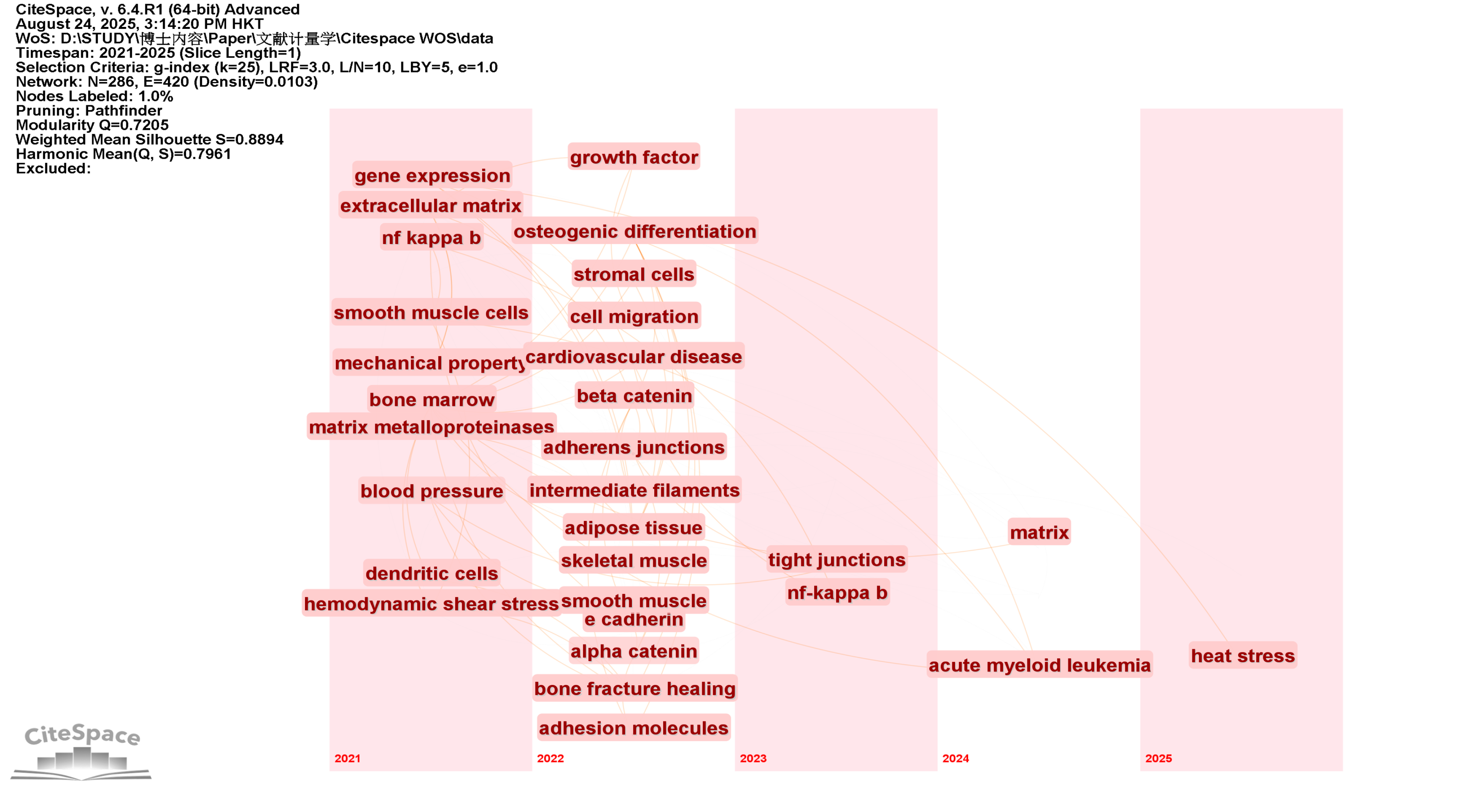
CiteSpace, v. 6.4.R1 (64-bit) Advanced
August 24, 2025, 3:14:20 PM HKT
WoS: D:\STUDY\博士内容\Paper\文献计量学\Citespace WOS\data
Timespan: 2021-2025 (Slice Length=1)
Selection Criteria: g-index (k=25), LRF=3.0, L/N=10, LBY=5, e=1.0
Network: N=286, E=420 (Density=0.0103)
Nodes Labeled: 1.0%
Pruning: Pathfinder
Modularity Q=0.7205
Weighted Mean Silhouette S=0.8894
Harmonic Mean(Q, S)=0.7961
Excluded:
gene expression
extracellular matrix
nf kappa b
smooth muscle cells
mechanical property
bone marrow
matrix metalloproteinases
blood pressure
dendritic cells
hemodynamic shear stress
growth factor
osteogenic differentiation
stromal cells
cell migration
cardiovascular disease
beta catenin
adherens junctions
intermediate filaments
adipose tissue
skeletal muscle
smooth muscle
e cadherin
alpha catenin
bone fracture healing
adhesion molecules
tight junctions
nf-kappa b
matrix
acute myeloid leukemia
heat stress
2021
2022
2023
2024
2025
CiteSpace
