# Supplementary material for: Mechanical stress-mediated immune and inflammatory regulation: a bibliometric and visualization analysis of mechanoimmunology based on two databases
Source: Front Med (Lausanne). 2025 Nov 6;12:1698177. doi: 10.3389/fmed.2025.1698177 (PMC12631210; doi:10.3389/fmed.2025.1698177)
Supplement: Supplementary File S4 — Each cluster in explosion phase. [file Data_Sheet_4.zip › each cluster in explosion phase/cluster2.pdf]

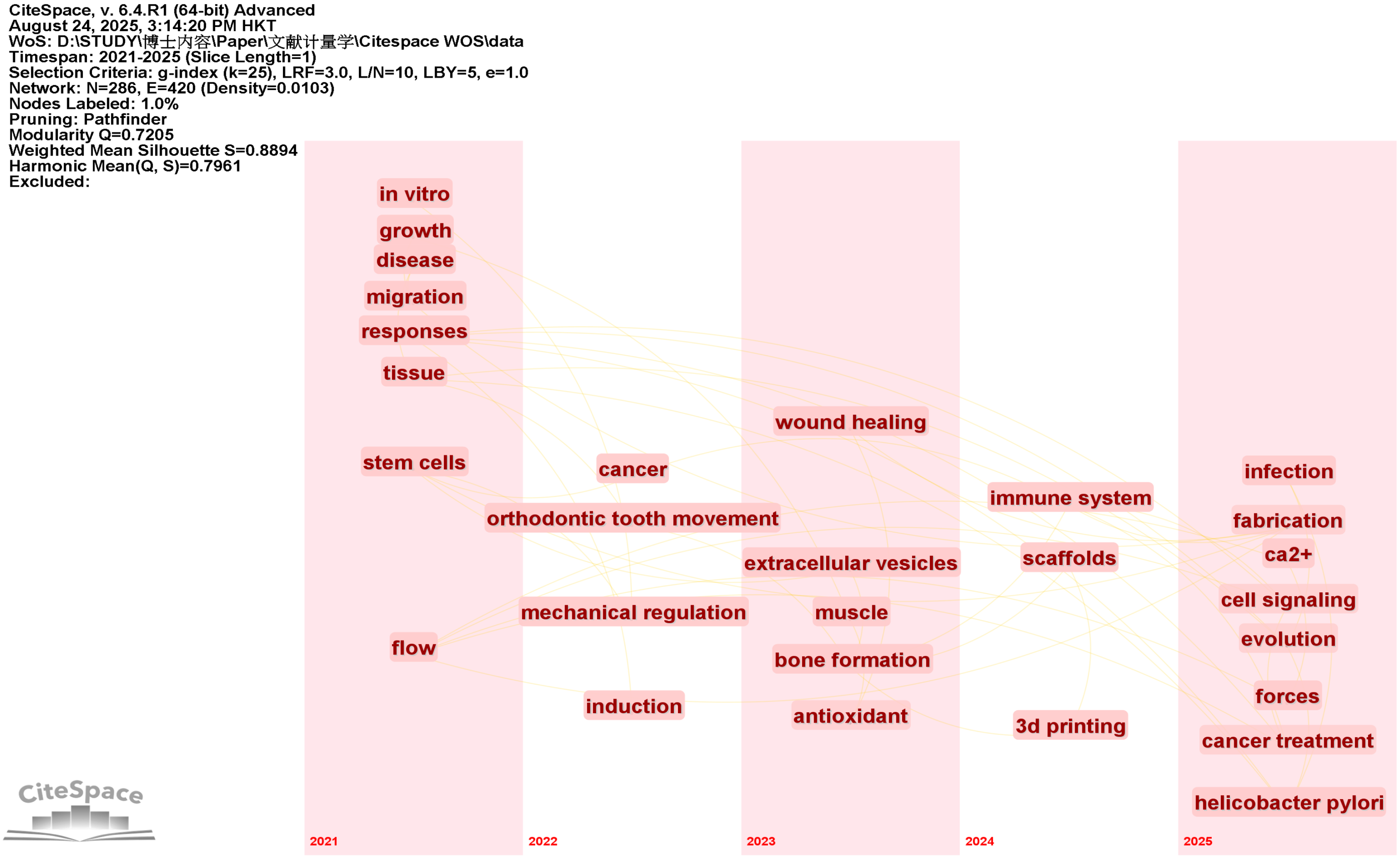
CiteSpace, v. 6.4.R1 (64-bit) Advanced
August 24, 2025, 3:14:20 PM HKT
WoS: D:\STUDY\博士内容\Paper\文献计量学\Citespace WOS\data
Timespan: 2021-2025 (Slice Length=1)
Selection Criteria: g-index (k=25), LRF=3.0, L/N=10, LBY=5, e=1.0
Network: N=286, E=420 (Density=0.0103)
Nodes Labeled: 1.0%
Pruning: Pathfinder
Modularity Q=0.7205
Weighted Mean Silhouette S=0.8894
Harmonic Mean(Q, S)=0.7961
Excluded:
in vitro
growth
disease
migration
responses
tissue
stem cells
flow
cancer
orthodontic tooth movement
mechanical regulation
induction
wound healing
extracellular vesicles
muscle
bone formation
antioxidant
immune system
scaffolds
3d printing
infection
fabrication
ca2+
cell signaling
evolution
forces
cancer treatment
helicobacter pylori
2021
2022
2023
2024
2025
CiteSpace
