# Supplementary material for: Mechanical stress-mediated immune and inflammatory regulation: a bibliometric and visualization analysis of mechanoimmunology based on two databases
Source: Front Med (Lausanne). 2025 Nov 6;12:1698177. doi: 10.3389/fmed.2025.1698177 (PMC12631210; doi:10.3389/fmed.2025.1698177)
Supplement: Supplementary File S4 — Each cluster in explosion phase. [file Data_Sheet_4.zip › each cluster in explosion phase/cluster8.pdf]

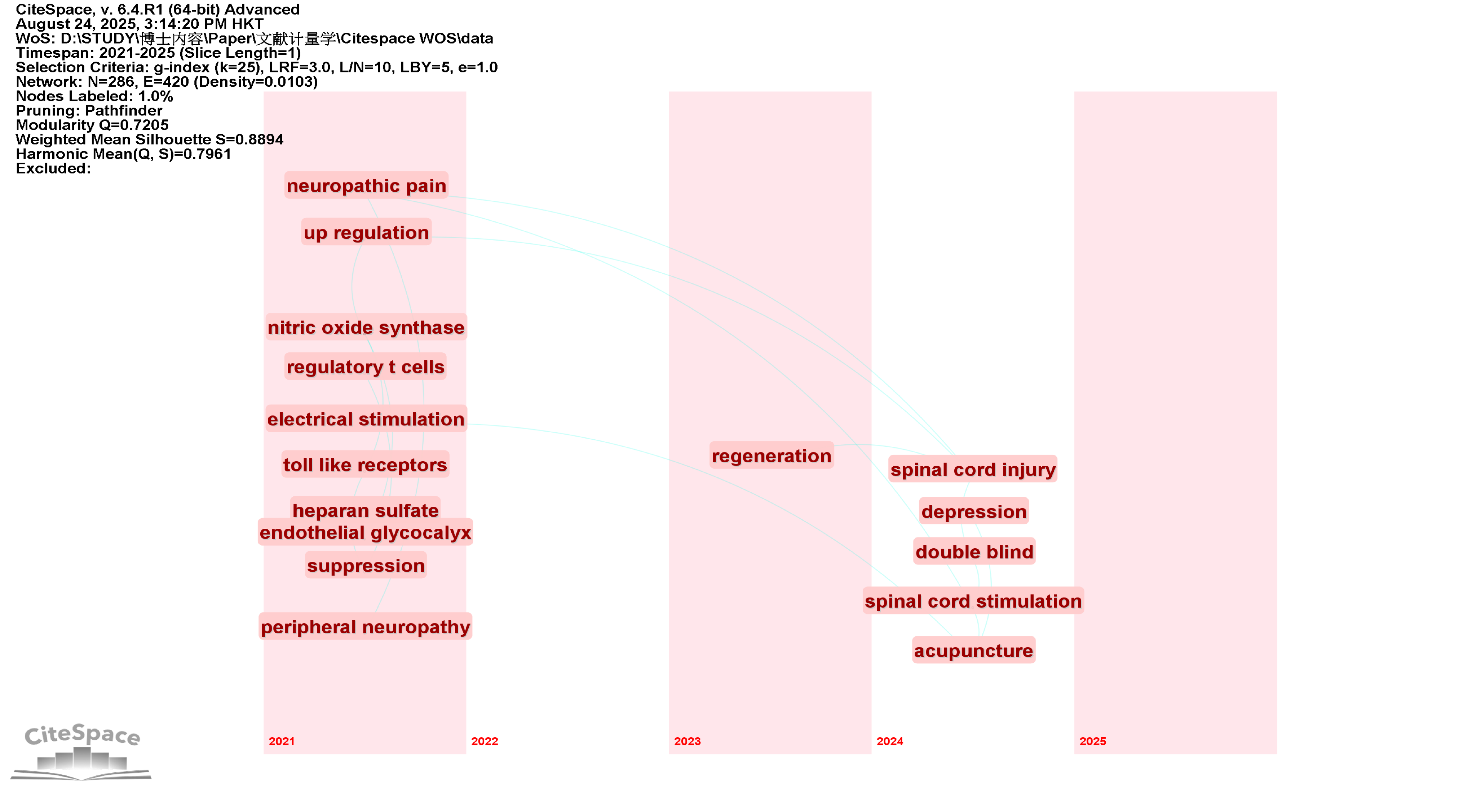
CiteSpace, v. 6.4.R1 (64-bit) Advanced
August 24, 2025, 3:14:20 PM HKT
WoS: D:\STUDY\博士内容\Paper\文献计量学\Citespace WOS\data
Timespan: 2021-2025 (Slice Length=1)
Selection Criteria: g-index (k=25), LRF=3.0, L/N=10, LBY=5, e=1.0
Network: N=286, E=420 (Density=0.0103)
Nodes Labeled: 1.0%
Pruning: Pathfinder
Modularity Q=0.7205
Weighted Mean Silhouette S=0.8894
Harmonic Mean(Q, S)=0.7961
Excluded:
neuropathic pain
up regulation
nitric oxide synthase
regulatory t cells
electrical stimulation
toll like receptors
heparan sulfate
endothelial glycocalyx
suppression
peripheral neuropathy
regeneration
spinal cord injury
depression
double blind
spinal cord stimulation
acupuncture
2021
2022
2023
2024
2025
CiteSpace
