# Supplementary material for: Mechanical stress-mediated immune and inflammatory regulation: a bibliometric and visualization analysis of mechanoimmunology based on two databases
Source: Front Med (Lausanne). 2025 Nov 6;12:1698177. doi: 10.3389/fmed.2025.1698177 (PMC12631210; doi:10.3389/fmed.2025.1698177)
Supplement: Supplementary File S3 — Each cluster in exploration phase. [file cluster0.pdf › each cluster in exploration phase/cluster0.pdf]

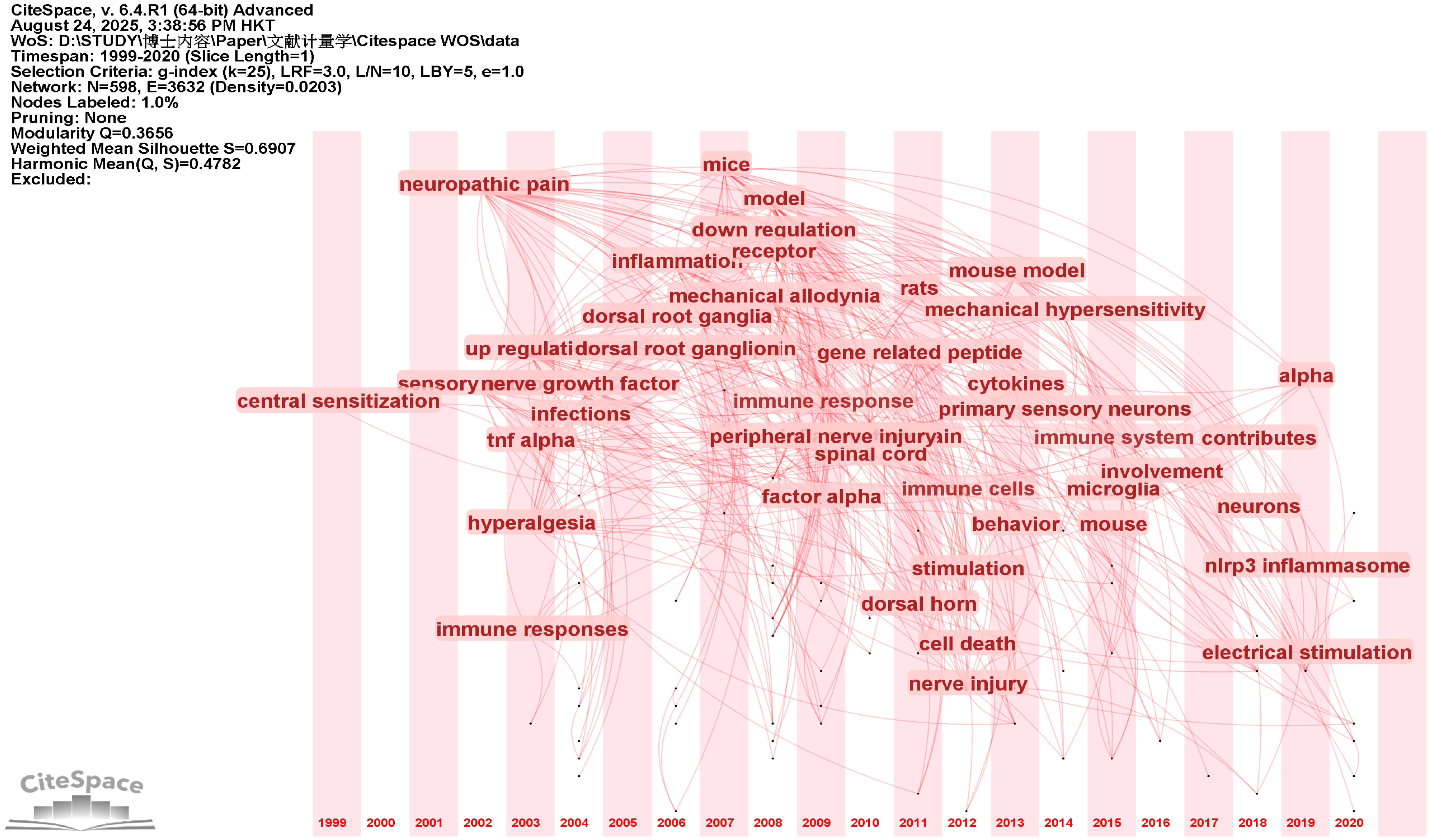
CiteSpace, v. 6.4.R1 (64-bit) Advanced
August 24, 2025, 3:38:56 PM HKT
WoS: D:\STUDY\博士内容\Paper\文献计量学\Citespace WOS\data
Timespan: 1999-2020 (Slice Length=1)
Selection Criteria: g-index (k=25), LRF=3.0, L/N=10, LBY=5, e=1.0
Network: N=598, E=3632 (Density=0.0203)
Nodes Labeled: 1.0%
Pruning: None
Modularity Q=0.3656
Weighted Mean Silhouette S=0.6907
Harmonic Mean(Q, S)=0.4782
Excluded:
mice
neuropathic pain
model
down regulation
inflammation
receptor
mouse model
rats
mechanical allodynia
mechanical hypersensitivity
dorsal root ganglia
up regulati
dorsal root ganglion
gene related peptide
alpha
sensory
nerve growth factor
cytokines
central sensitization
immune response
primary sensory neurons
infections
tnf alpha
peripheral nerve injury
immune system
contributes
spinal cord
involvement
immune cells
microglia
factor alpha
neurons
hyperalgesia
behavior
mouse
stimulation
nlrp3 inflammasome
dorsal horn
immune responses
cell death
electrical stimulation
nerve injury
CiteSpace
1999
2000
2001
2002
2003
2004
2005
2006
2007
2008
2009
2010
2011
2012
2013
2014
2015
2016
2017
2018
2019
2020
