# Supplementary material for: Mechanical stress-mediated immune and inflammatory regulation: a bibliometric and visualization analysis of mechanoimmunology based on two databases
Source: Front Med (Lausanne). 2025 Nov 6;12:1698177. doi: 10.3389/fmed.2025.1698177 (PMC12631210; doi:10.3389/fmed.2025.1698177)
Supplement: Supplementary File S3 — Each cluster in exploration phase. [file cluster2.pdf › each cluster in exploration phase/cluster2.pdf]

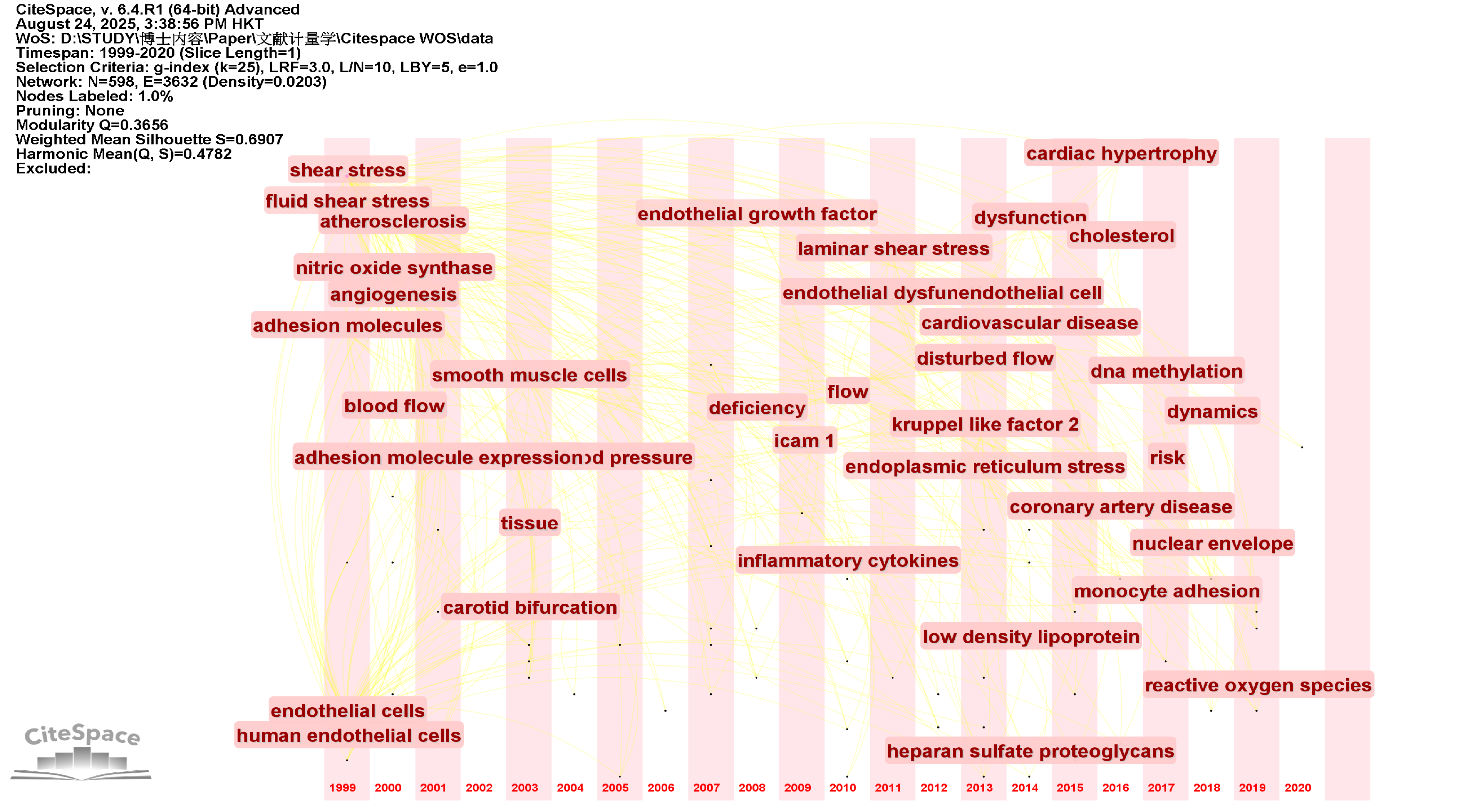
CiteSpace, v. 6.4.R1 (64-bit) Advanced
August 24, 2025, 3:38:56 PM HKT
WoS: D:\STUDY\博士内容\Paper\文献计量学\Citespace WOS\data
Timespan: 1999-2020 (Slice Length=1)
Selection Criteria: g-index (k=25), LRF=3.0, L/N=10, LBY=5, e=1.0
Network: N=598, E=3632 (Density=0.0203)
Nodes Labeled: 1.0%
Pruning: None
Modularity Q=0.3656
Weighted Mean Silhouette S=0.6907
Harmonic Mean(Q, S)=0.4782
Excluded:
shear stress
fluid shear stress
atherosclerosis
nitric oxide synthase
angiogenesis
adhesion molecules
smooth muscle cells
blood flow
adhesion molecule expression
d pressure
tissue
carotid bifurcation
endothelial cells
human endothelial cells
endothelial growth factor
laminar shear stress
endothelial dysfunendothelial cell
deficiency
flow
icam 1
inflammatory cytokines
cardiac hypertrophy
dysfunction
cholesterol
cardiovascular disease
disturbed flow
dna methylation
dynamics
kruppel like factor 2
risk
endoplasmic reticulum stress
coronary artery disease
nuclear envelope
monocyte adhesion
low density lipoprotein
reactive oxygen species
heparan sulfate proteoglycans
1999
2000
2001
2002
2003
2004
2005
2006
2007
2008
2009
2010
2011
2012
2013
2014
2015
2016
2017
2018
2019
2020
CiteSpace
